# Supplementary material for: Synthesis and Antimicrobial Studies of Coumarin-Substituted Pyrazole Derivatives as Potent Anti-Staphylococcus aureus Agents
Source: Molecules. 2020 Jun 15;25(12):2758. doi: 10.3390/molecules25122758 (PMC7356691; doi:10.3390/molecules25122758)

Synthesis and Antimicrobial Studies of Coumarin-Substituted Pyrazole Derivatives as Potent Anti-*Staphylococcus aureus* Agents

Rawan Alnufaie [1], Hansa Raj KC [1], Nickolas Alsup [1], Jedidiah Whitt [1], Steven Andrew Chambers [1], David Gilmore [2] and Mohammad A. Alam [1,*]

[1] Department of Chemistry and Physics, College of Science and Mathematics, Arkansas State University, Jonesboro, AR 72467, United States; rawan.alnufaie@smail.astate.edu (R.A.); hansa.kc@smail.astate.edu (H.R.K.C.); nickolas.alsup@smail.astate.edu (N.A); jedidiah.whitt@smail.astate.edu (J.W.); steven.chambers@smail.astate.edu (S.A.C.)

[2] Department of Biological Sciences, College of Science and Mathematics, Arkansas State University, Jonesboro, AR 72467, United States; dgilmore@astate.edu

* Correspondence: malam@astate.edu; Tel.: +1-870-972-3319 (M.A.A)

CHARACTERIZATION DATA

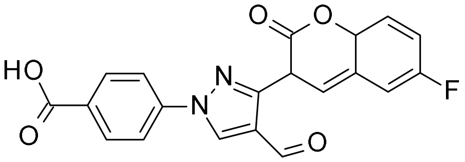

4-[3-(7-fluoro-2-oxo-3,8a-dihydrochromen-3-yl)-4-formyl-pyrazol-1-yl]benzoic acid (4). Brownish; (3.567 g, 93.7%) ^{1}H NMR (300MHz, DMSO-d$_6$): δ 9.91 (s, 1H), 9.35 (s, 1H), 8.68 (s, 1H), 8.09 (s, 4H), 7.72 (d, J = 8.0 Hz, 1H), 7.55-7.53 (m, 2H); ^{13}C NMR (75 MHz, DMSO-d$_6$): δ 186.0, 166.9, 158.6 (d, ^1J = 239.6 Hz), 159.5, 150.3, 148.0, 142.6, 141.8, 133.6, 131.4, 130.1, 124,2, 121.1, 120.4 (d, ^2J = 24.5 Hz), 120.1 (d, ^3J = 9.7 Hz), 119.4, 118.7 (d, ^3J = 8.7 Hz), 114.6 (d, ^2J = 24.3 Hz). HRMS (ESI-FTMS Mass (m/z): calcd for $C_{20}H_{11}FN_2O_5$ [M+H]$^+$ = 379.0725, found 379.0738.

^{1}H NMR of compound (**4**)

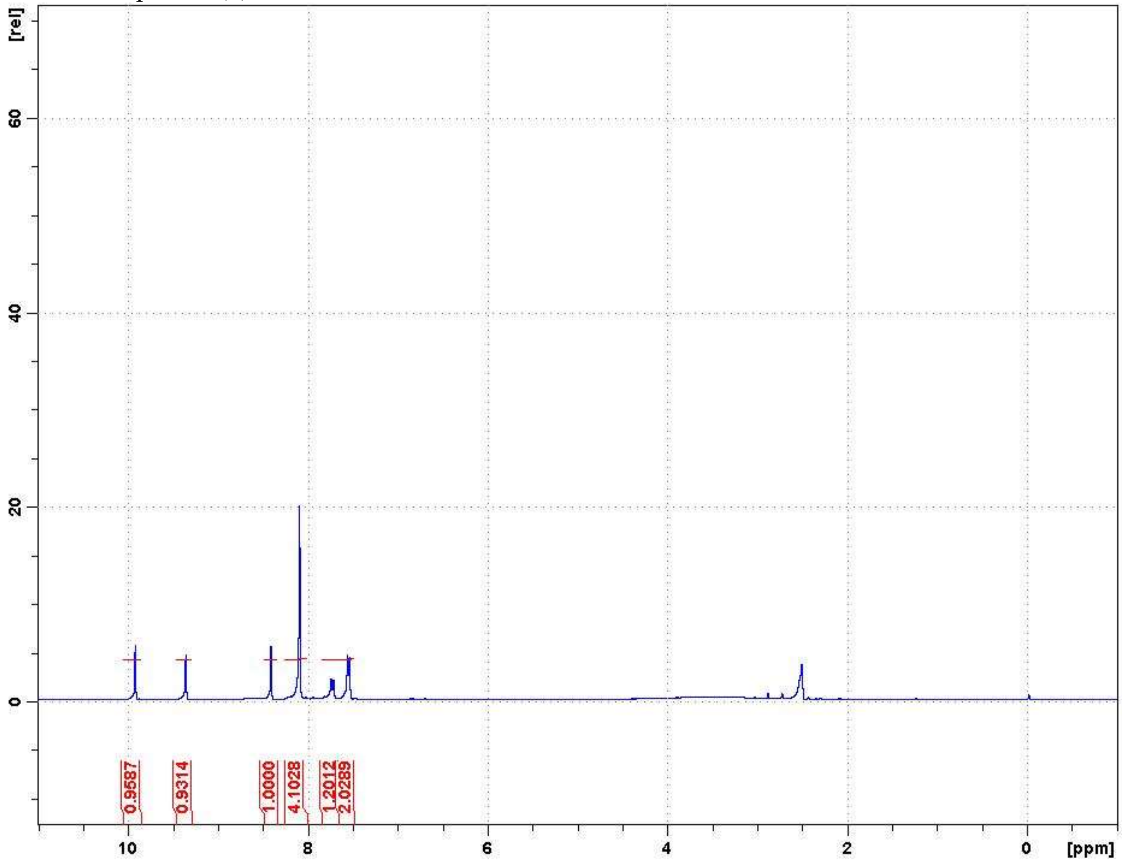

^{13}C NMR of compound (**4**)

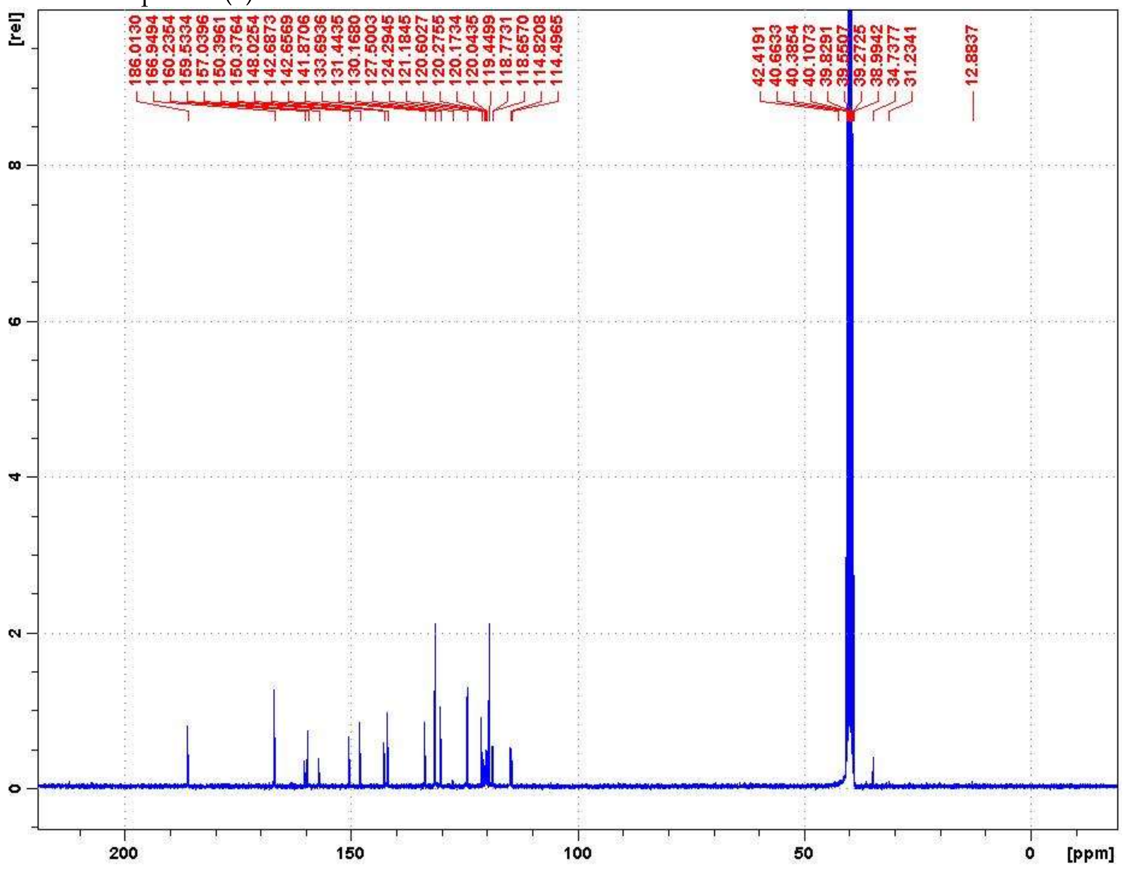

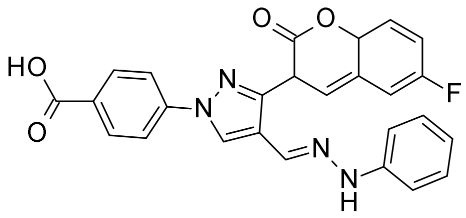

4-[3-(7-fluoro-2-oxo-3,8a-dihydrochromen-3-yl)-4-[(E)-(phenylhydrazono)methyl]pyrazol-1-yl]benzoic acid (5). Yellow; (343 mg, 73%) ^{1}H NMR (300MHz, DMSO-d$_6$): δ 10.22 (s, 1H), 8.98 (s, 1H), 8.32 (s, 1H), 8.12-8.05 (m, 4H), 7.84 (s, 1H), 7.74 (d, *J* = 9.3 Hz, 1H), 7.60-7.58 (m, Hz, 2H), 6.99 (t, *J* = 8.1 Hz, 2H), 6.75-6.73 (m, 2H), 6.63 (t, *J* = 7.2 Hz, 1H); ^{13}C NMR (75 MHz, DMSO-d$_6$): δ 167.0, 158.6 (d, ^{1}J = 239.3 Hz), 159.1, 150.2, 146.0, 145.6, 142.4, 141.9, 131.4, 129.2, 128.99, 128.96, 127.3, 123.3, 121.3, 120.3 (d, ^{3}J = 9.5 Hz), 120.0 (d, ^{2}J = 24.6 Hz), 118.8, 118.5 (d, ^{3}J = 8.5 Hz), 118.3, 114.4 (d, ^{2}J = 24.2 Hz), 112.0. HRMS (ESI-FTMS Mass (m/z): calcd for $C_{26}H_{20}N_4O_5$ $[M+H]^+$ = 469.1506, found 469.1298.

^{1}H NMR of compound (**5**)

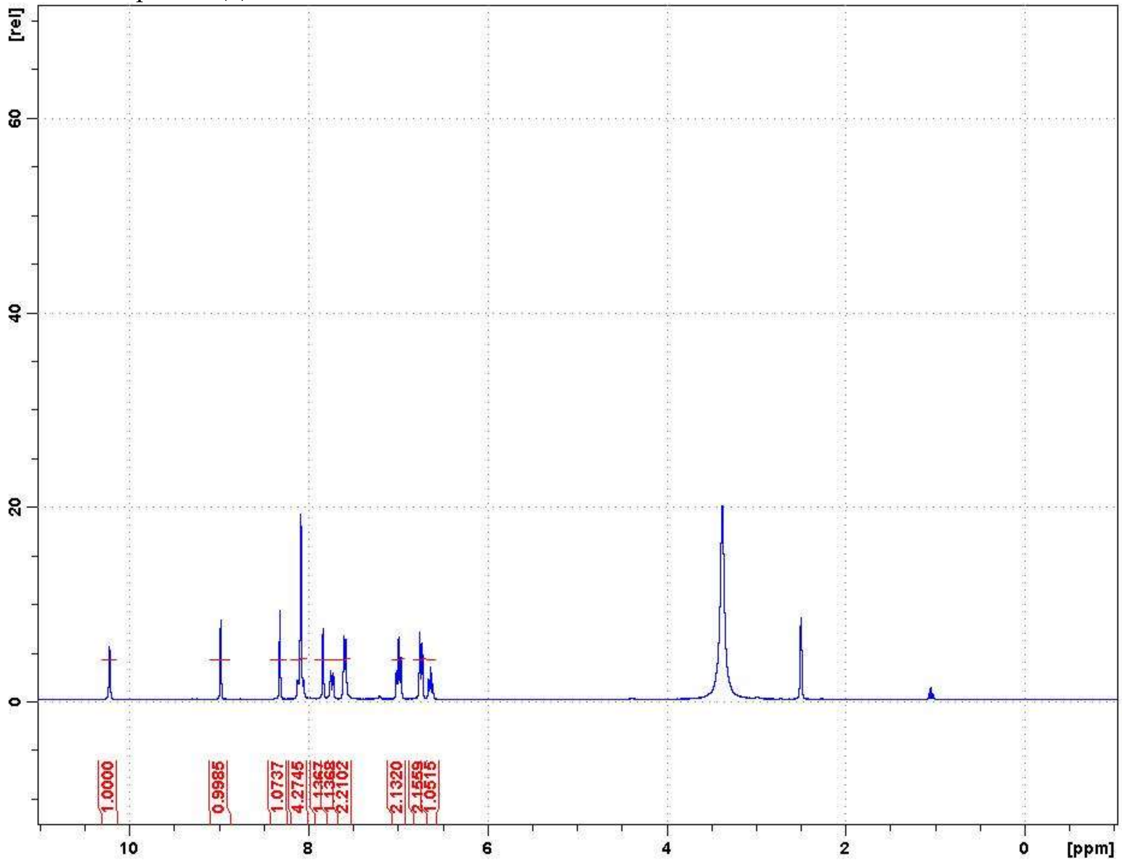

^{13}C NMR of compound (**5**)

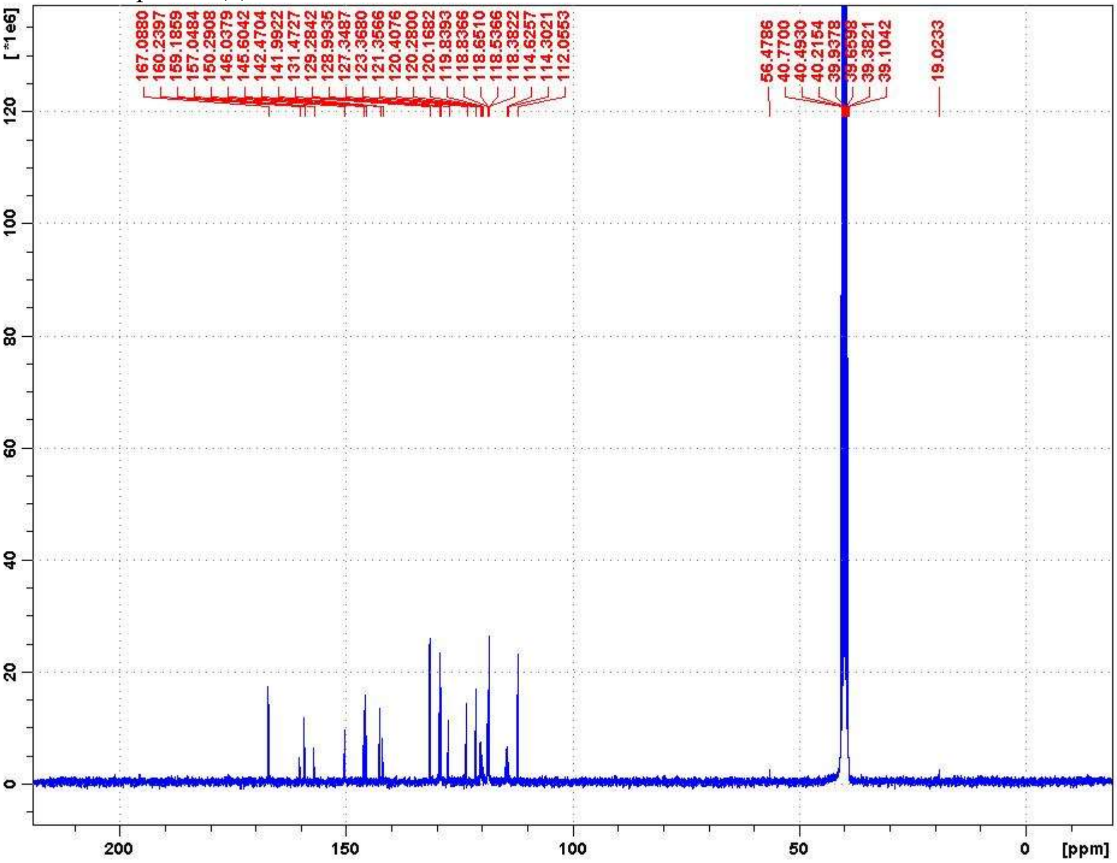

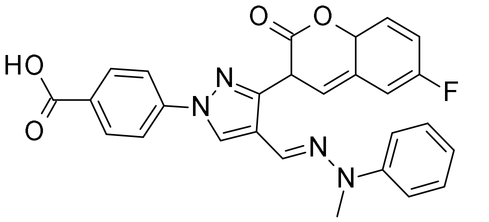

4-[3-(6-fluoro-2-oxo-3,8a-dihydrochromen-3-yl)-4-[(E)-[methyl(phenyl)hydrazono]methyl]pyrazol-1-yl]benzoic acid (6). Yellow; (373 mg, 77%) ^{1}H NMR (300MHz, DMSO-d$_6$): 8.95 (s, 1H), 8.32 (s, 1H), 8.09 (s, 4H), 7.76-7.72 (m, 1H), 7.62-7.56 (m, 3H), 7.10-7.07 (m, 2H), 6.98 (t, J = 7.3 Hz, 2H), 6.75 (t, J = 7.2 Hz, 1H), 2.50-2.49 (m, 3H); ^{13}C NMR (75 MHz, DMSO-d$_6$): δ 167.1, 158.6 (d, ^1J = 239.3 Hz), 159.2, 150.3, 147.5, 146.0, 142.4, 141.8, 131.4, 129.0, 128.9, 127.2, 125.4, 123.7, 122.2, 120.3 (d, ^3J = 9.5 Hz), 120.1, 119.7, 118.6 (d, ^3J = 8.5 Hz), 118.3, 114.5, 114.2, 32.7. HRMS (ESI-FTMS Mass (m/z): calcd for $C_{27}H_{19}FN_4O_4$ $[M+H]^+$ = 483.1463, found 483.1466.

^{1}H NMR of compound (**6**)

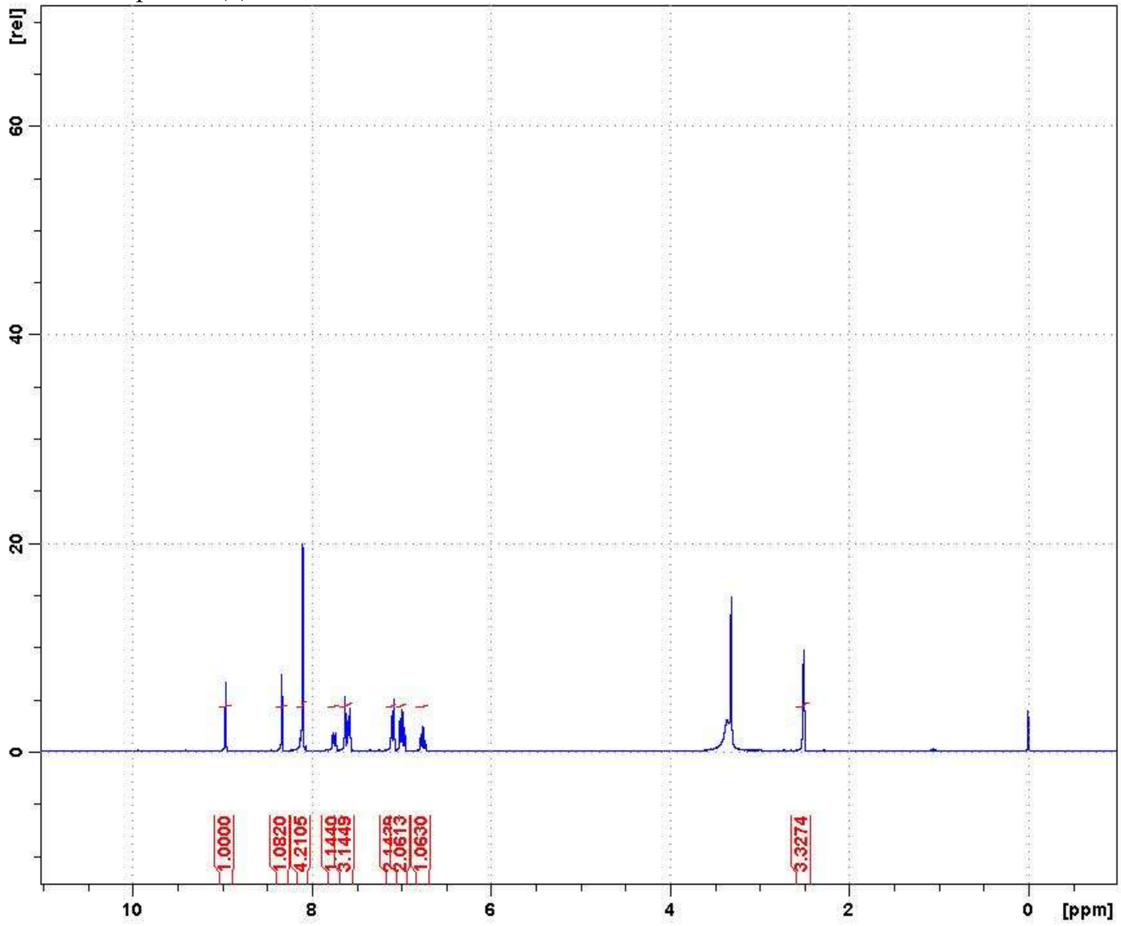

^{13}C NMR of compound (**6**)

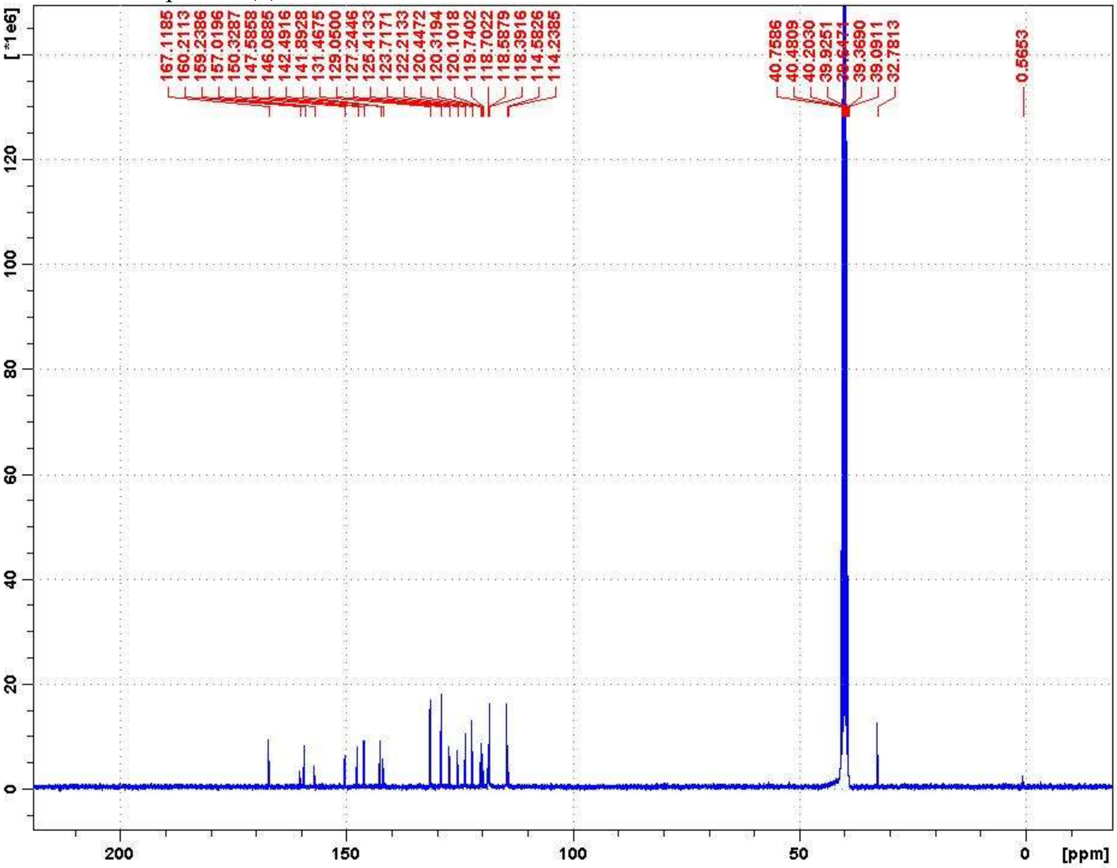

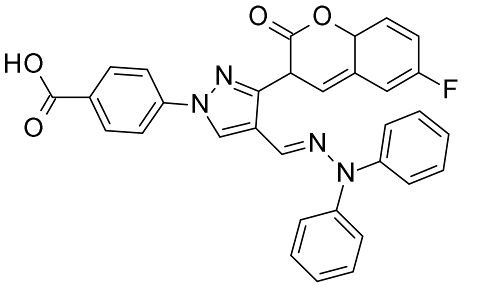

4-[4-[(E)-(diphenylhydrazono)methyl]-3-(6-fluoro-2-oxo-3,8a-dihydrochromen-3-yl)pyrazol-1-yl]benzoic acid (7). Yellow; (459 mg, 84%) ^{1}H NMR (300MHz, DMSO-d$_6$): δ 8.94 (s, 1H), 8.33 (s, 1H), 8.09-8.06 (m, 2H), 8.01-7.98 (m, 1H), 7.77-7.74 (m, 1H), 7.65-7.58 (m, 2H), 7.30-7.24 (m, 4H), 7.16-7.11 (m, 3H), 6.94-6.91 (m, 4H); ^{13}C NMR (75 MHz, DMSO-d$_6$): δ ^{13}C NMR (75 MHz, DMSO-d$_6$): δ 167.0, 158.6 (d, ^{1}J = 239.3 Hz) , 159.0, 150.2, 145.8, 143.1, 142.3, 141.9, 131.4, 130.2, 129.0, 128.6, 128.0, 124.9, 123.7, 122.2, 121.0, 120.2 (d, ^{3}J = 9.7 Hz), 119.8, 118.6 (d, ^{3}J = 8.7 Hz), 118.3, 144.4 (d, ^{2}J = 24.3 Hz). HRMS (ESI-FTMS Mass (m/z): calcd for $C_{32}H_{21}FN_4O_4$ [M+H]$^+$ = 545.1620, found 545.1613.

^{1}H NMR of compound (**7**)

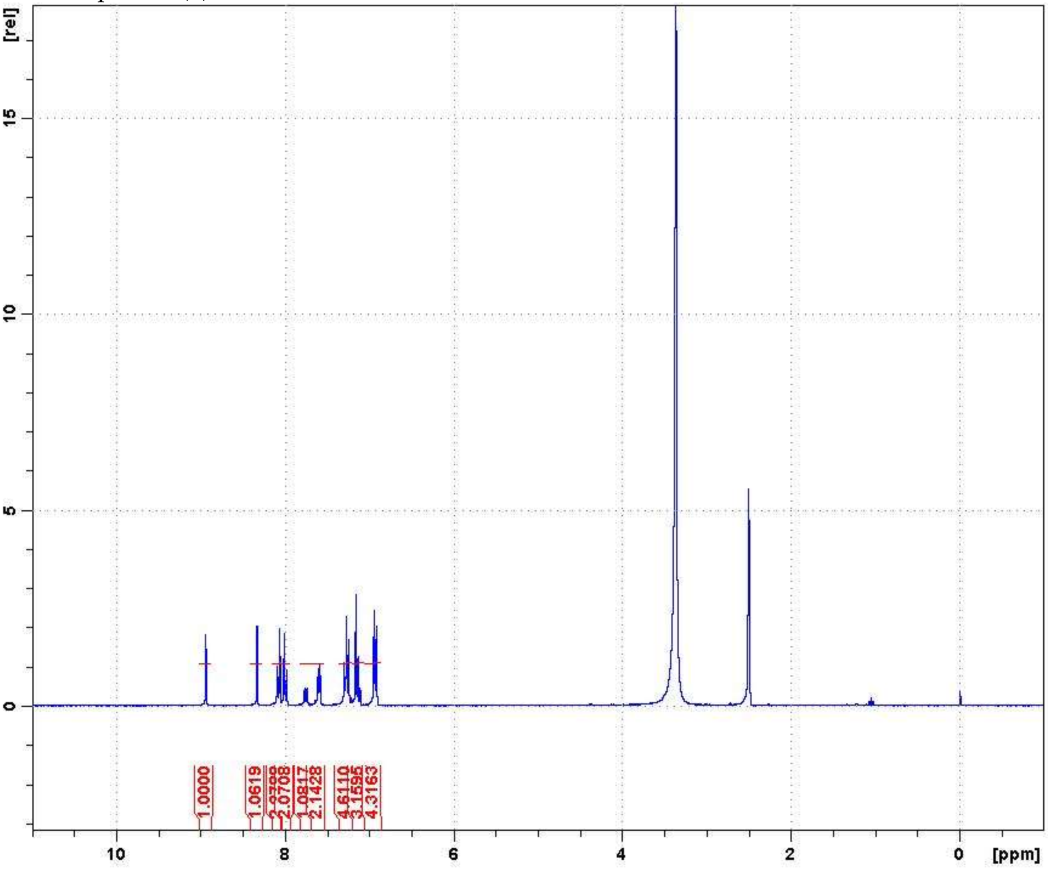

^{13}C NMR of compound (**7**)

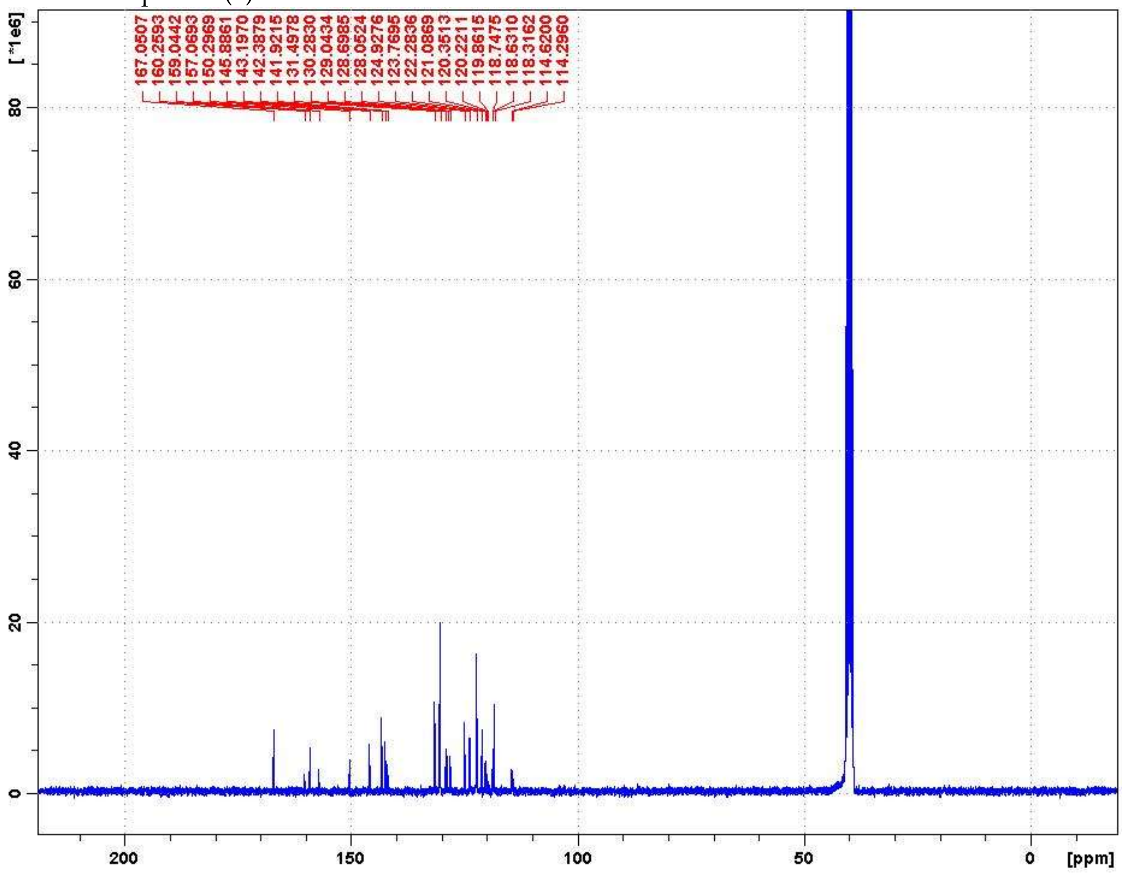

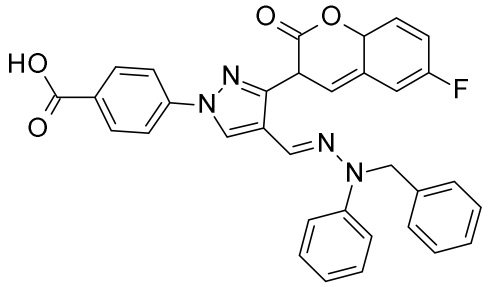

4-[4-[(E)-[benzyl(phenyl)hydrazono]methyl]-3-(6-fluoro-2-oxo-3,8a-dihydrochromen-3-yl)pyrazol-1-yl]benzoic acid (8). Yellow; (459 mg, 82%)^1H NMR (300MHz, DMSO-d$_6$): δ 8.88 (s, 1H), 8.28 (s, 1H), 8.09-8.00 (m, 4H), 7.73-7.71 (m, 1H), 7.58 (s, 3H), 7.53-7.12 (m, 5H), 6.99-6.86 (m, 4H), 6.75-6.71 (m, 1H), 5.20 (s, 2H); ^{13}C NMR (75 MHz, DMSO-d$_6$): δ ^{13}C NMR (75 MHz, DMSO-d$_6$): δ 167.0, 158.6 (d, ^1J = 239.4 Hz), 159.0, 150.2, 147.2, 145.9, 142.4, 141.7, 136.2, 131.4, 129.1, 128.9, 127.8, 127.4, 126.6, 125.3, 123.9, 121.8, 120.3, 120.2, 119.9 (d, ^2J = 24.6 Hz), 118.6 (d, ^3J = 8.7 Hz), 118.3, 114.4 (d, ^2J = 24.1 Hz), 113.9. 48.2. HRMS (ESI-FTMS Mass (m/z): calcd for $C_{33}H_{23}FN_4O_4$ $[M+H]^+$ = 559.1776, found 559.1768.

¹H NMR of compound (**8**)

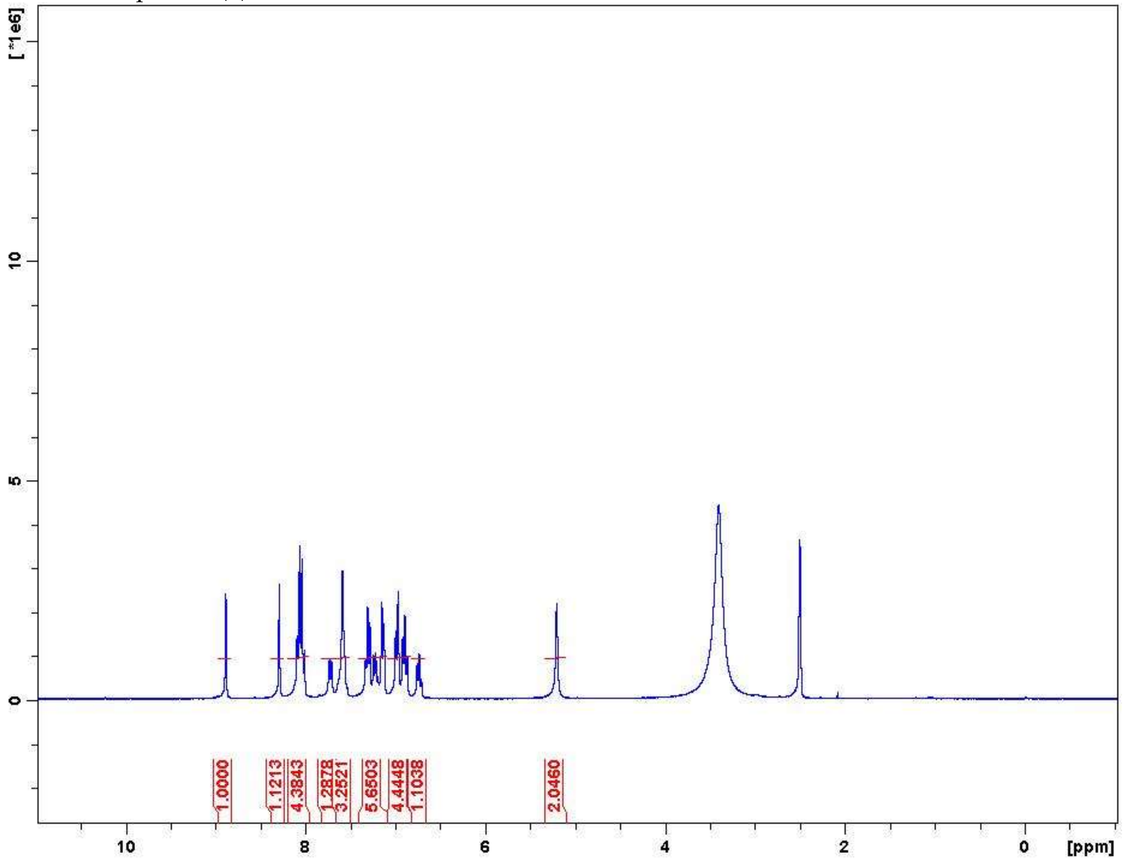

^{13}C NMR of compound (**8**)

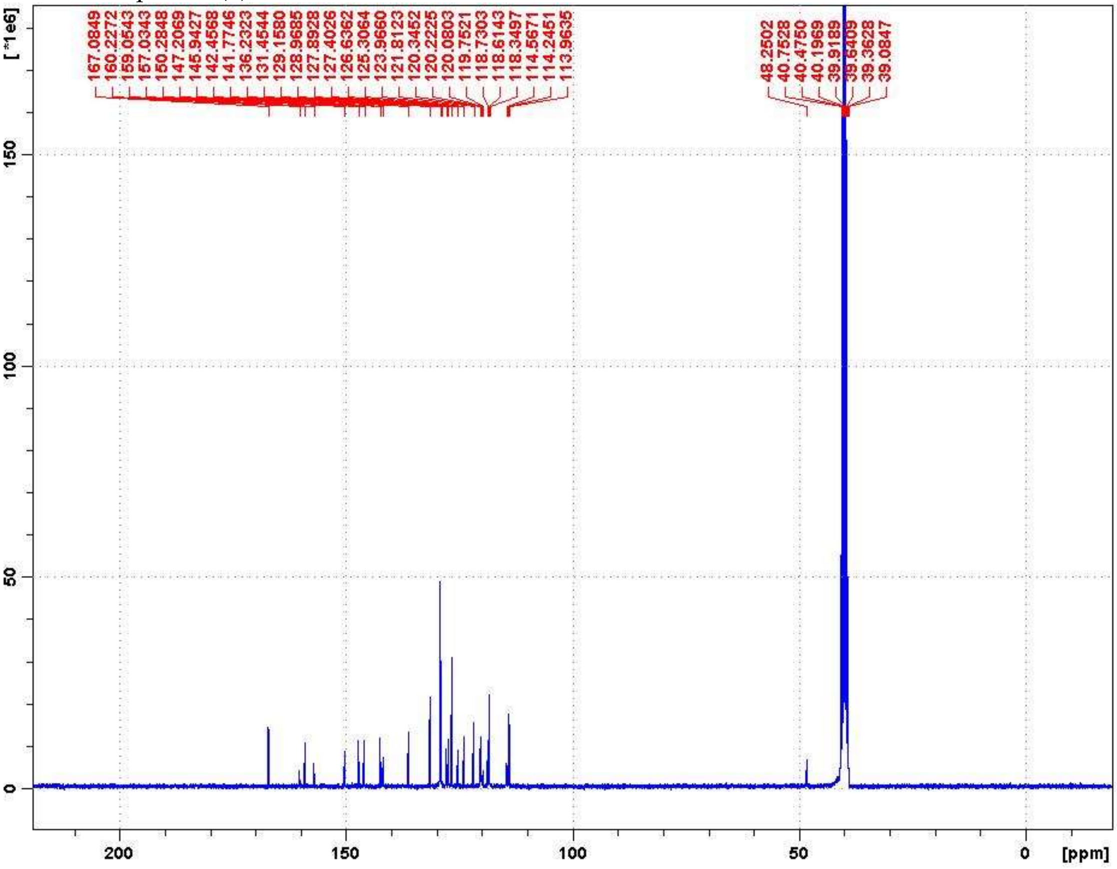

Mass of compound (**8**)

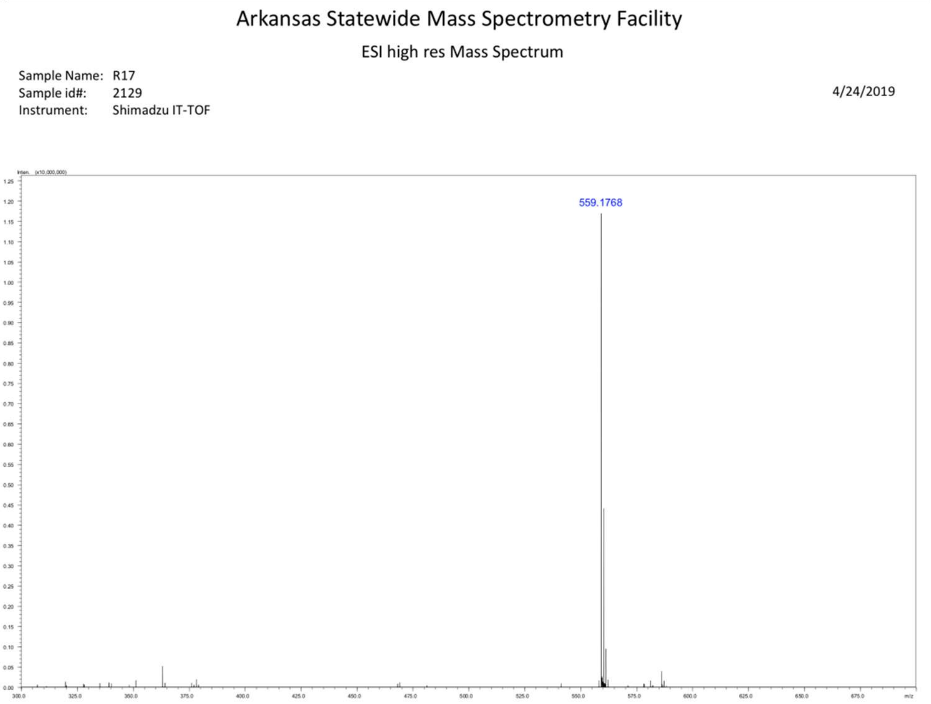

4-[3-(7-fluoro-2-oxo-3,8a-dihydrochromen-3-yl)-4-[(E)-(p-tolylhydrazono)methyl]pyrazol-1-yl]benzoic acid (9). Orange; (481 mg, 89%) ^{1}H NMR (300MHz, DMSO-d$_6$): δ 10.10 (s, 1H), 8.96 (s, 1H), 8.31 (s, 1H), 8.11-8.05 (m, 4H), 7.79-7.72 (m, 2H), 7.60-7.59 (m, 2H), 6.83-6.80 (m, 2H), 6.67-6.65 (m, 2H), 2.14 (s, 3H); ^{13}C NMR (75 MHz, DMSO-d$_6$): δ 167.1, 158.6 (d, ^1J = 239.4 Hz), 159.1, 150.3, 145.9, 143.3, 142.4, 141.9, 131.4, 129.7, 128.9, 128.3, 127.3, 127.1, 123.3, 121.4, 120.3 (d, ^3J = 9.6 Hz), 120.0 (d, ^2J = 24.2 Hz), 118.5 (d, ^3J = 8.4 Hz), 118.3, 114.4 (d, ^2J = 24.0 Hz), 112.0, 20.6. HRMS (ESI-FTMS Mass (m/z): calcd for $C_{27}H_{19}FN_4O_4$ [M+H]$^+$ = 483.1463, found 483.1455.

^{1}H NMR of compound (**9**)

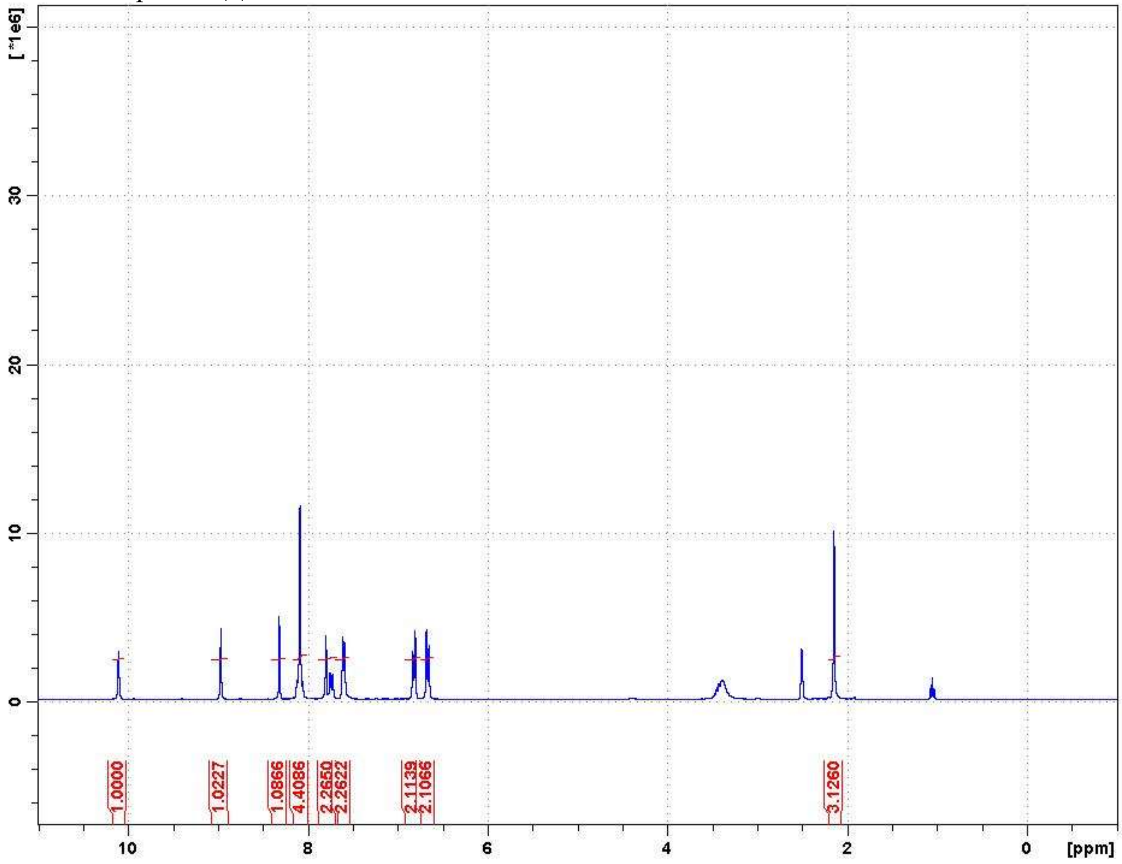

^{13}C NMR of compound (**9**)

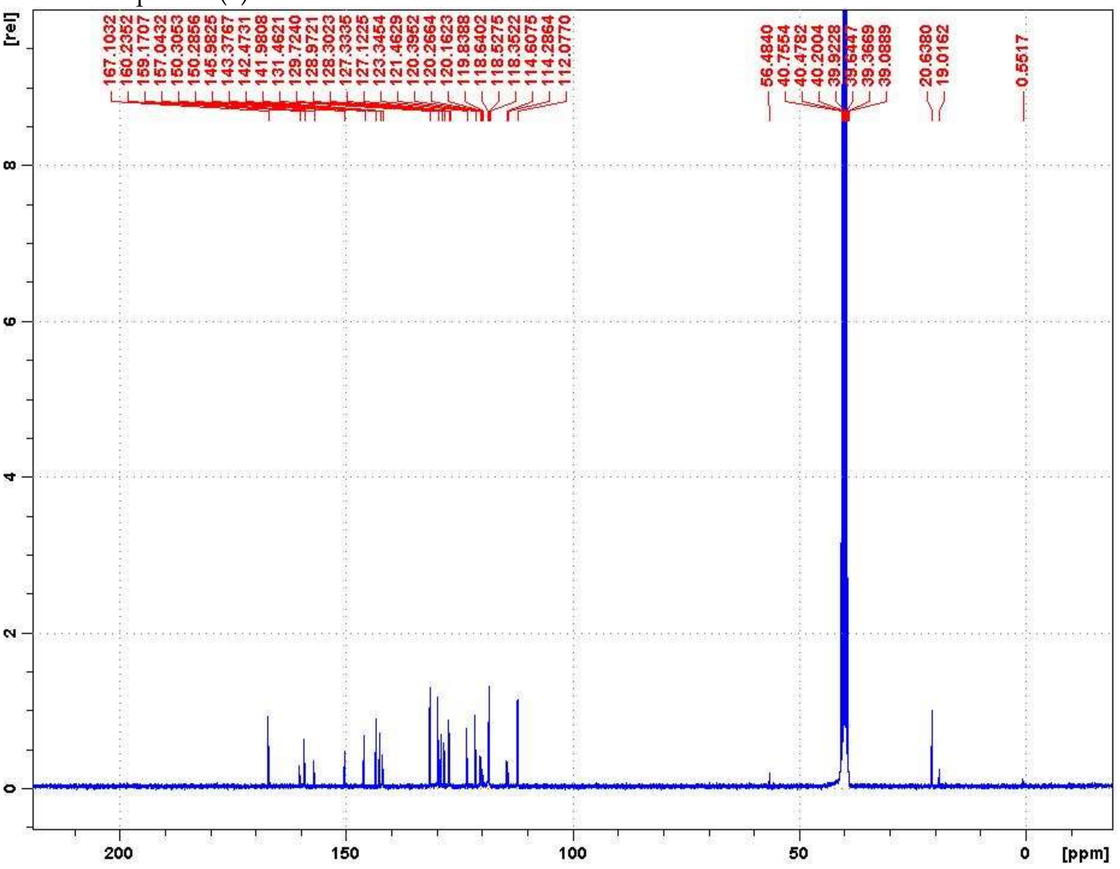

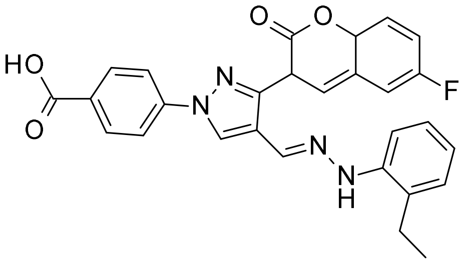

4-[4-[(E)-[(2-ethylphenyl)hydrazono]methyl]-3-(7-fluoro-2-oxo-3,8a-dihydrochromen-3-yl)pyrazol-1-yl]benzoic acid (10). Orange; (393 mg, 79%)^{1}H NMR (300MHz, DMSO-d_6): 9.52 (s, 1H), 9.00 (s, 1H), 8.32 (s, 1H), 8.12-8.10 (m, 5H), 7.75-7,72 (m, 1H), 7.60-7.58 (m, 2H), 6.99-6.91 (m, 2H), 6.70-6.60 (m, 2H), 2.56-2.49 (m, 2H), 1.11 (t, J = 7.3 Hz, 3H); ^{13}C NMR (75 MHz, DMSO-d_6): δ 167.1, 158.6 (d, J = 239.3 Hz), 159.1, 150.2, 146.1, 142.7, 142.4, 142.0, 131.4, 130.2, 129.9, 128.9, 128.6, 127.3, 126.5, 123.4, 121.5, 120.3 (d, ^{3}J = 9.6 Hz), 119.8, 119.0, 118.6 (d, ^{3}J = 8.8 Hz), 118.3, 114.4 (d, ^{2}J = 24.3 Hz), 112.3, 23.5, 14.3. HRMS (ESI-FTMS Mass (m/z): calcd for $C_{28}H_{21}FN_4O_4$ [M+H]$^+$ = 497.1620, found 497.1609.

^{1}H NMR of compound (**10**)

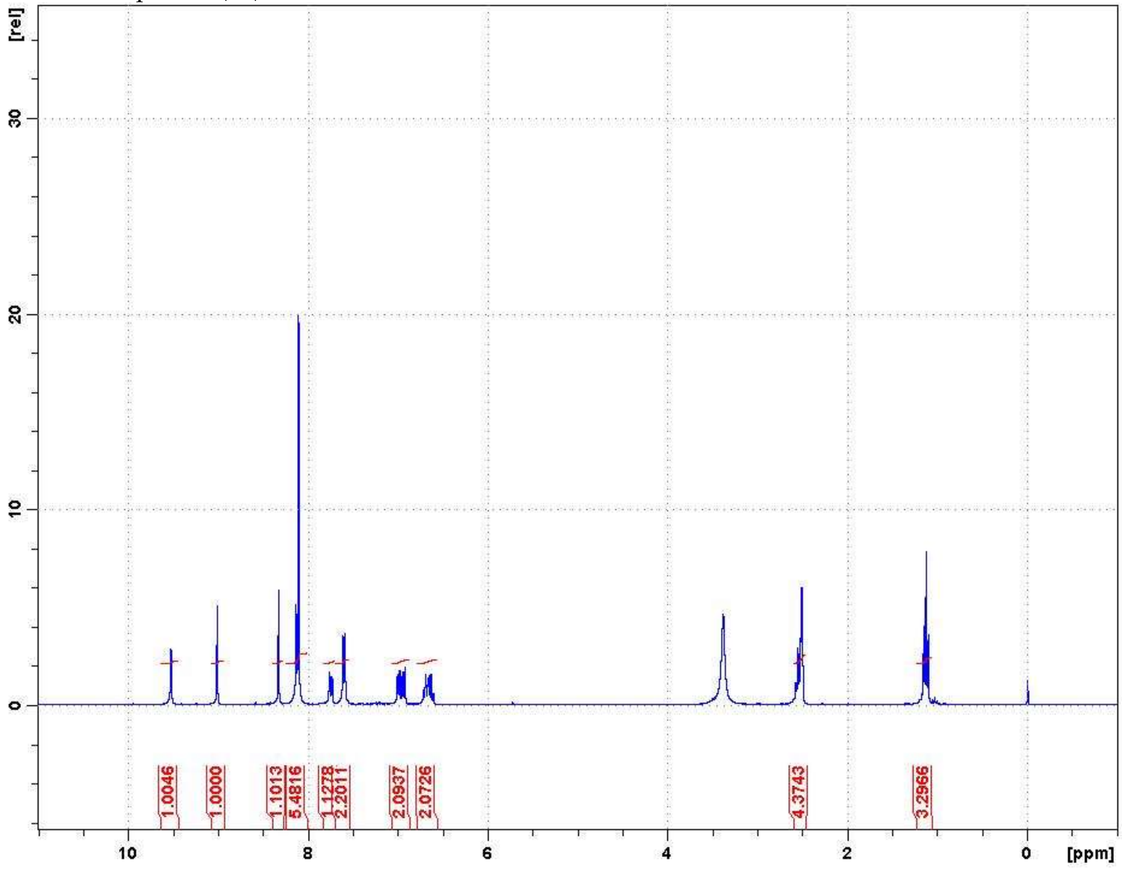

^{13}C NMR of compound (**10**)

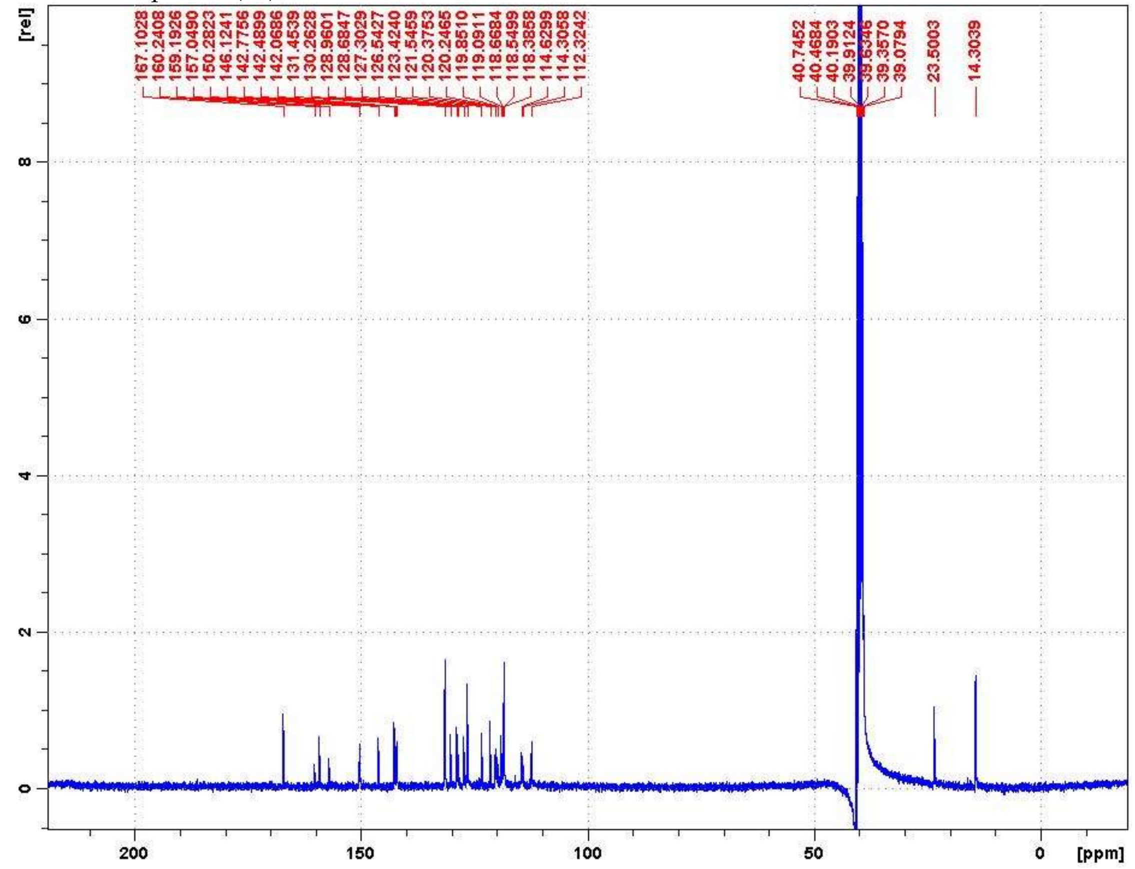

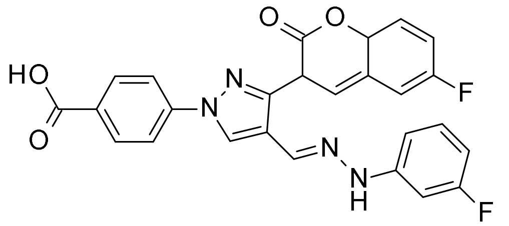

4-[3-(7-fluoro-2-oxo-3,8a-dihydrochromen-3-yl)-4-[(E)-[(3-fluorophenyl)hydrazono]methyl]pyrazol-1-yl]benzoic acid (11). Orange; (395 mg, 81%) ^{1}H NMR (300MHz, DMSO-d$_6$): 10.45 (s, 1H), 9.03 (s, 1H), 8.34 (s, 1H), 8.09-8.06 (m, 4H), 7.86 (s,1H), 7.73 (d, J = 7.1 Hz, 1H), 7.58-7.56 (m, 2H), 7.09-7.01 (m, 1H), 6.56-6.51 (m, 2H), 6.39 (t, J = 6.3 Hz, 1H); ^{13}C NMR (75 MHz, DMSO-d$_6$): 167.0, 163.7 (d, ^{1}J = 239.0 Hz), 158.6 (d, ^{1}J = 239.4 Hz), 159.1, 150.2, 147.6 (d, ^{3}J = 11.0 Hz), 146.0, 142.4, 142.1, 131.4, 130.8 (d, ^{3}J = 9.9 Hz), 130.3, 129.0, 127.7, 123.1, 121.0, 120.2 (d, ^{3}J = 9.7 Hz), 119.8, 118.6 (d, ^{3}J = 8.5 Hz), 118.4, 114.4 (d, ^{2}J = 24.1 Hz), 108.2, 104.8 (d, ^{2}J = 21.3 Hz), 98.5 (d, ^{2}J = 26.1 Hz). HRMS (ESI-FTMS Mass (m/z): calcd for $C_{26}H_{16}F_2N_4O_4$ $[M+H]^+$ = 487.1212, found 487.1198.

^{1}H NMR of compound (**11**)

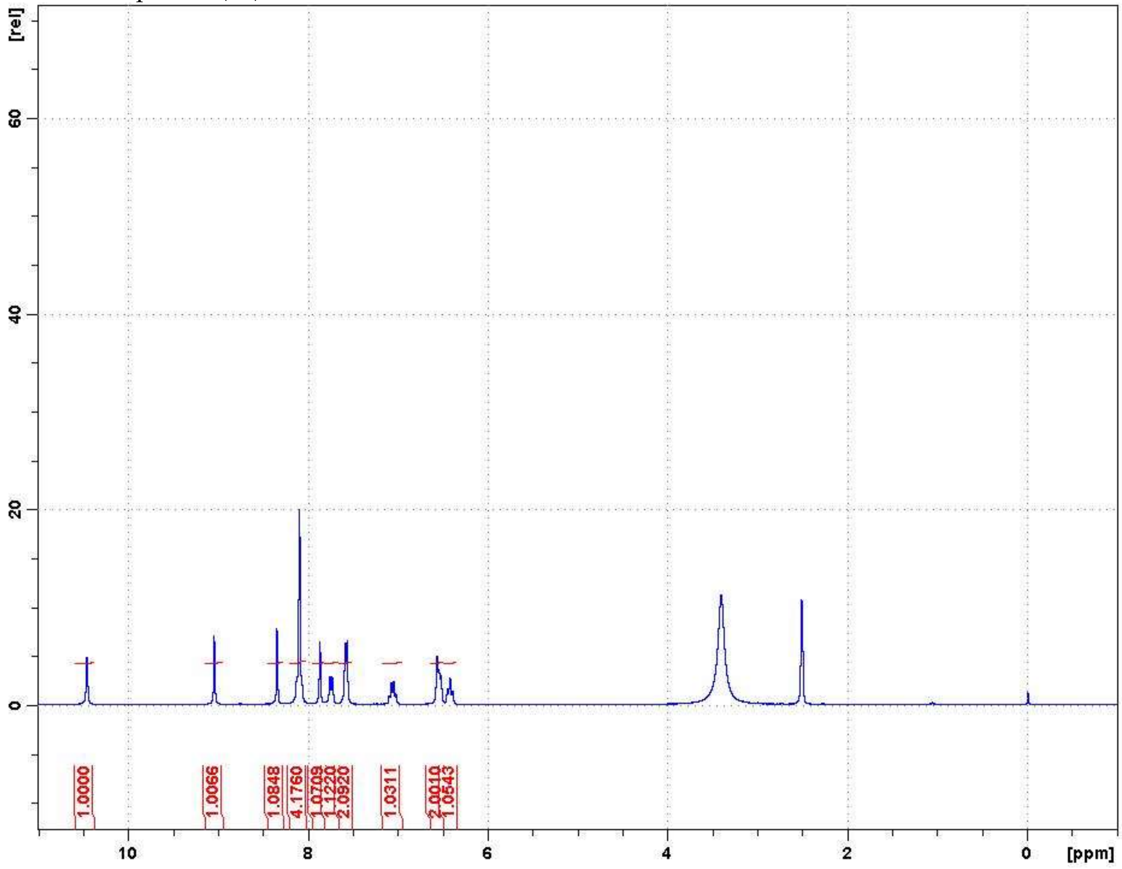

^{13}C NMR of compound (**11**)

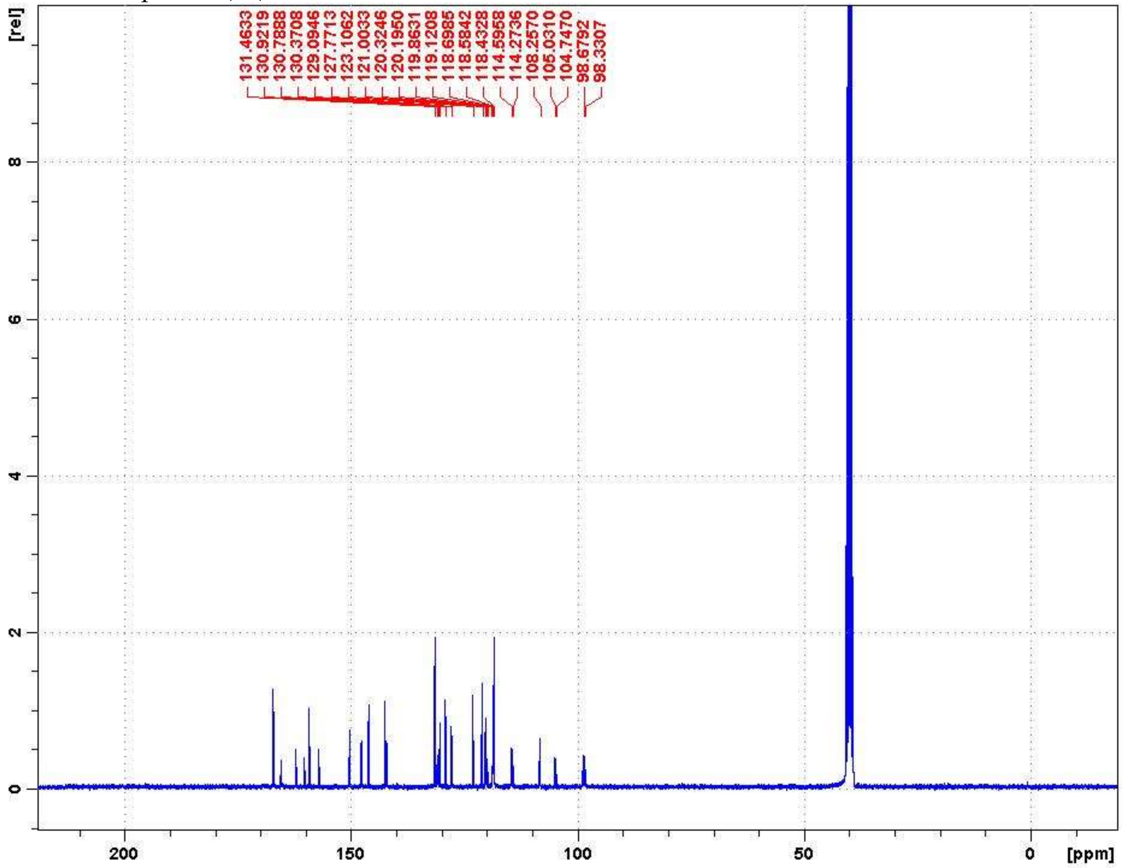

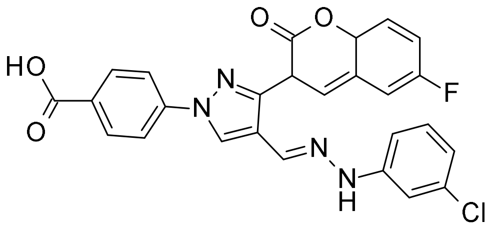

4-[4-[(E)-[(3-chlorophenyl)hydrazono]methyl]-3-(7-fluoro-2-oxo-3,8a-dihydrochromen-3-yl)pyrazol-1-yl]benzoic acid (12). Orange; (429 mg, 85%)^{1}H NMR (300MHz, DMSO-d_6): 10.42 (s, 1H), 9.02 (s, 1H), 8.34 (s, 1H), 8.12-8.05 (m, 4H), 7.88 (s, 1H), 7.74-7.72 (m, 1H), 7.58-7.56 (m, 2H), 7.05 (t, J = 8.0 Hz, 1H), 6.77 (s, 1H), 6.66 (t, J = 8.1 Hz, 2H); ^{13}C NMR (75 MHz, DMSO-d_6): δ 167.0, 158.6 (d, ^{1}J = 239.3 Hz), 159.1, 150.3, 147.0, 146.0, 142.4, 142.1, 134.3, 131.4, 130.7 (d, ^{3}J = 22.5 Hz), 129.0, 127.9, 123.1, 120.9, 120.3, 120.1, 119.8, 118.8 (d, ^{3}J = 8.7 Hz), 118.4, 118.2, 114.4 (d, ^{2}J = 24.3 Hz), 111.1, 110.8. HRMS (ESI-FTMS Mass (m/z): calcd for $C_{26}H_{16}ClFN_4O_4$ $[M+H]^+$ = 503.0917, 505.0890, found 503.0902, 505.0905.

^{1}H NMR of compound (**12**)

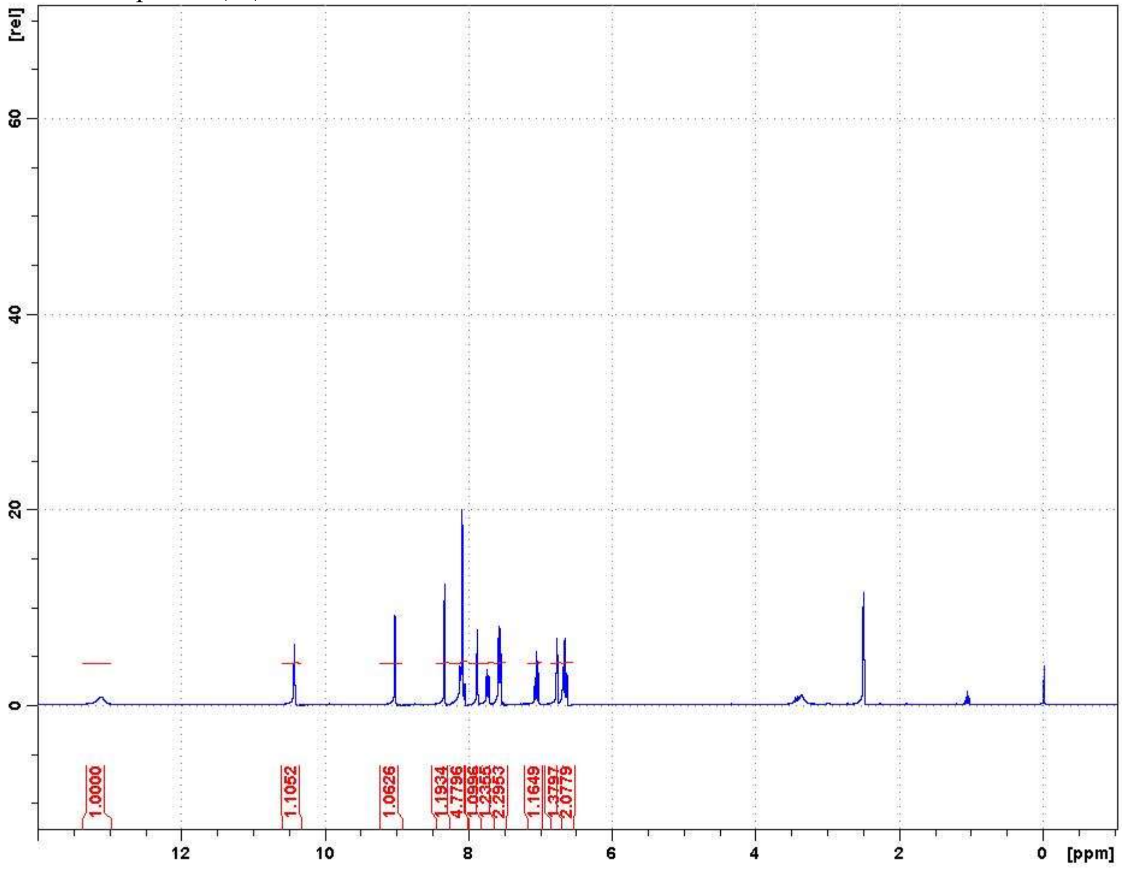

^{13}C NMR of compound (**12**)

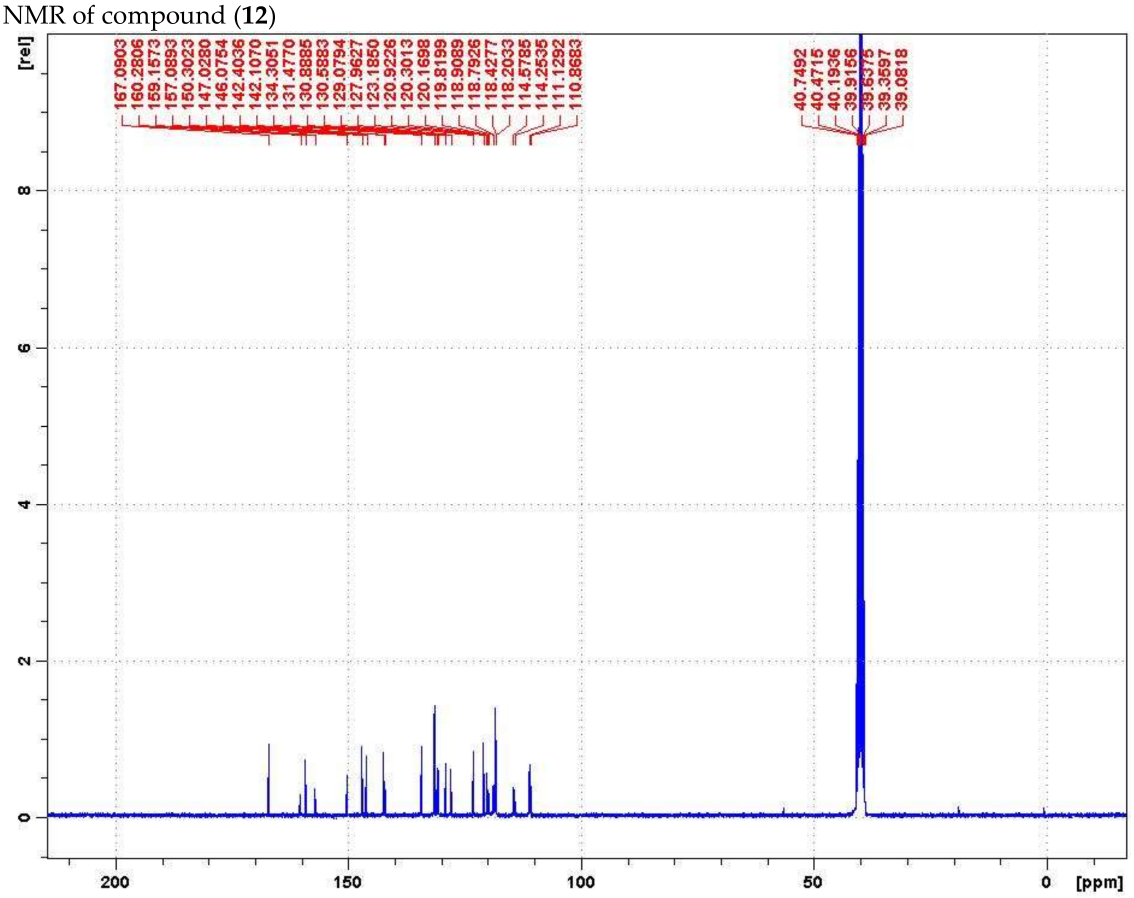

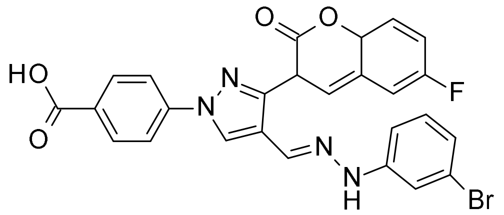

4-[4-[(E)-[(3-bromophenyl)hydrazono]methyl]-3-(7-fluoro-2-oxo-3,8a-dihydrochromen-3-yl)pyrazol-1-yl]benzoic acid (13). Orange; (428 mg, 78%) ^{1}H NMR (300MHz, DMSO-d_6): δ 10.40 (s, 1H), 9.02 (s, 1H), 8.33 (s, 1H), 8.12-8.05 (m, 4H), 7.88 (s, 1H), 7.74-7.71 (m, 1H), 7.58-7.52 (m, 2H), 7.01-7.93 (m, 2H), 6.79-6.70 (m, 2H); ^{13}C NMR (75 MHz, DMSO-d_6): δ 167.0, 158.6 (d, ^{1}J = 239.3 Hz), 159.1, 150.3, 147.1, 146.0, 142.4, 142.1, 131.4, 131.2, 130.6, 129.0, 127.9, 123.1, 122.9, 121.1, 120.8, 120.2 (d, ^{3}J = 9.7 Hz), 119.8, 118.9 (d, ^{3}J = 8.4 Hz), 118.4, 114.4 (d, ^{2}J = 24.1 Hz), 113.9, 111.2. HRMS (ESI-FTMS Mass (m/z): calcd for $C_{26}H_{16}BrFN_4O_4$ $[M+H]^+$ = 547.0412, 549.0393, found 547.0400, 549.0394.

^{1}H NMR of compound (**13**)

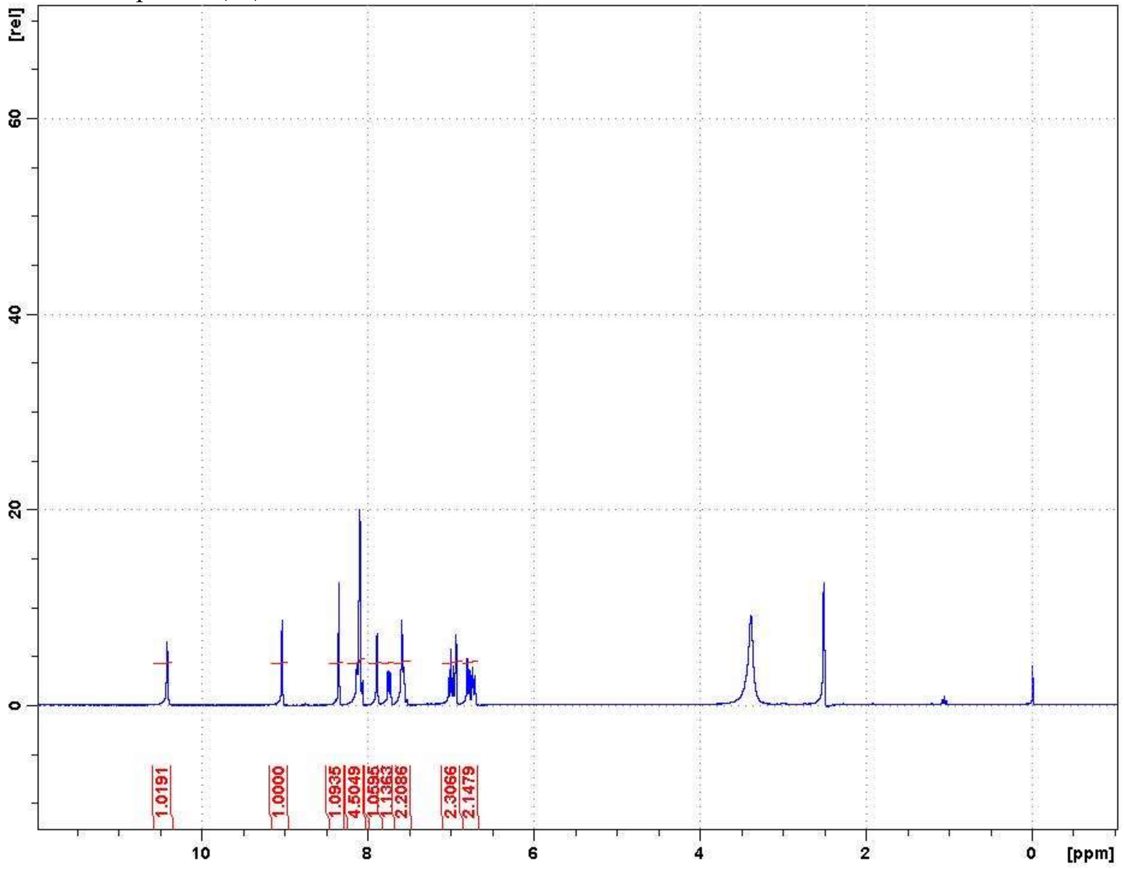

^{13}C NMR of compound (**13**)

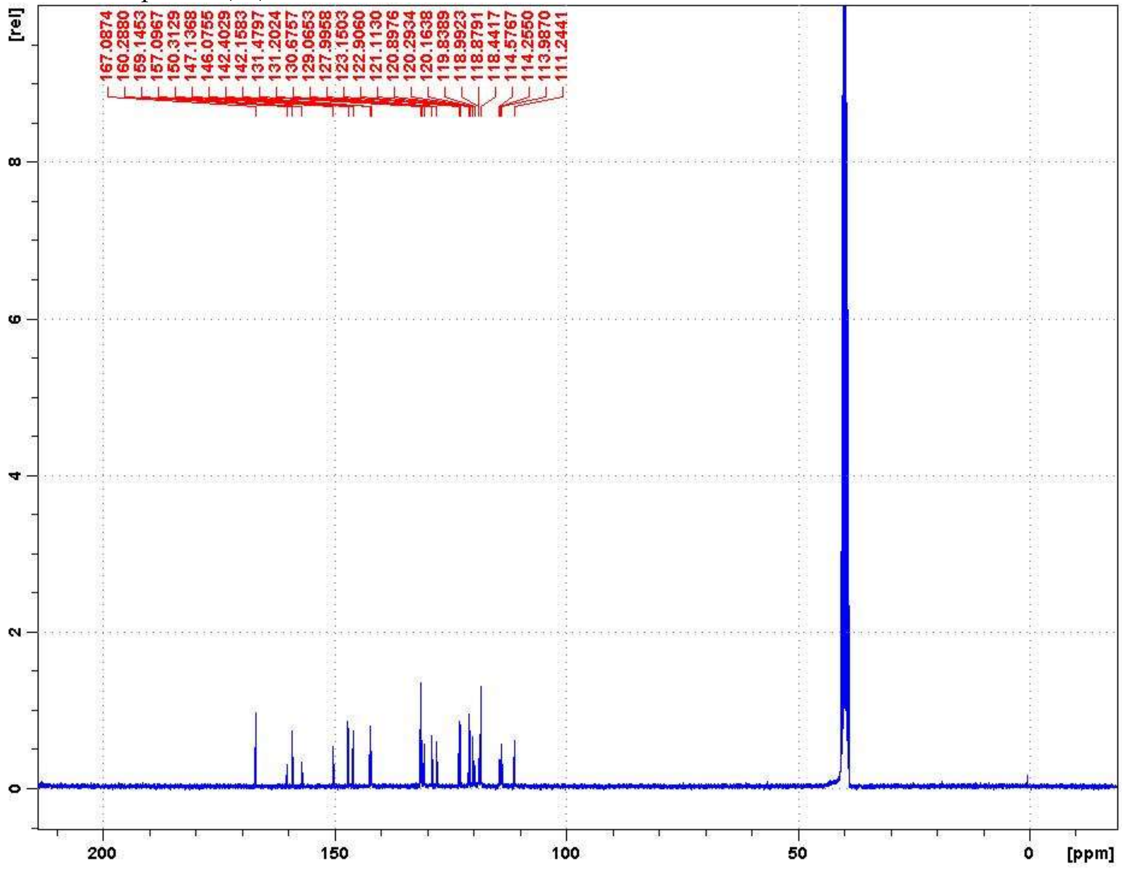

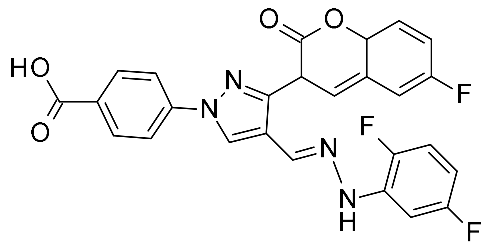

4-[4-[(E)-[(2,5-difluorophenyl)hydrazono]methyl]-3-(6-fluoro-2-oxo-3,8a-dihydrochromen-3-yl)pyrazol-1-yl]benzoic acid (14). Orange; (379 mg, 75%) ^{1}H NMR (300MHz, DMSO-d$_6$): 10.38 (s, 1H), 9.08 (s, 1H), 8.33 (s, 1H), 8.14-8.05 (m, 5H), 7.72 (d, *J* = 7.2 Hz, 1H), 7.60-7.50 (m, 2H), 7.13-7.05 (m, 1H), 6.75-6.68 (m, 1H), 6.45-6.38 (m, 1H); ^{13}C NMR (75 MHz, DMSO-d$_6$): δ 167.1, 159.5 (d, ^1J = 236.5 Hz), 158.6 (d, ^1J = 239.5 Hz), 159.1, 150.2, 145.4 (d, ^1J = 233.5 Hz), 146.1, 142.3, 142.2, 135.1 (t, ^3J = 11.8 Hz), 133.1, 131.4, 129.2, 128.2, 122.9, 120.7, 120.2, 120.0 (d, ^3J = 13.1 Hz), 118.6 (d, ^3J = 8.6 Hz), 118.4, 116.4-116.0 (m), 114.4 (d, ^2J = 24.2 Hz), 103.8 (d, ^3J = 17.7 Hz), 100.2 (d, ^2J = 30.0 Hz). HRMS (ESI-FTMS Mass (m/z): calcd for $C_{26}H_{15}F_3N_4O_4$ $[M+H]^+$ = 505.1118, found 505.1110.

^{1}H NMR of compound (**14**)

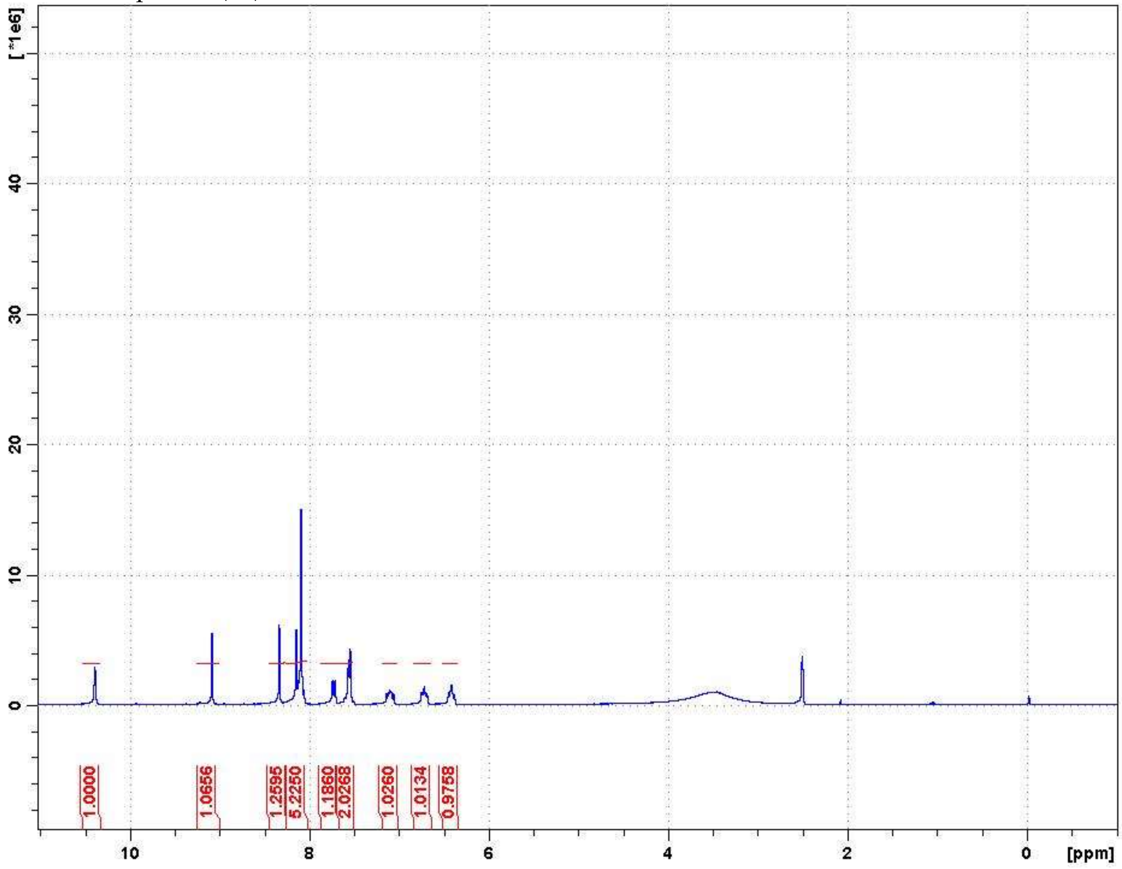

^{13}C NMR of compound (**14**)

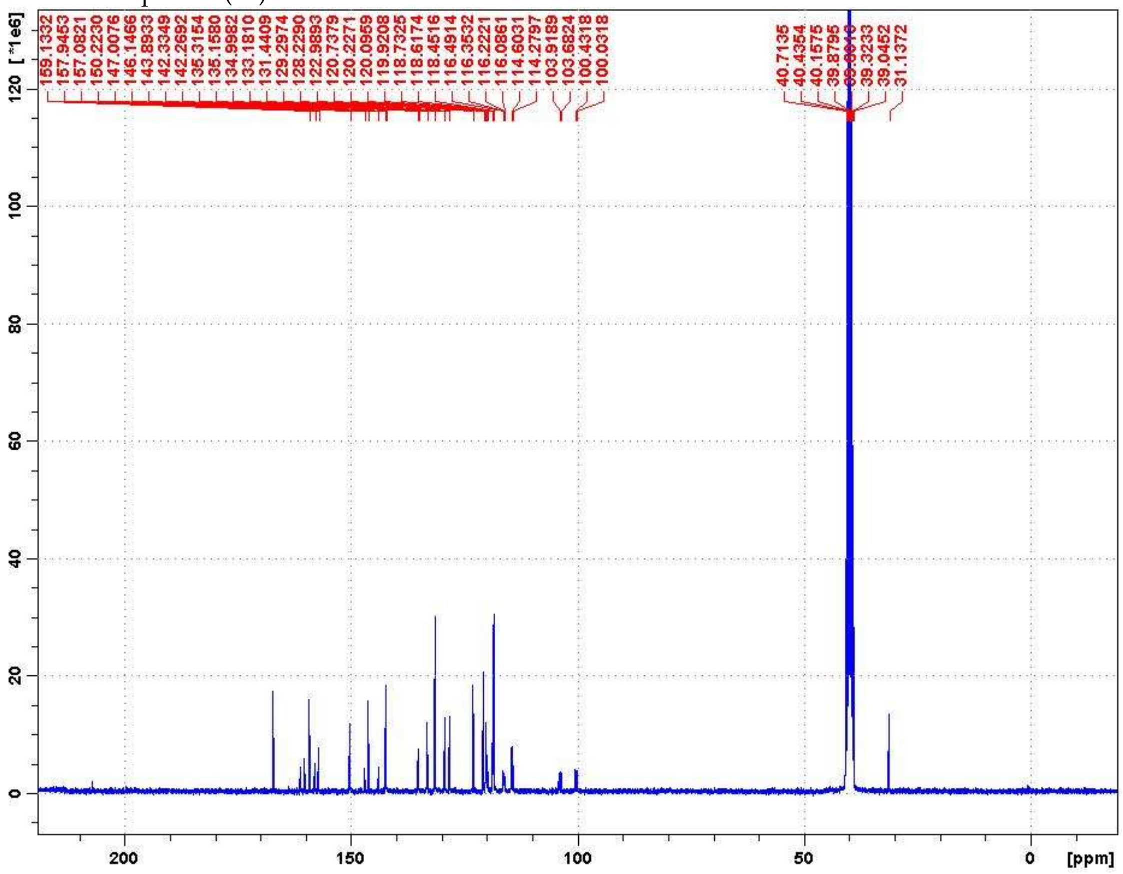

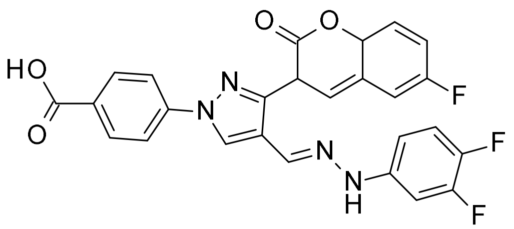

4-[4-[(E)-[(3,4-difluorophenyl)hydrazono]methyl]-3-(6-fluoro-2-oxo-3,8a-dihydrochromen-3-yl)pyrazol-1-yl]benzoic acid (15). Orange; (425 mg, 84%) ^{1}H NMR (300MHz, DMSO-d$_6$): 10.40 (s, 1H), 9.02 (s, 1H), 8.33 (s, 1H), 8.11-8.04 (m, 4H), 7.84 (s, 1H), 7.75-7.72 (m, 1H), 7.57 (d, *J* = 5.8 Hz, 2H), 7.16-7.07 (m, 1H), 6.71-6.65 (m, 1H), 6.52-6.49 (m, 1H); ^{13}C NMR (75 MHz, DMSO-d$_6$): δ 167.0, 158.6 (d, ^{1}J = 239.4 Hz), 159.2, 150.4 (dd, *J* = 13.0, 240.6 Hz), 150.6, 146.0, 142.8 (dd, *J* = 12.8, 233.6 Hz), 143.2 (d, ^{3}J = 8.4 Hz), 142.4, 142.1, 131.4, 130.4, 129.0, 127.7, 122.9, 120.9, 120.2 (d, ^{3}J = 9.5 Hz), 120.25, 119.9, 118.6 (d, ^{3}J = 8.5 Hz), 118.4, 118.1, 117.9, 114.4 (d, ^{2}J = 24.1 Hz). HRMS (ESI-FTMS Mass (m/z): calcd for $C_{26}H_{15}F_3N_4O_4$ [M+H]$^+$ = 505.1118, found 505.1106.

^{1}H NMR of compound (**15**)

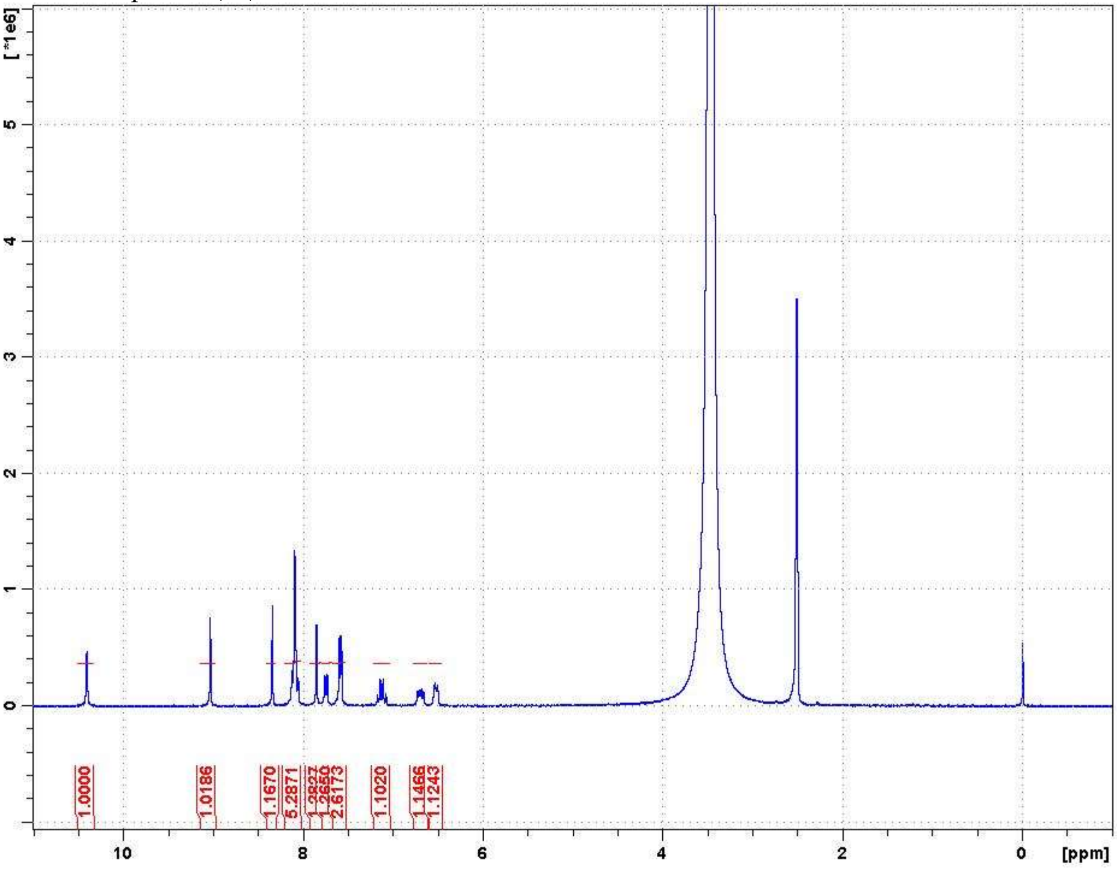

^{13}C NMR of compound (**15**)

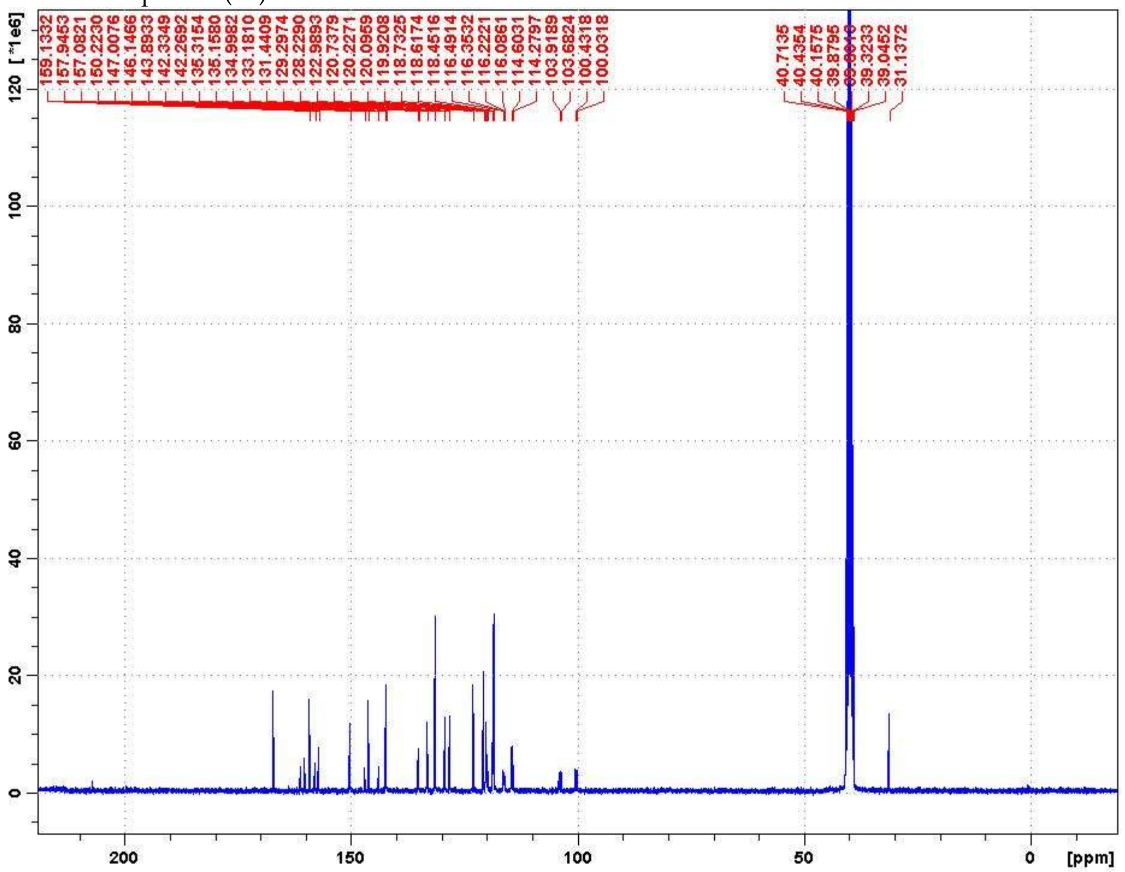

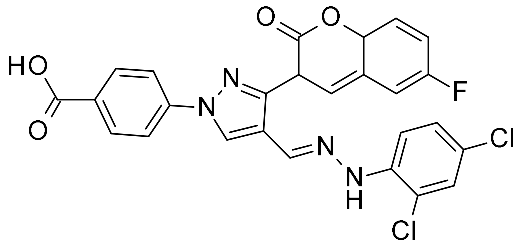

4-[4-[(E)-[(2,4-dichlorophenyl)hydrazono]methyl]-3-(6-fluoro-2-oxo-3,8a-dihydrochromen-3-yl)pyrazol-1-yl]benzoic acid (16). Yellowish; (415 mg, 77%)^{1}H NMR (300MHz, DMSO-d_6): 13.11 (br s, 1H), 9.98 (s, 1H), 9.08 (s, 1H), 8.34 (s, 1H), 8.21 (s, 1H), 8.10 (s, 4H), 7.76-7.72 (m, 1H), 7.62-7.59 (m, 2H), 7.418-7.410 (m, 1H), 7.12-7.09 (s, 1H), 6.96-6.92 (m, 1H); ^{13}C NMR (75 MHz, DMSO-d_6): δ 167.0, 158.6 (d, ^{1}J = 239.7 Hz), 159.1, 150.2, 146.3, 142.3, 140.9, 133.8, 131.4, 129.1, 129.0, 127.9, 127.8, 122.7, 122.2, 120.8, 120.21 (d, ^{2}J = 24.6 Hz), 120.22 (d, ^{3}J = 9.7 Hz) 118.7, 118.54, 118.53, 116.8, 114.8, 114.5 (d, ^{2}J = 24.1 Hz). HRMS (ESI-FTMS Mass (m/z): calcd for $C_{26}H_{15}Cl_2FN_4O_4$ $[M+H]^+$ = 537.0527, 539.0499, found 537.0512, 539.0482.

^{1}H NMR of compound (**16**)

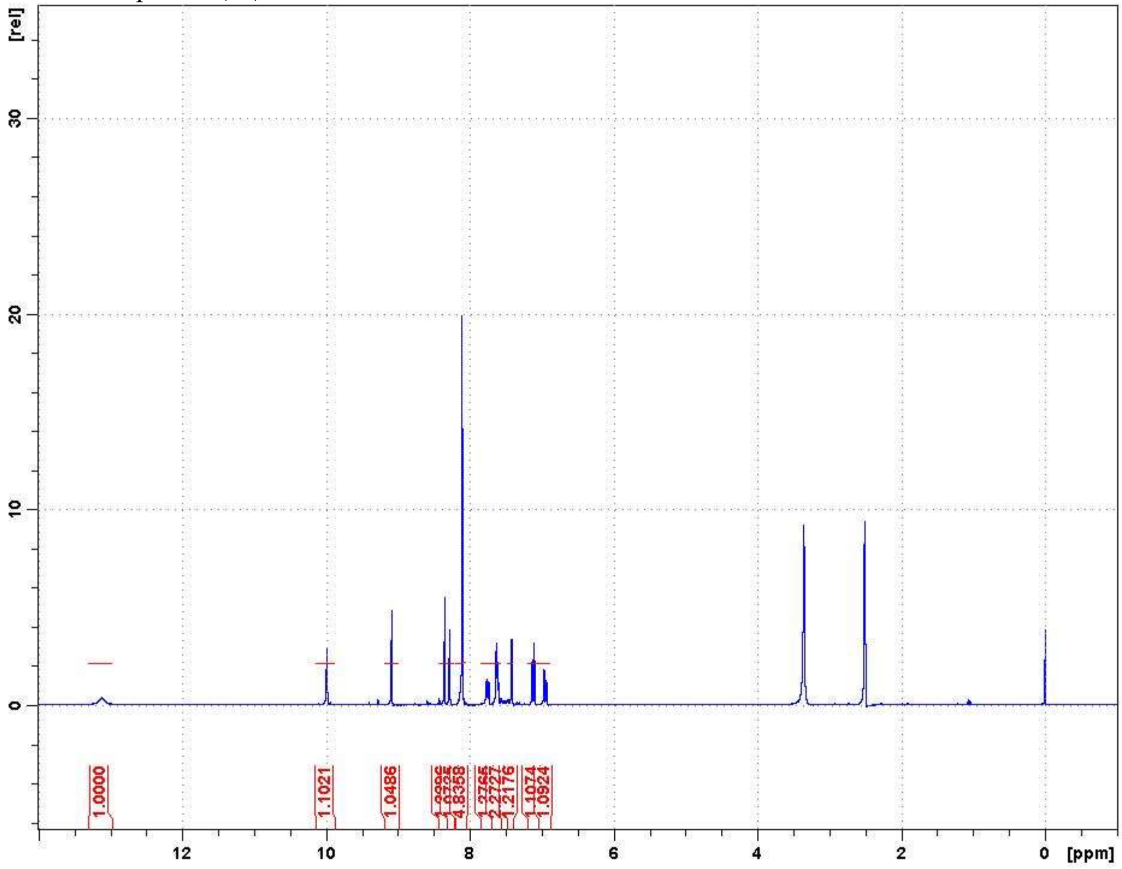

^{13}C NMR of compound (**16**)

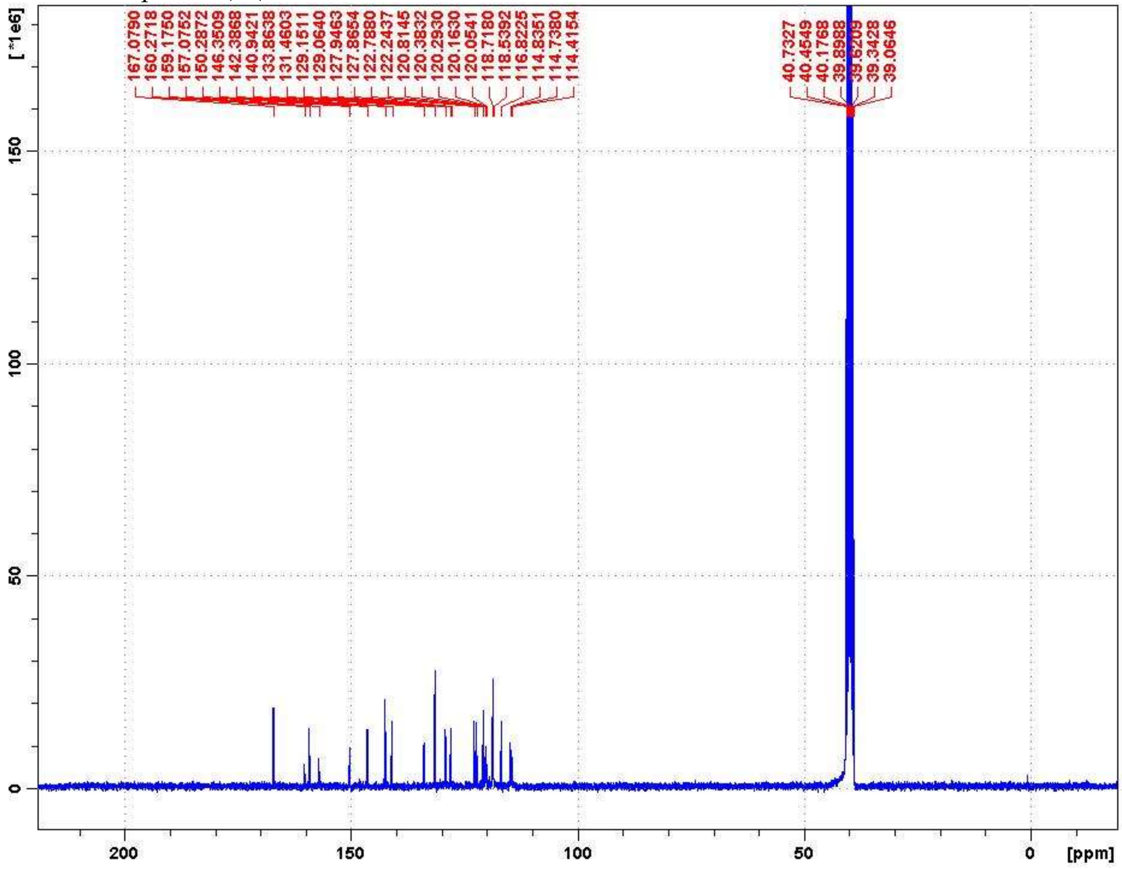

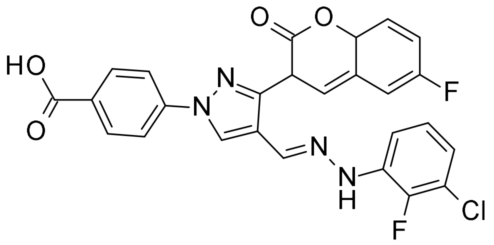

4-[4-[(E)-[(3-chloro-2-fluoro-phenyl)hydrazono]methyl]-3-(6-fluoro-2-oxo-3,8a-dihydrochromen-3-yl)pyrazol-1-yl]benzoic acid (17). Orange; (418 mg, 80%) ^{1}H NMR (300MHz, DMSO-d$_6$): 10.39 (s, 1H), 9.06 (s, 1H), 8.33 (s, 1H), 8.14-8.08 (m, 5H), 7.75-7.72 (m, 1H), 7.60-7.58 (m, 2H), 6.95 (t, J = 7.6 Hz, 1H), 6.81-6.70 (m, 2H); ^{13}C NMR (75 MHz, DMSO-d$_6$): δ 167.0, 158.6 (d, ^{1}J = 239.7 Hz), 159.1, 150.2, 144.6 (d, ^{1}J = 240 Hz), 146.2, 142.3, 142.2, 135.2 (d, ^{3}J = 9.3 Hz), 133.1, 131.4, 129.1, 128.0, 125.4, 125.3, 123.0, 120.7, 120.3, 120.1 (d, ^{3}J = 8.5 Hz), 119.9 (d, ^{3}J = 7.3 Hz), 118.7 (d, ^{3}J = 13.7 Hz), 118.5, 114.5 (d, ^{2}J = 24.4 Hz), 112.5. HRMS (ESI-FTMS Mass (m/z): calcd for $C_{26}H_{15}ClF_2N_4O_4$ $[M+H]^+$ = 521.0823, 523.0796, found 521.0806, 523.0783.

^{1}H NMR of compound (**17**)

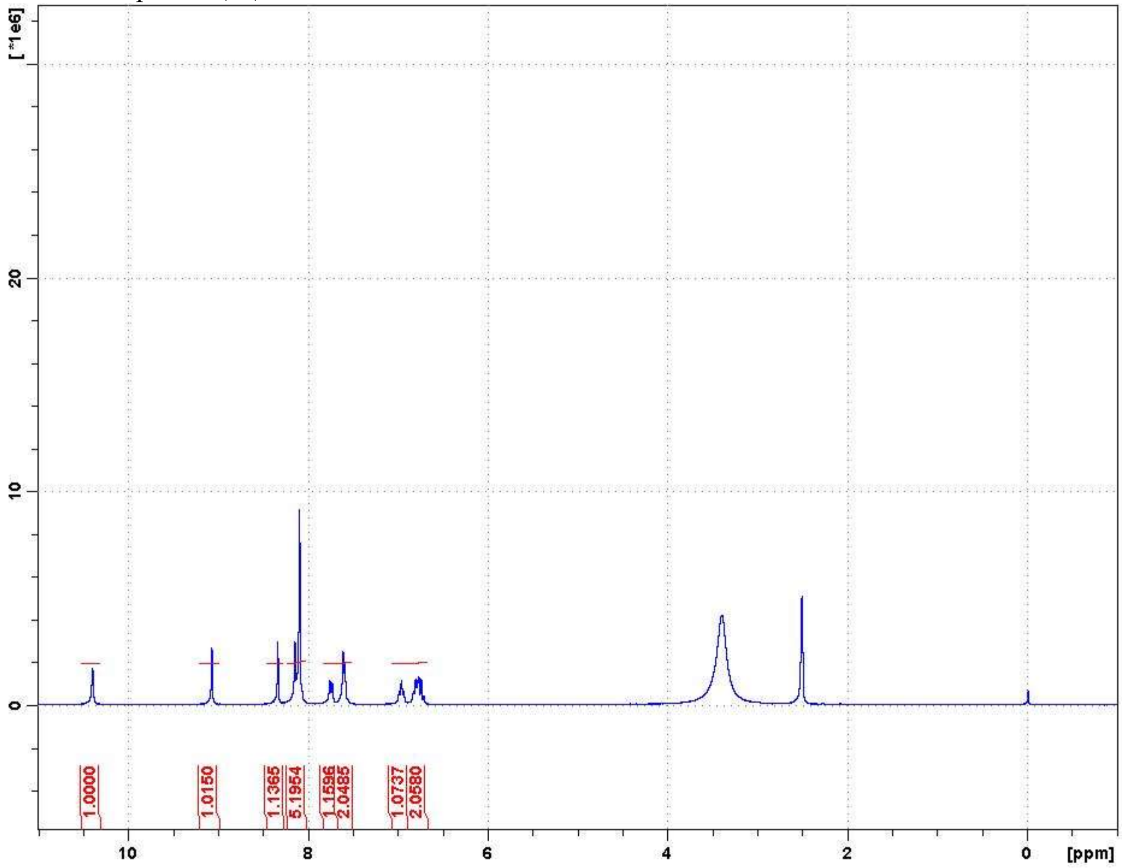

^{13}C NMR of compound (**17**)

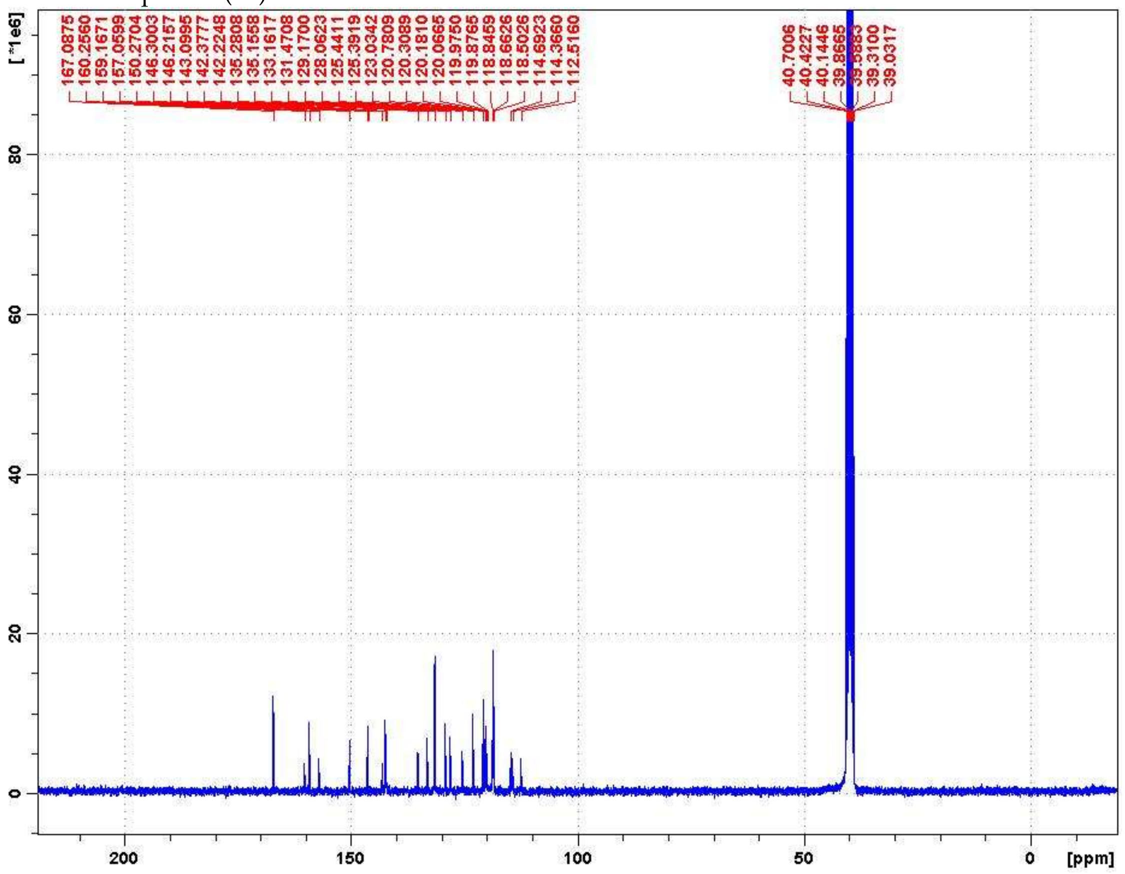

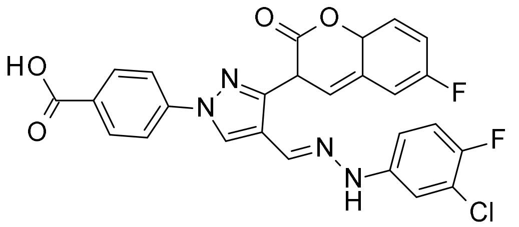

4-[4-[(E)-[(3-chloro-4-fluoro-phenyl)hydrazono]methyl]-3-(6-fluoro-2-oxo-3,8a-dihydrochromen-3-yl)pyrazol-1-yl]benzoic acid (18). Orange; (496 mg, 95%)^{1}H NMR (300MHz, DMSO-d_6): 10.36 (s, 1H), 8.99 (s, 1H), 8.32 (s, 1H), 8.11-8.03 (m, 4H), 7.85 (s, 1H), 7.74-7.71 (m, 1H), 7.58-7.56 (m, 2H), 7.10 (t, J = 9.0 Hz, 1H), 6.83-6.80 (m, 1H), 6.70-6.65 (m, 1H); ^{13}C NMR (75 MHz, DMSO-d_6): δ 167.1, 158.7 (d, ^{1}J = 239.3 Hz), 159.1, 150.8 (d, ^{1}J = 235.0 Hz), 150.2, 146.0, 143.0, 142.3, 142.1, 131.1, 130.5, 129.0, 127.9, 123.1, 120.8, 120.3 (d, ^{3}J = 8.6 Hz), 120.0 (d, ^{2}J = 24.4 Hz), 120.12, 118.8 (d, ^{3}J = 8.6 Hz), 118.4, 117.5 (d, ^{2}J = 21.9 Hz), 114.4 (d, ^{2}J = 24.1 Hz), 112.2, 111.9 (d, ^{3}J = 6.0 Hz). HRMS (ESI-FTMS Mass (m/z): calcd for $C_{26}H_{15}ClF_2N_4O_4$ $[M+H]^+$ = 521.0823, 523.0796, found 521.0818, 523.0793.

^{1}H NMR of compound (**18**)

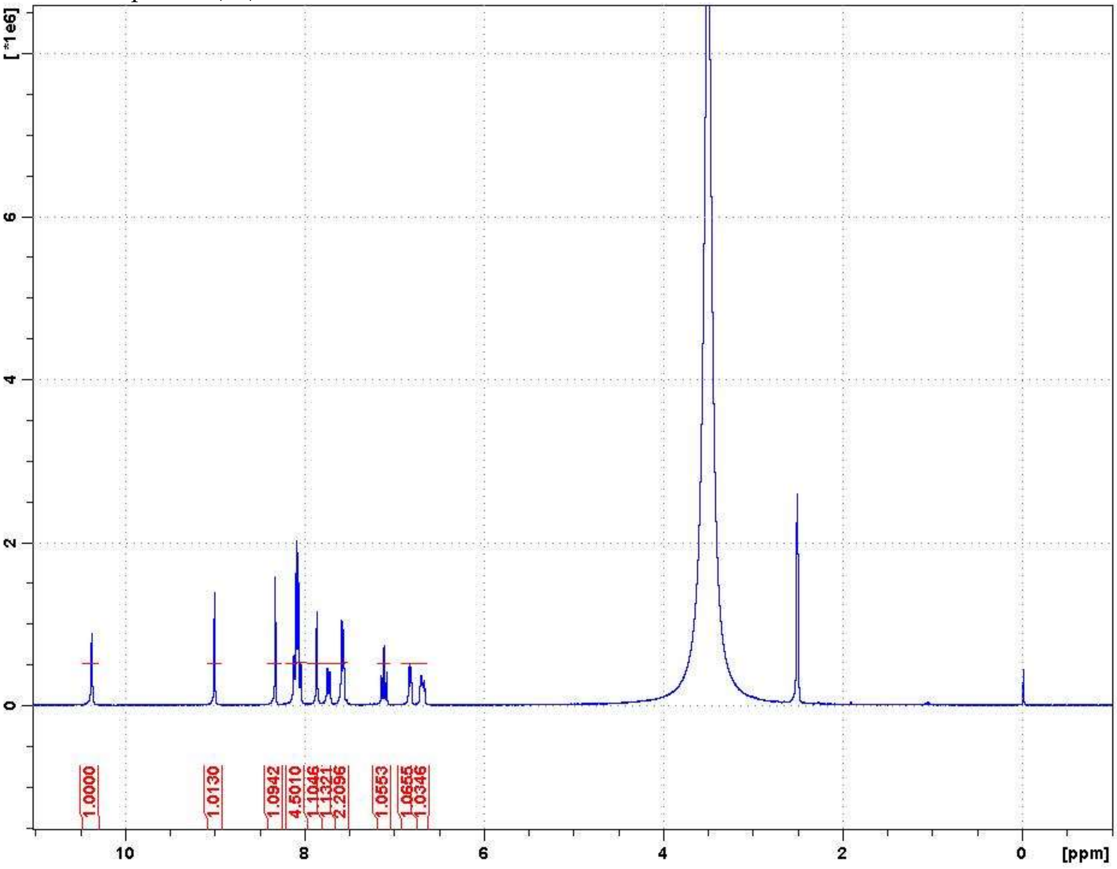

^{13}C NMR of compound (**18**)

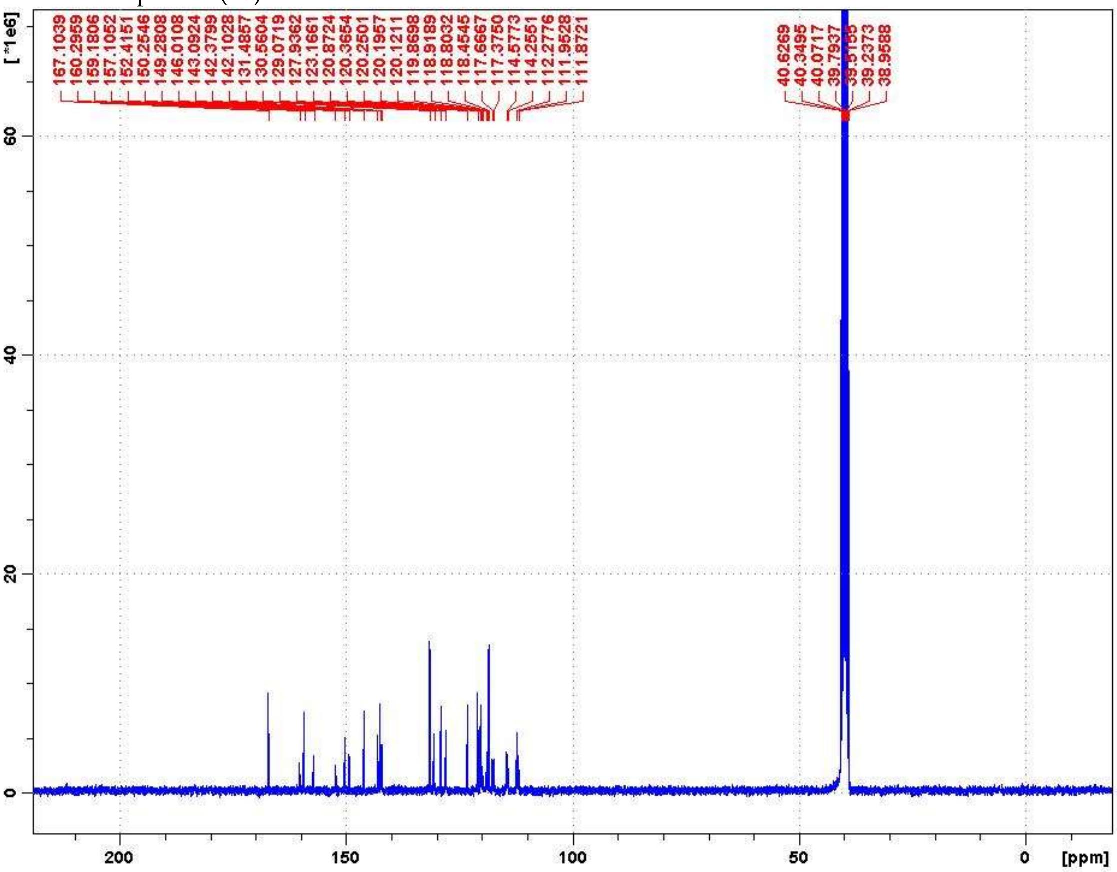

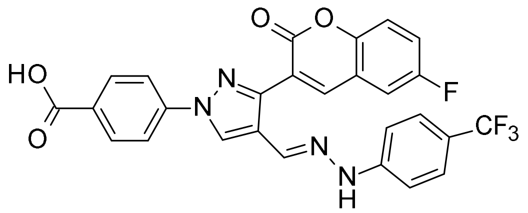

4-[3-(7-fluoro-2-oxo-3,8a-dihydrochromen-3-yl)-4-[(E)-[[4-(trifluoromethyl)phenyl]hydrazono]methyl]pyrazol-1-yl]benzoic acid (19). Yellowish; (393 mg, 73%) ^{1}H NMR (300MHz, DMSO-d_6): 10.70 (s, 1H), 9.04 (s, 1H), 8.34 (s, 1H), 8.12-8.09 (m, 4H), 7.92 (s, 1H), 7.75-7.73 (m, 1H), 7.62-7.59 (m, 2H), 7.35-7.32 (m, 2H), 6.92-6.89 (m, 2H); ^{13}C NMR (75 MHz, DMSO-d_6): δ 167.0, 158.6 (d, ^{1}J = 239.6 Hz), 159.2, 150.2, 148.5, 146.2, 142.3, 142.2, 131.8, 131.4, 129.2, 127.9, 126.6, 125.7 (q, J = 268.6 Hz) 122.9, 120.7, 120.2 (d, ^{3}J = 9.6 Hz), 118.6 (d, ^{3}J = 10.8 Hz), 118.3, 117.8, 114.5 (d, ^{2}J = 24.0 Hz), 111.7. HRMS (ESI-FTMS Mass (m/z): calcd for $C_{27}H_{16}F_4N_4O_4$ $[M+H]^+$ = 537.1180, found 537.1177.

^{1}H NMR of compound (**19**)

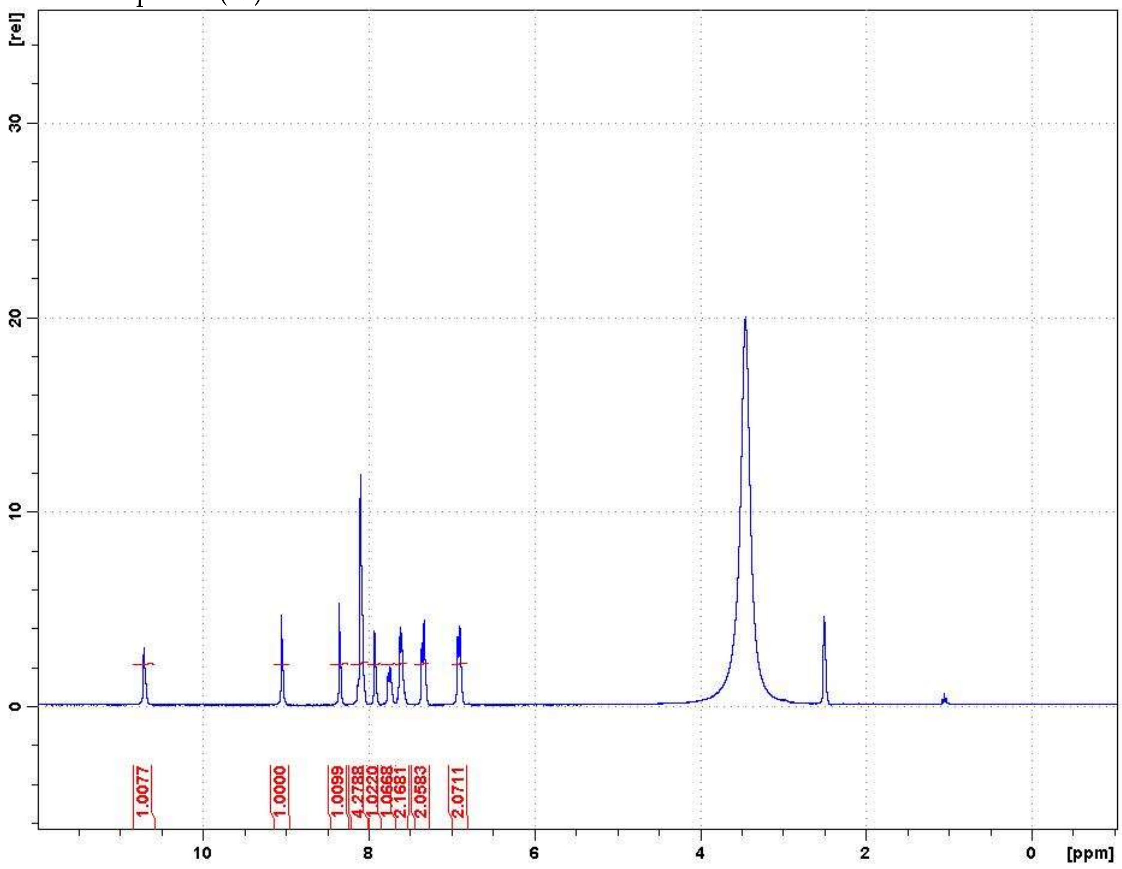

^{13}C NMR of compound (**19**)

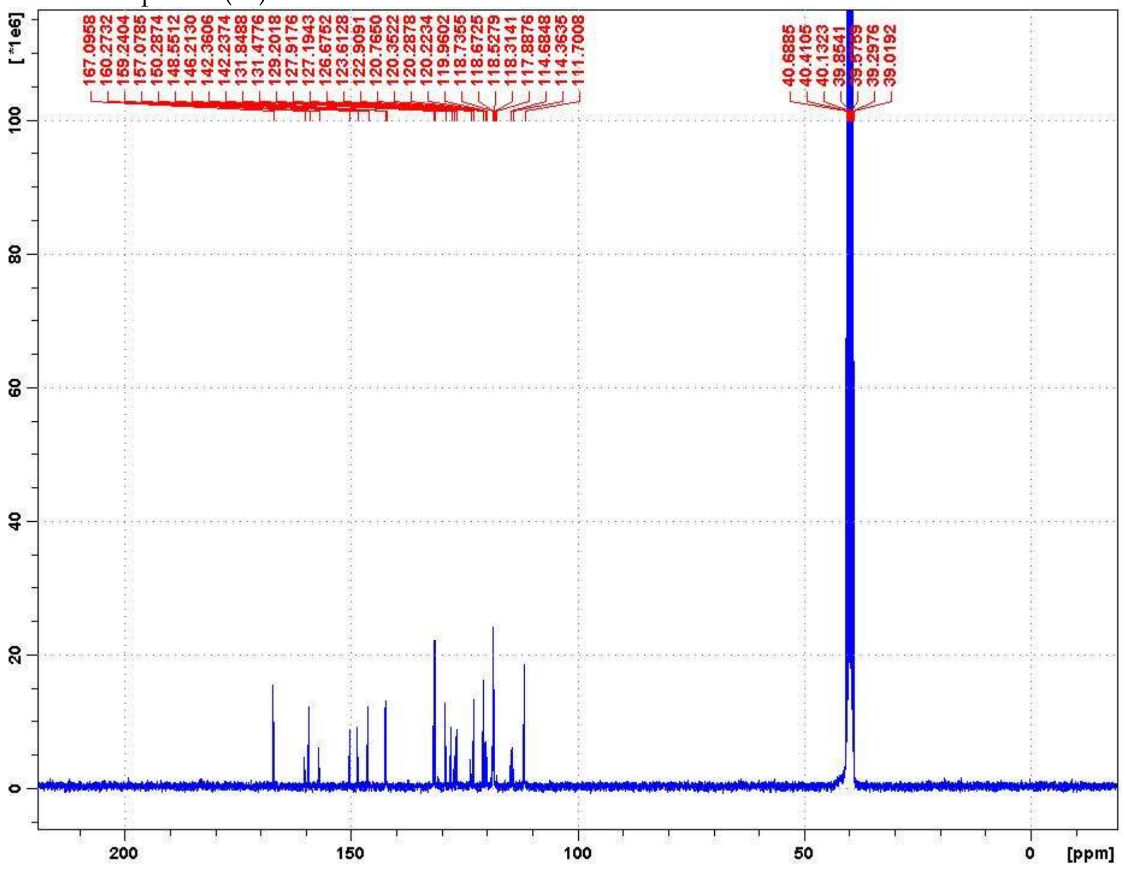

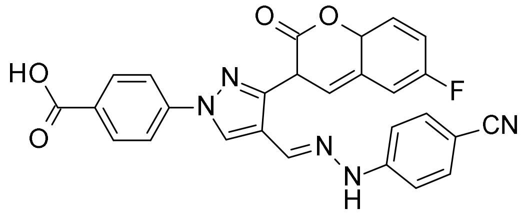

4-[4-[(E)-[(4-cyanophenyl)hydrazono]methyl]-3-(7-fluoro-2-oxo-3,8a-dihydrochromen-3-yl)pyrazol-1-yl]benzoic acid (20). Yellow; (436 mg, 88%) ^{1}H NMR (300MHz, DMSO-d$_6$): δ 10.86 (s, 1H), 9.08 (s, 1H), 8.36 (s, 1H), 8.13-8.06 (m, 4H), 7.95 (s, 1H), 7.76-7.73 (m, 1H), 7.65-7.55 (m, 2H), 7.46-7.43 (m, 2H), 6.90-6.87 (m, 2H); ^{13}C NMR (75 MHz, DMSO-d$_6$): δ 167.1, 159.2, 158.6 (d, ^{1}J = 239.7 Hz), 150.2, 148.8, 146.2, 142.3, 133.8, 133.0, 131.4, 129.3, 128.1, 122.7, 120.54, 120.57, 120.2 (d, ^{3}J = 9.8 Hz), 120.0, 118.7, 118.59, 118.55, 114.5 (d, ^{2}J = 24.0 Hz), 112.1, 99.3. HRMS (ESI-FTMS Mass (m/z): calcd for $C_{27}H_{16}FN_5O_4$ [M+H]$^+$ = 494.1259, found 494.1252.

^{1}H NMR of compound (**20**)

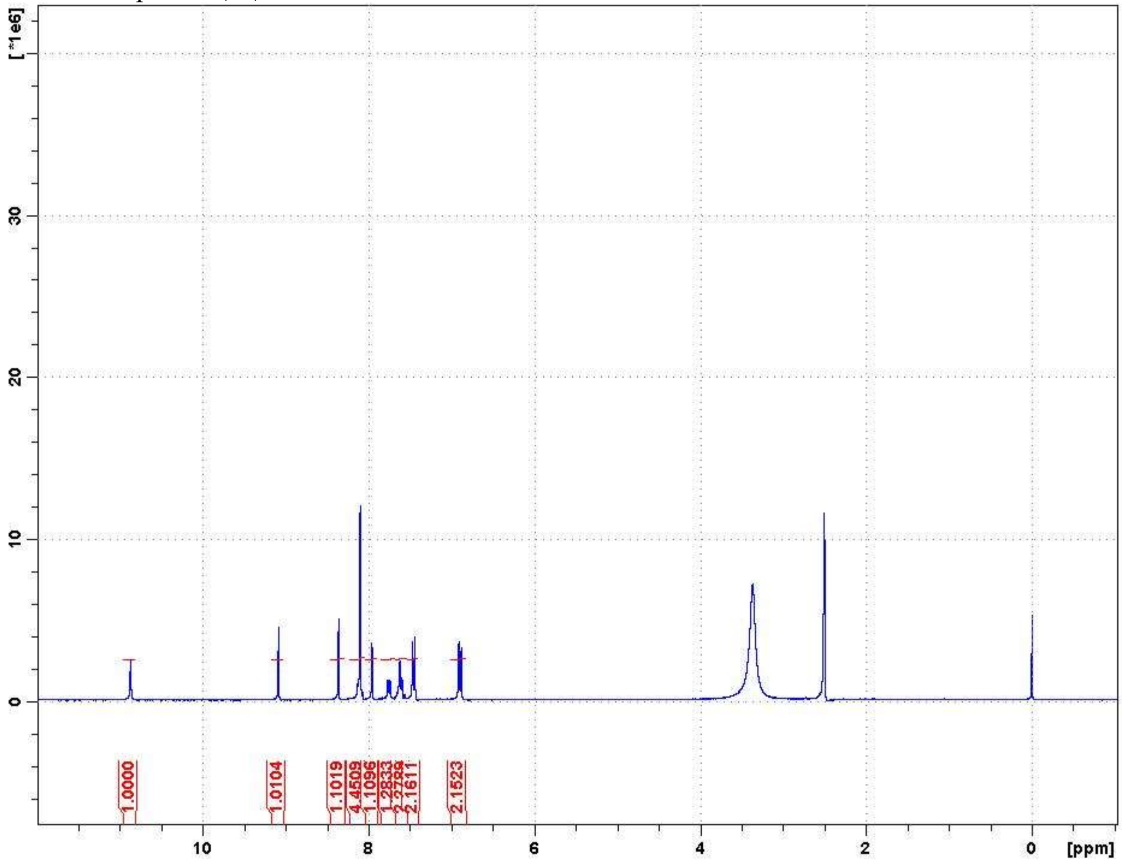

^{13}C NMR of compound (**20**)

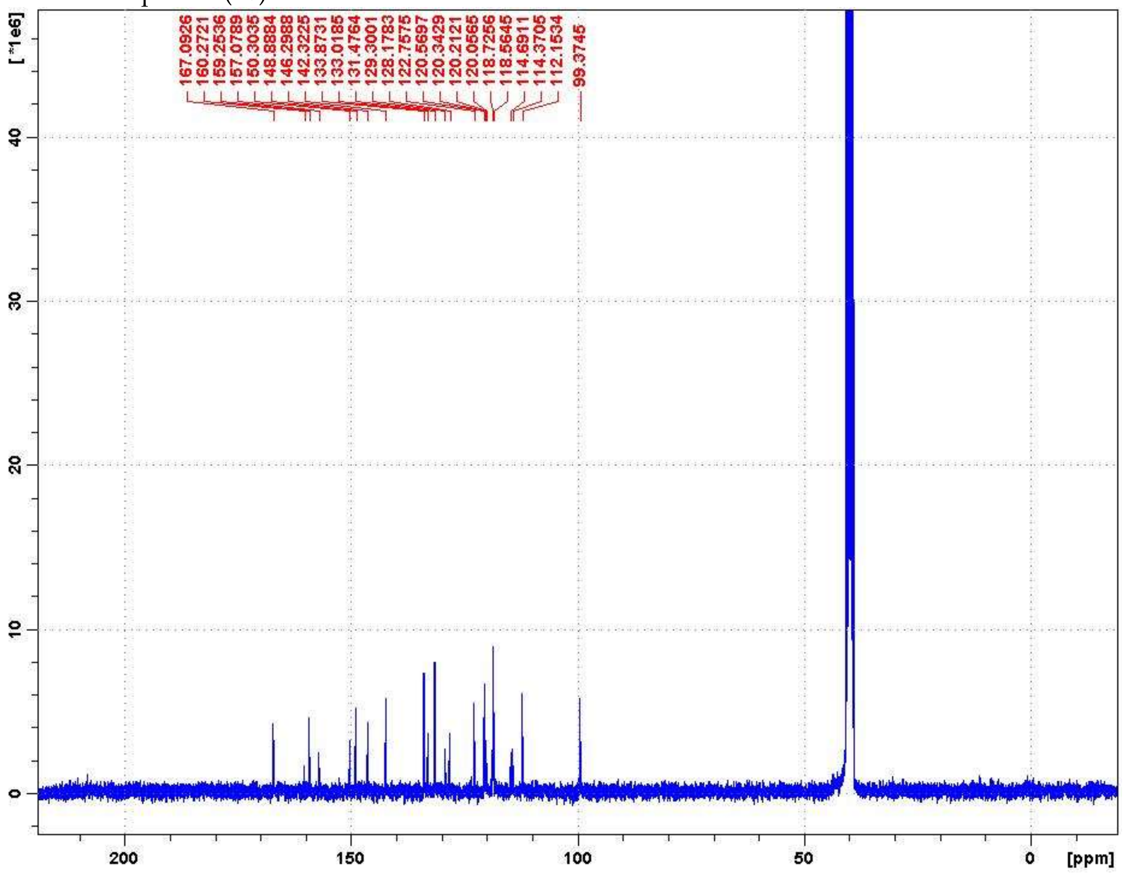

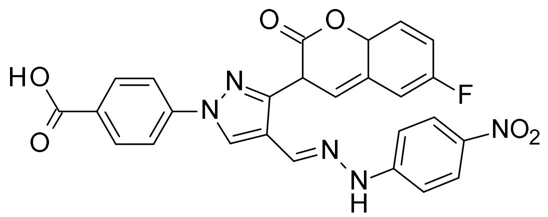

4-[3-(7-fluoro-2-oxo-3,8a-dihydrochromen-3-yl)-4-[(E)-[(4-nitrophenyl)hydrazono]methyl]pyrazol-1-yl]benzoic acid (21). Orange; (458 mg, 89%) ^{1}H NMR (300MHz, DMSO-d$_6$): δ 11.2 (s, 1H), 9.08 (s, 1H), 8.35 (s, 1H), 8.11-8.02 (m, 5H), 7.94 (d, *J* = 9.2 Hz, 2H), 7.75-7.72 (m, 1H), 7.61-7.55 (m, 2H), 6.90-6.87 (m, 2H); ^{13}C NMR (75 MHz, DMSO-d$_6$): δ 167.0, 158.6 (d, ^1J = 239.4 Hz), 159.2, 150.8, 150.3, 146.4, 142.3, 142.2, 138.4, 134.9, 131.4, 129.3, 128.4, 126.3, 122.5, 120.37, 120.32, 120.2, 120.1 (d, ^3J = 10.6 Hz), 118.3, 114.5 (d, ^2J = 24.4 Hz), 111.2. HRMS (ESI-FTMS Mass (m/z): calcd for $C_{26}H_{16}FN_5O_6$ [M+H]$^+$ = 514.1157, found 514.115.

^{1}H NMR of compound (**21**)

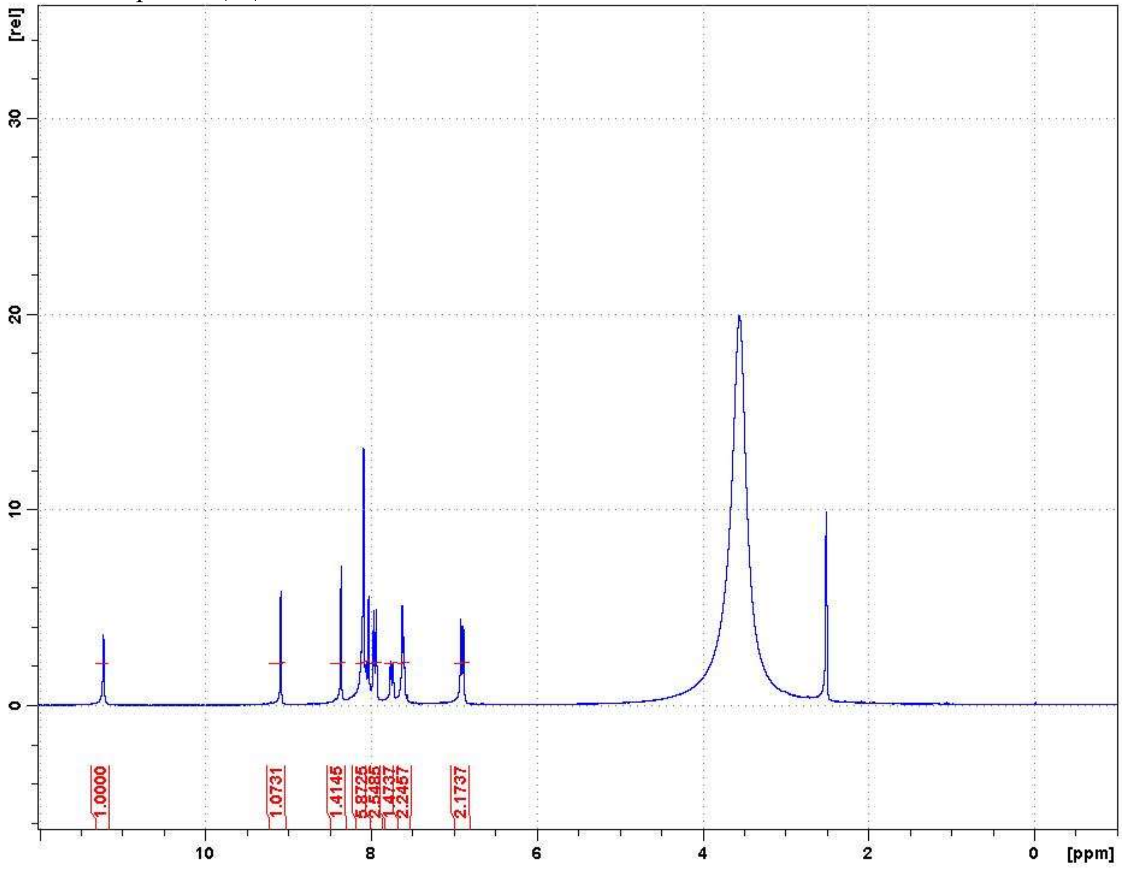

^{13}C NMR of compound (**21**)

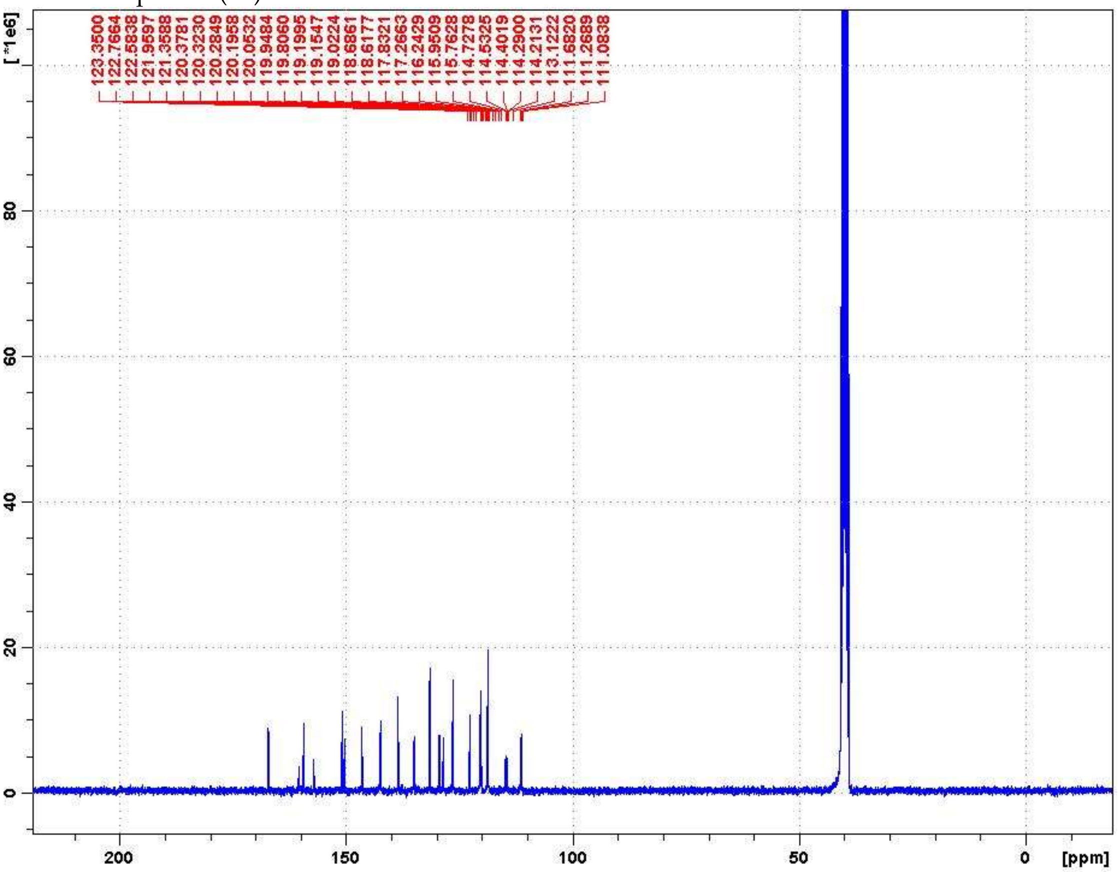

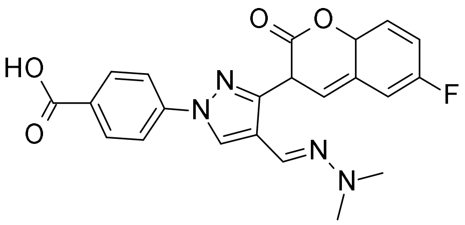

4-[4-[(E)-(dimethylhydrazono)methyl]-3-(6-fluoro-2-oxo-3,8a-dihydrochromen-3-yl)pyrazol-1-yl]benzoic acid (22). Yellow; (333 mg, 79%) ^{1}H NMR (300MHz, DMSO-d_6): 8.75 (s, 1H), 8.27 (s, 1H), 8.12-8.06 (m, 4H), 7.73-7.70 (m, 1H), 7.54 (d, *J* = 6.4 Hz, 2H), 7.20 (s, 1H), 2.49 (s, 3H); ^{13}C NMR (75 MHz, DMSO-d_6): δ 167.1, 158.5 (d, ^1J = 239.1 Hz), 159.1, 150.3, 146.1, 142.5, 141.8, 131.4, 128.8, 125.7, 125.5, 122.9, 122.3, 120.3 (d, ^3J = 9.6 Hz), 119.7 (d, ^2J = 24.8 Hz), 118.5 (d, ^3J = 8.7 Hz), 118.3, 114.3 (d, ^2J = 24.1 Hz), 42.9. HRMS (ESI-FTMS Mass (m/z): calcd for $C_{22}H_{17}FN_4O_4$ $[M+H]^+$ = 421.1307, found 421.1298.

^{1}H NMR of compound (**22**)

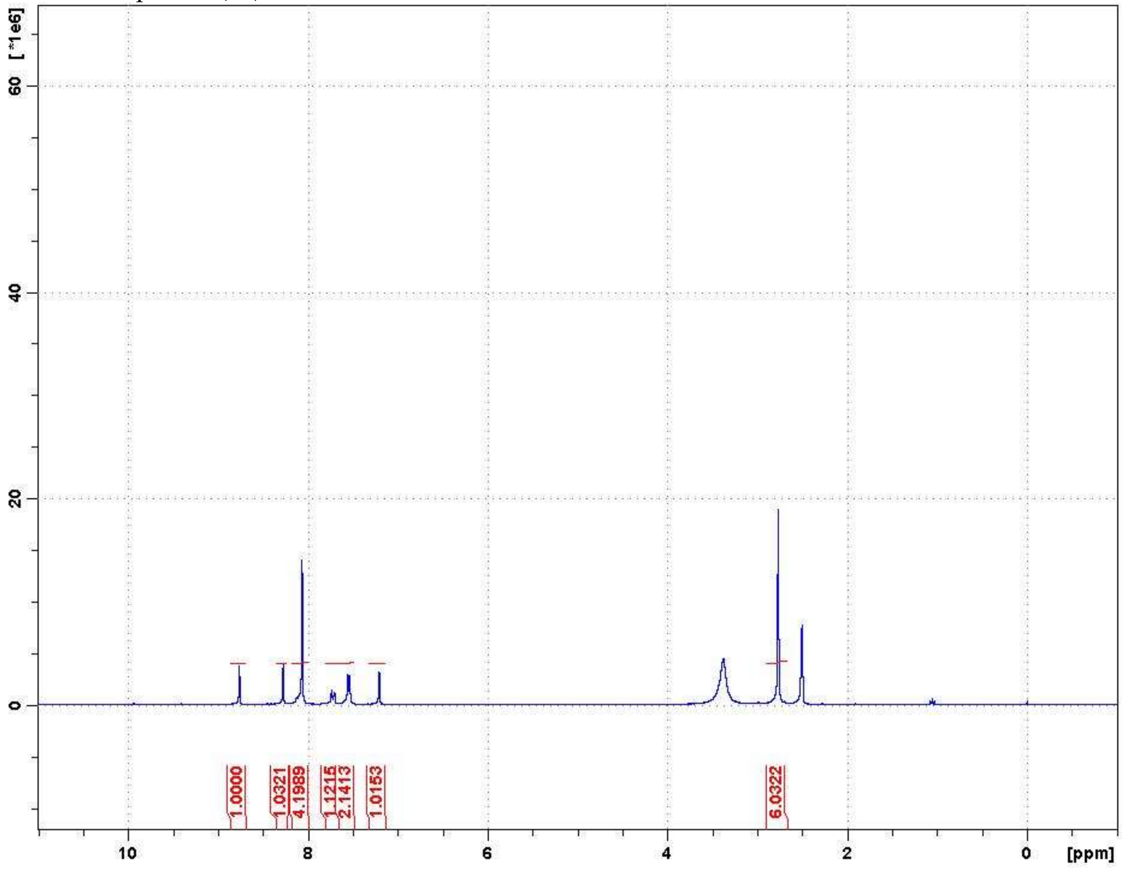

^{13}C NMR of compound (**22**)

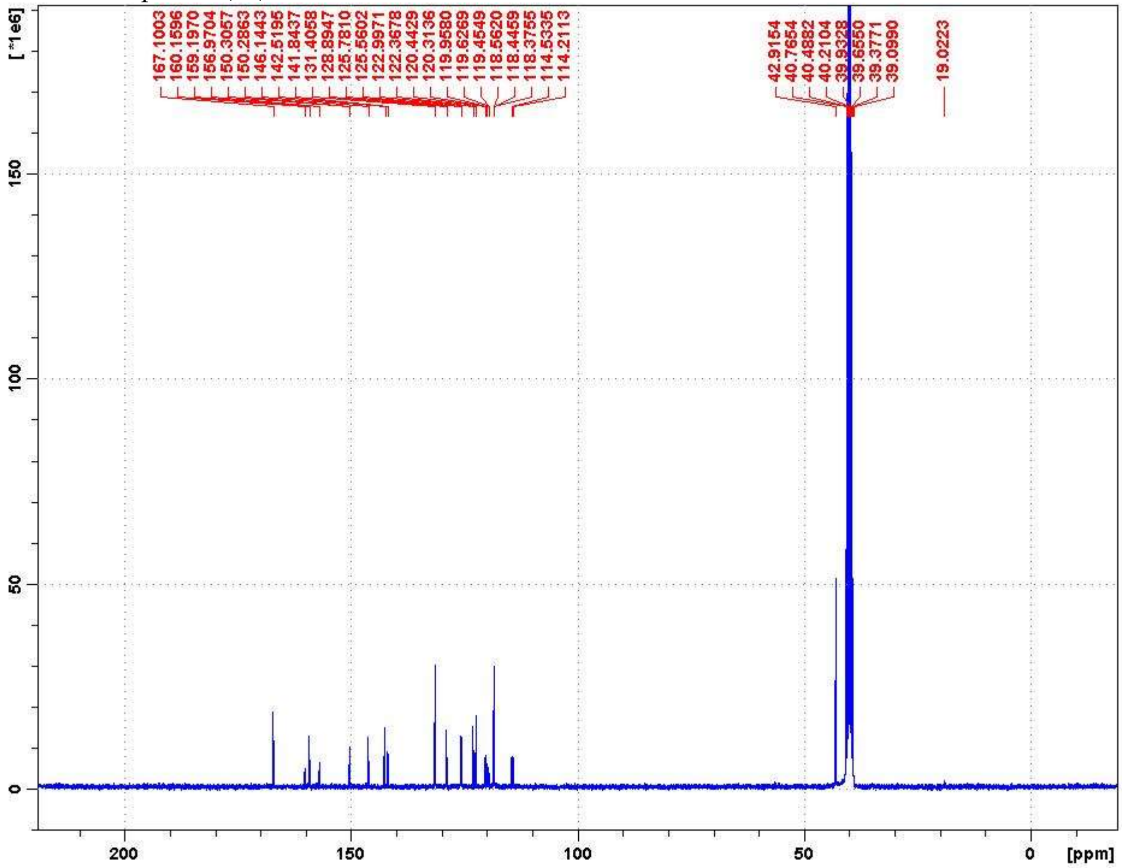

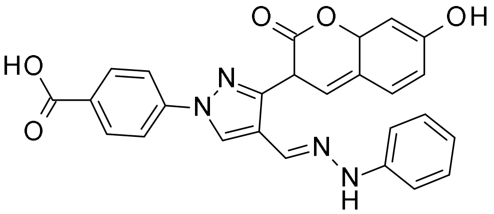

4-[3-(7-hydroxy-2-oxo-chromen-3-yl)-4-[(E)-(phenylhydrazono)methyl]pyrazol-1-yl]benzoic acid (23). Brown; (370 mg, 79%) ^{1}H NMR (300MHz, DMSO-d_6): δ 10.17 (s, 1H), 8.94 (s, 1H), 8.22 (s, 1H), 8.08 (s, 5H), 7.81 (s, 1H), 7.67 (d, *J* = 8.4 Hz, 1H), 7.04 (t, *J* = 7.9 Hz, 2H), 6.83 (t, *J* = 14.7 Hz, 4H), 6.65 (t, *J* = 7.0 Hz, 1H); ^{13}C NMR (75 MHz, DMSO-d_6) δ 167.1, 162.2, 159.9, 155.9, 146.9, 145.7, 143.7, 142.5, 131.4, 130.7, 129.5, 129.3, 128.8, 126.8, 121.2, 118.7, 118.3, 117.0, 114.0, 112.1, 111.8, 102.4. HRMS (ESI-FTMS Mass (m/z): calcd for $C_{26}H_{18}N_4O_5$ $[M+H]^+$ = 467.1350, found 467.1352.

^{1}H NMR of compound (**23**)

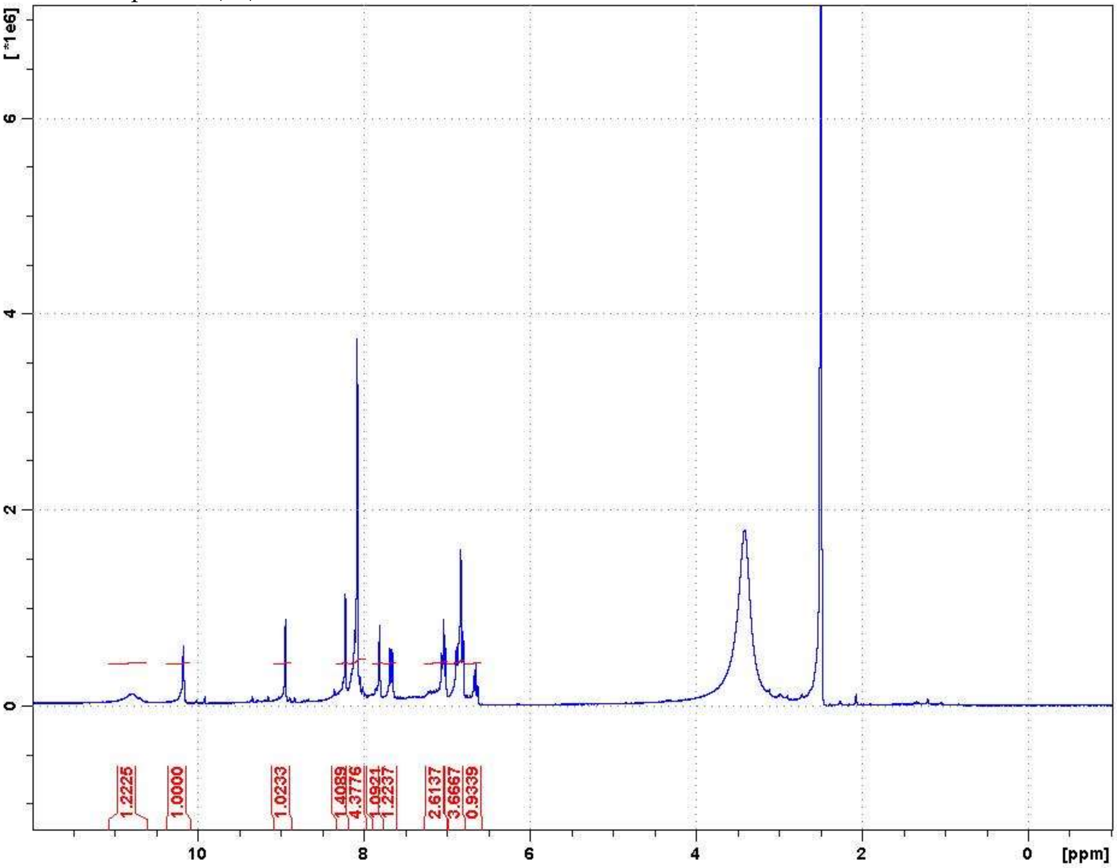

^{13}C NMR of compound (**23**)

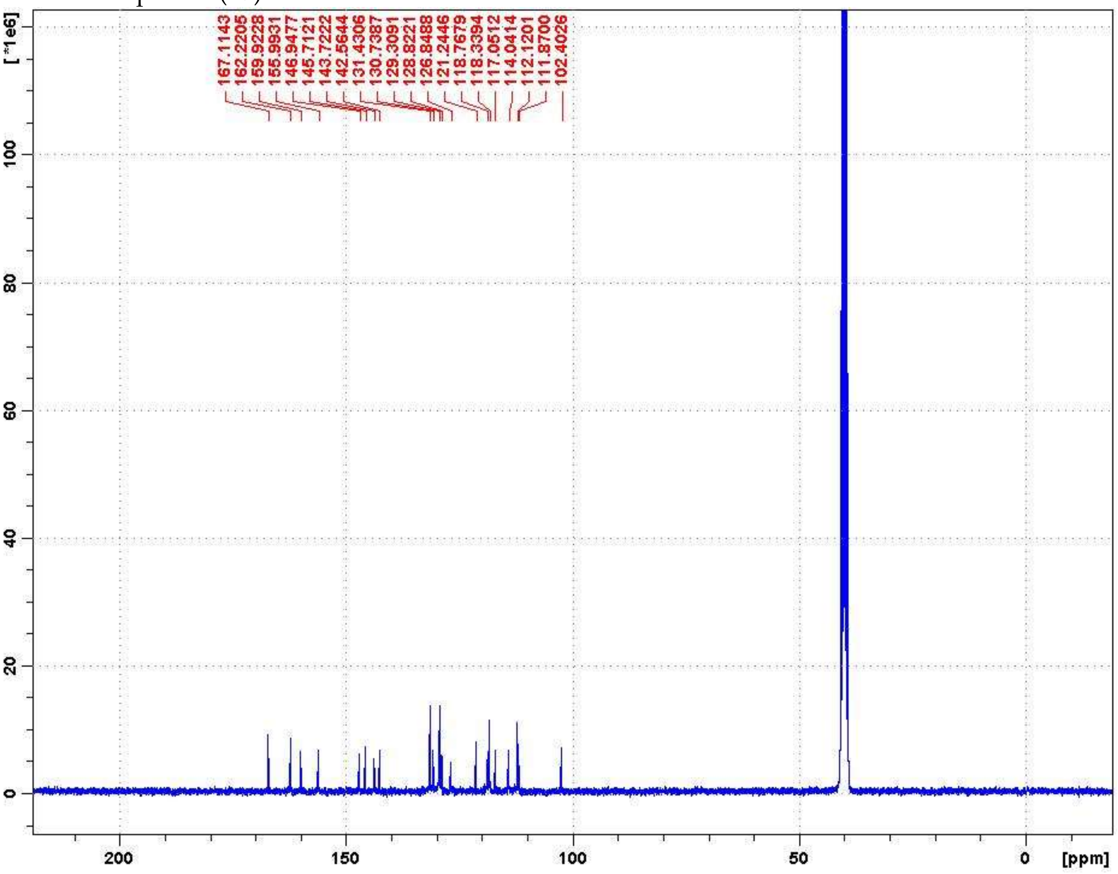

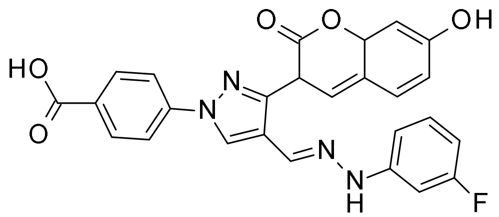

4-[4-[(E)-[(3-fluorophenyl)hydrazono]methyl]-3-(7-hydroxy-2-oxo-chromen-3-yl)pyrazol-1-yl]benzoic acid (24). Brownish; (428 mg, 86%) ^{1}H NMR (300MHz, DMSO-d_6): δ 10.95 (br s, 1H), 10.54 (s, 1H), 9.00 (s, 1H), 8.22 (s, 1H), 8.08 (s, 5H), 7.87 (s, 1H), 7.66 (d, *J* = 8.1 Hz, 1H), 7.07-7.06 (m, 1H), 6.90-6.86 (m, 2H), 6.66-6.60 (m, 2H), 6.41 (t, *J* = 8.1 Hz, 1H); ^{13}C NMR (75 MHz, DMSO-d_6) δ 167.0, 163.8 (d, ^{1}J = 238.9 Hz), 162.4, 159.9, 155.9, 147.7 (d, ^{3}J = 11.0 Hz), 147.0, 143.8, 142.5, 131.4, 130.8, 130.7, 130.6, 128.9, 127.2, 120.9, 118.3, 116.7, 114.1, 111.7, 108.2, 104.7 (d, ^{2}J = 21.7 Hz), 102.4, 98.5 (d, ^{2}J = 26.0 Hz). HRMS (ESI-FTMS Mass (m/z): calcd for $C_{26}H_{17}FN_4O_5$ $[M+H]^+$ = 485.1256, found 485.1257.

^{1}H NMR of compound (**24**)

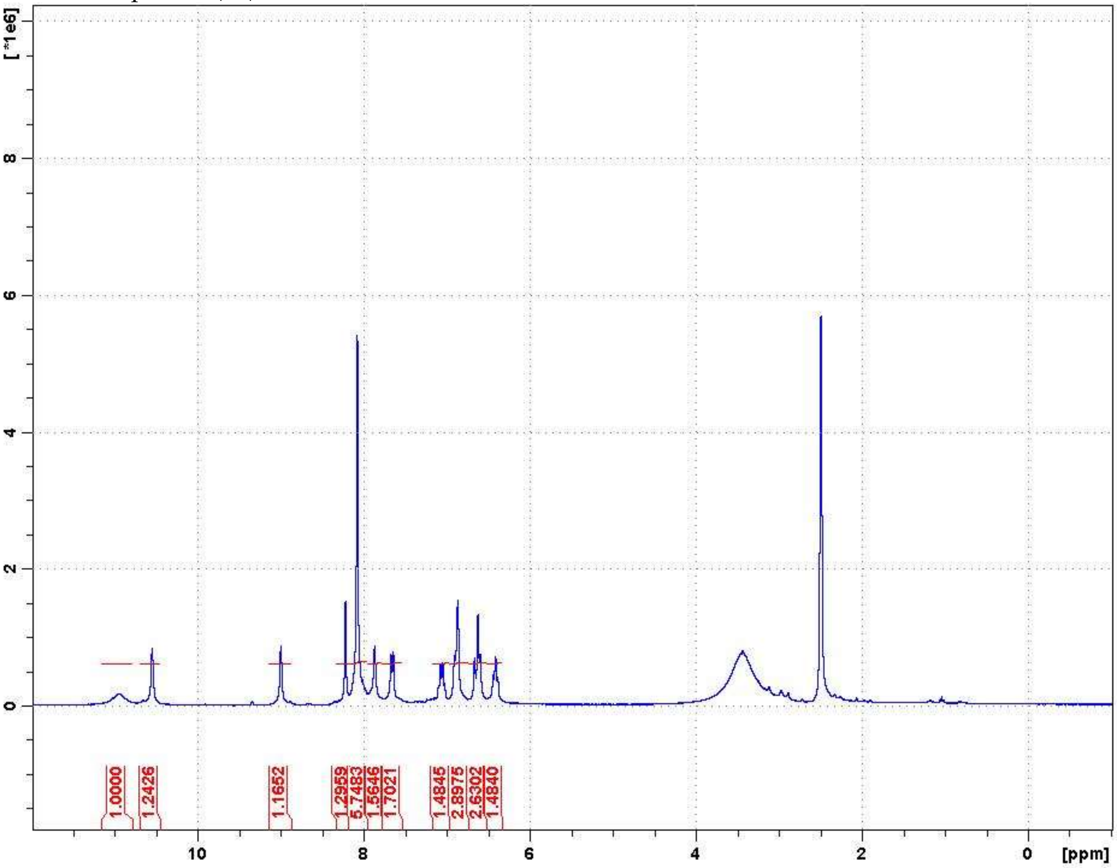

^{13}C NMR of compound (**24**)

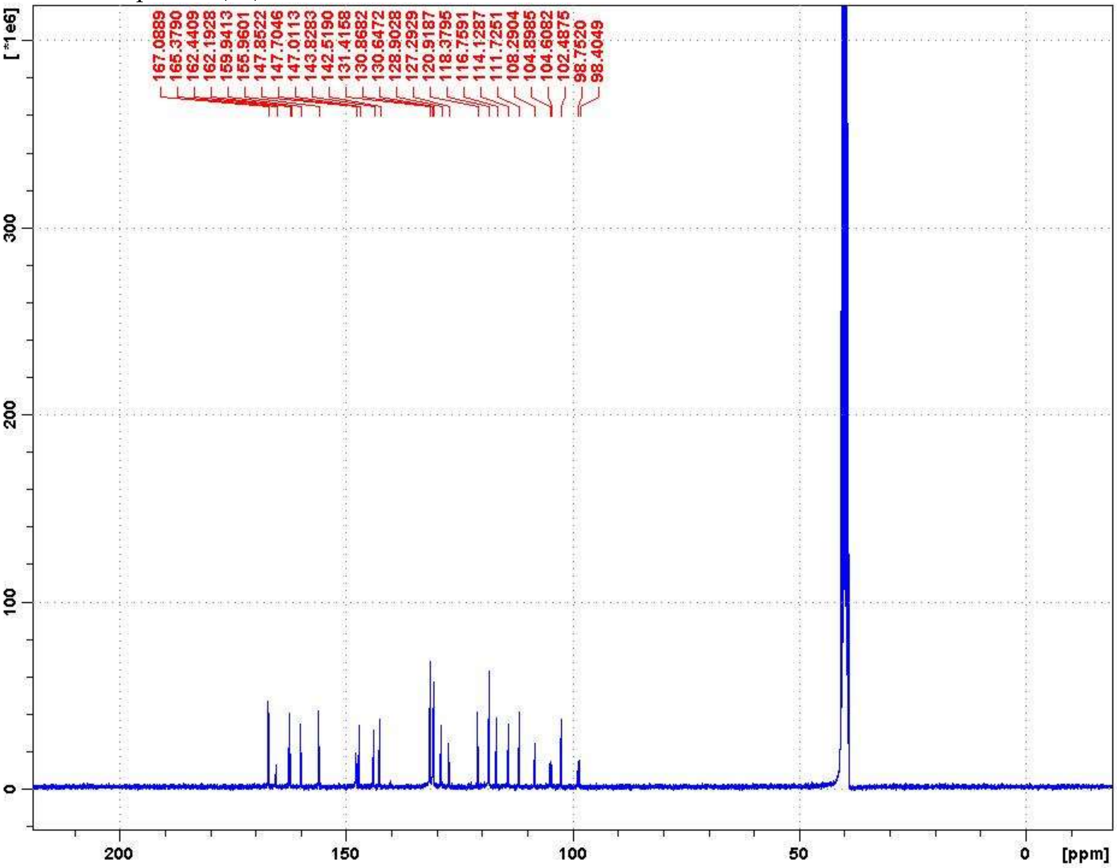

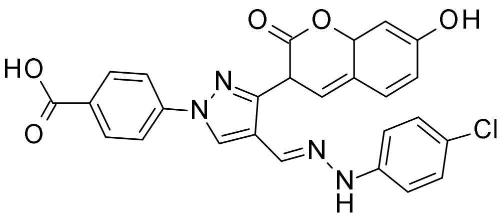

4-[4-[(E)-[(4-chlorophenyl)hydrazono]methyl]-3-(7-hydroxy-2-oxo-chromen-3-yl)pyrazol-1-yl]benzoic acid (25). Brown; (357 mg, 71%) ^{1}H NMR (300MHz, DMSO-d$_6$): δ 10.82 (br s, 1H), 10.32 (s, 1H). 8.95 (s, 1H), 8.22 (s, 1H), 8.06 (s, 4H), 7.80 (s, 1H), 7.66 (d, *J* = 8.2 Hz, 1H), 7.06 (d, *J* = 8.5 Hz, 3H), 6.83-6.81(m, 4H); ^{13}C NMR (75 MHz, DMSO-d$_6$) δ 167.1, 162.2, 159.9, 156.0, 147.0, 144.6, 143.8, 142.5, 131.4, 130.7, 130.4, 129.0, 128.9, 127.0, 121.9, 120.9, 118.3, 116.7, 114.0, 113.5, 111.8, 102.4. HRMS (ESI-FTMS Mass (m/z): calcd for $C_{26}H_{17}ClN_4O_5$ $[M+H]^+$ = 501.0960, 503.0933, found 501.0962, 503.0932.

^{1}H NMR of compound (**25**)

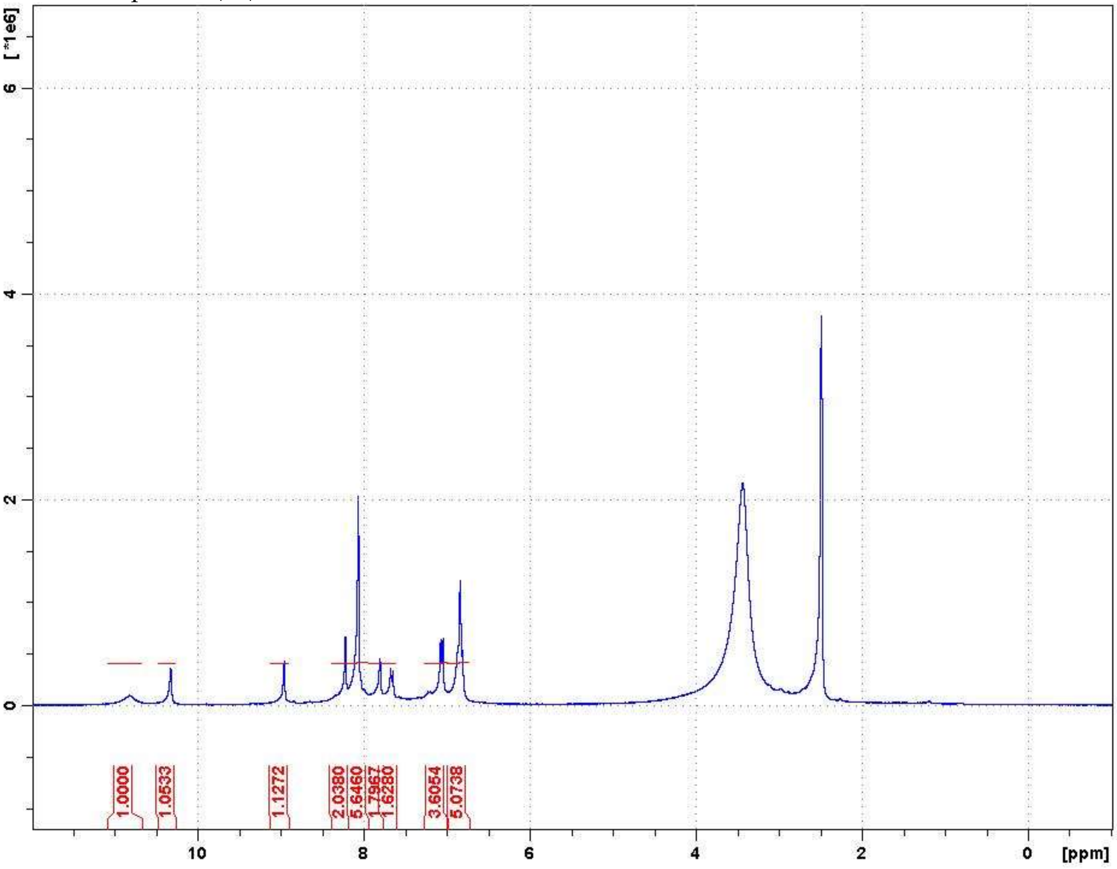

^{13}C NMR of compound (**25**)

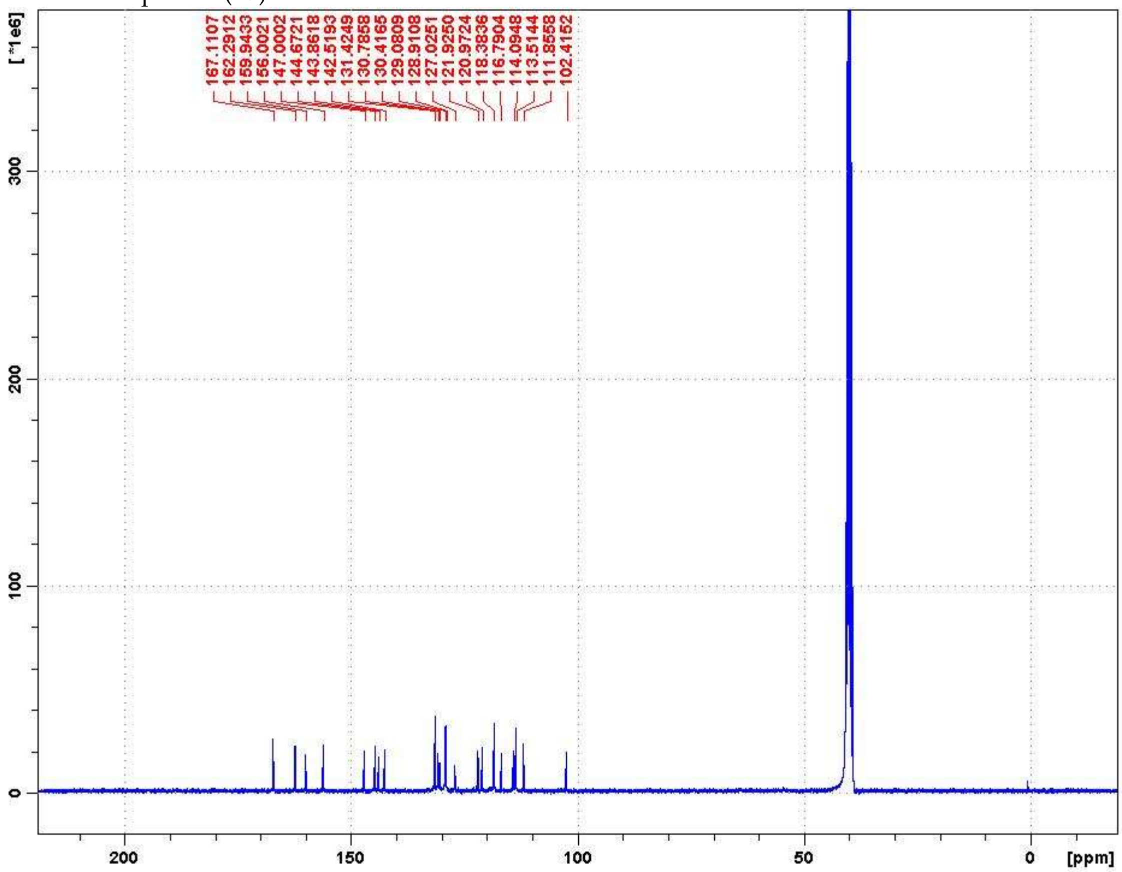

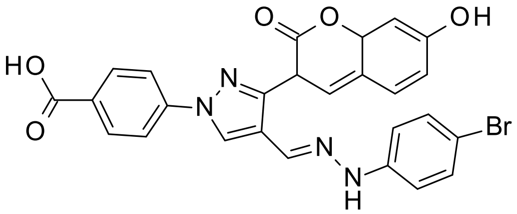

4-[4-[(E)-[(4-bromophenyl)hydrazono]methyl]-3-(7-hydroxy-2-oxo-chromen-3-yl)pyrazol-1-yl]benzoic acid (26). Brown; (437 mg, 80%) ^{1}H NMR (300MHz, DMSO-d$_6$): δ 10.88 (br s, 1H), 10.39 (s, 1H), 8.96 (s, 1H), 8.22 (s, 1H), 8.07 (s, 5H), 7.83 (s, 1H), 7.67 (d, *J* = 7.9 Hz, 1H), 7.18 (d, *J* = 7.8 Hz, 2H), 6.90-6.78 (m, 4H); ^{13}C NMR (75 MHz, DMSO-d$_6$) δ 167.0, 162.3, 159.9, 155.9, 147.0, 145.0, 143.8, 142.5, 131.8, 131.4, 130.7, 130.4, 128.8, 127.0, 120.9, 118.3, 116.7, 114.1, 114.0, 111.8, 109.4, 102.4. HRMS (ESI-FTMS Mass (m/z): calcd for $C_{26}H_{17}BrN_4O_5$ $[M+H]^+$ = 545.0455, 547.0436, found 545.0456, 547.0435.

^{1}H NMR of compound (**26**)

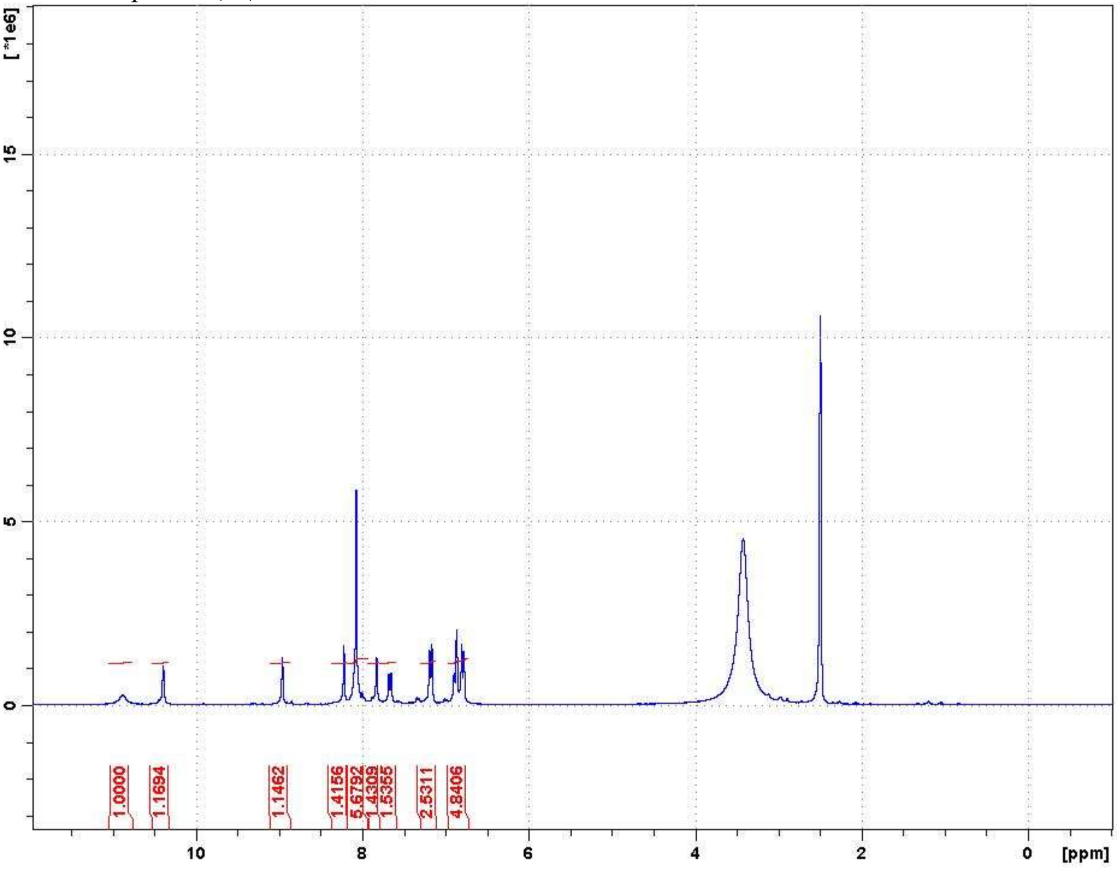

^{13}C NMR of compound (**26**)

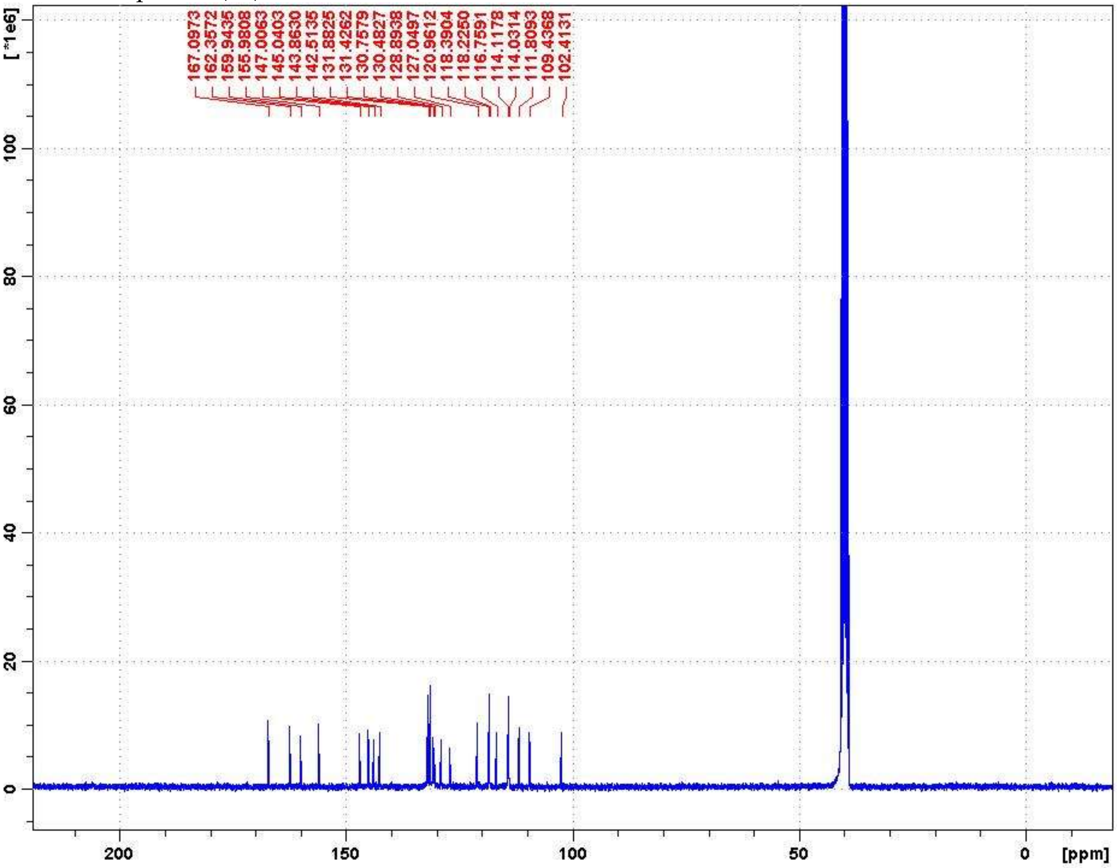

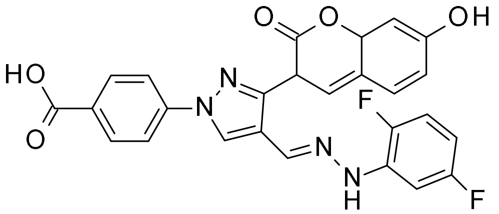

4-[4-[(E)-[(2,5-difluorophenyl)hydrazono]methyl]-3-(7-hydroxy-2-oxo-chromen-3-yl)pyrazol-1-yl]benzoic acid (27). Brownish; (459 mg, 91%) ^{1}H NMR (300MHz, DMSO-d$_6$): δ 10.94 (br s, 1H), 10.33 (s, 1H), 9.03 (s, 1H), 8.20 (s, 1H), 8.13-8.07 (m, 5H), 7.65 (d, J = 8.3 Hz, 1H), 7.12-7.04 (m, 1H), 6.90-6.84 (m, 3H), 6.44-6.39 (m, 1H); ^{13}C NMR (75 MHz, DMSO-d$_6$) δ 167.0, 162.4, 159.7 (d, ^{1}J = 235.9 Hz), 159.8, 155.9, 145.5 (d, ^{1}J = 241.0 Hz), 147.0, 142.4, 135.28 (m), 133.5, 131.4, 130.6, 128.9, 127.7, 120.6, 118.4, 116.6, 116.4, 116.2 (d, ^{3}J = 9.6 Hz), 114.1, 111.6, 103.7 (d, ^{2}J = 25.0 Hz), 102.4, 100.4 (d, ^{2}J = 29.2 Hz). HRMS (ESI-FTMS Mass (m/z): calcd for $C_{26}H_{16}F_2N_4O_5$ [M+H]$^+$ = 503.1162, found 503.1158.

^{1}H NMR of compound (**27**)

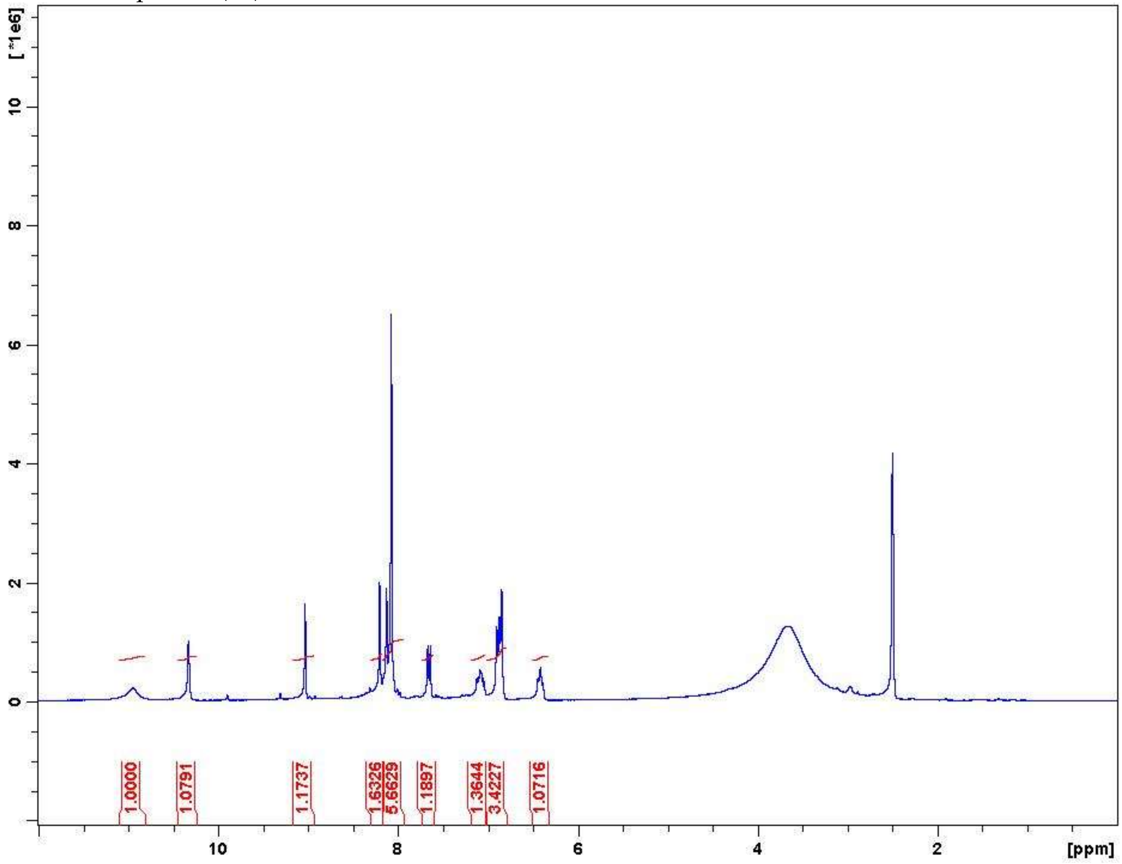

^{13}C NMR of compound (**27**)

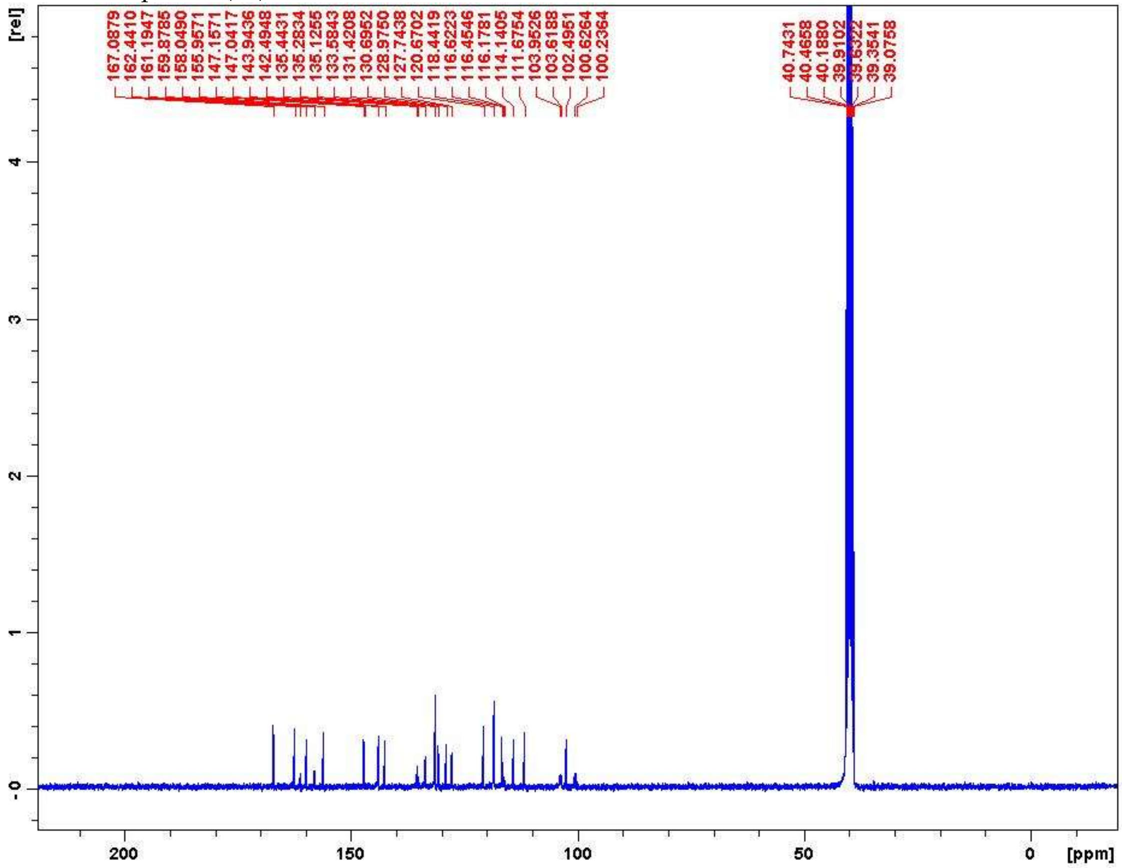

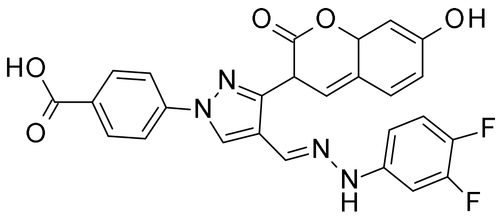

4-[4-[(E)-[(3,4-difluorophenyl)hydrazono]methyl]-3-(7-hydroxy-2-oxo-chromen-3-yl)pyrazol-1-yl]benzoic acid (28). Black; (378 mg, 75%) ^{1}H NMR (300MHz, DMSO-d$_6$): δ 10.41 (s, 1H), 8.99 (s, 1H), 8.22 (s, 1H), 8.07 (s, 4H), 7.83 (s, 1H), 7.66 (d, J = 8.2 Hz, 1H), 7.17-7.07 (m, 1H), 6.88-6.76 (m, 3H), 6.57 (s, 1H); ^{13}C NMR (75 MHz, DMSO-d$_6$) δ 167.1, 162.3, 159.9, 155.9, 152.1 (dd, J = 13.0, 254.0 Hz), 146.9, 144.4 (dd, J = 12.9, 246.3 Hz), 143.8, 142.4, 141.2 (d, ^{3}J = 12.8 Hz), 131.4, 130.7, 128.8, 127.3, 120.8, 119.3, 118.3, 118.0 (d, ^{3}J = 17.5 Hz), 116.7, 114.1, 111.7, 107.7, 102.4, 100.2 (d, ^{2}J = 21.9 Hz). HRMS (ESI-FTMS Mass (m/z): calcd for $C_{26}H_{16}F_2N_4O_5$ [M+H]$^+$ = 503.1162, found 503.1162.

^{1}H NMR of compound (**28**)

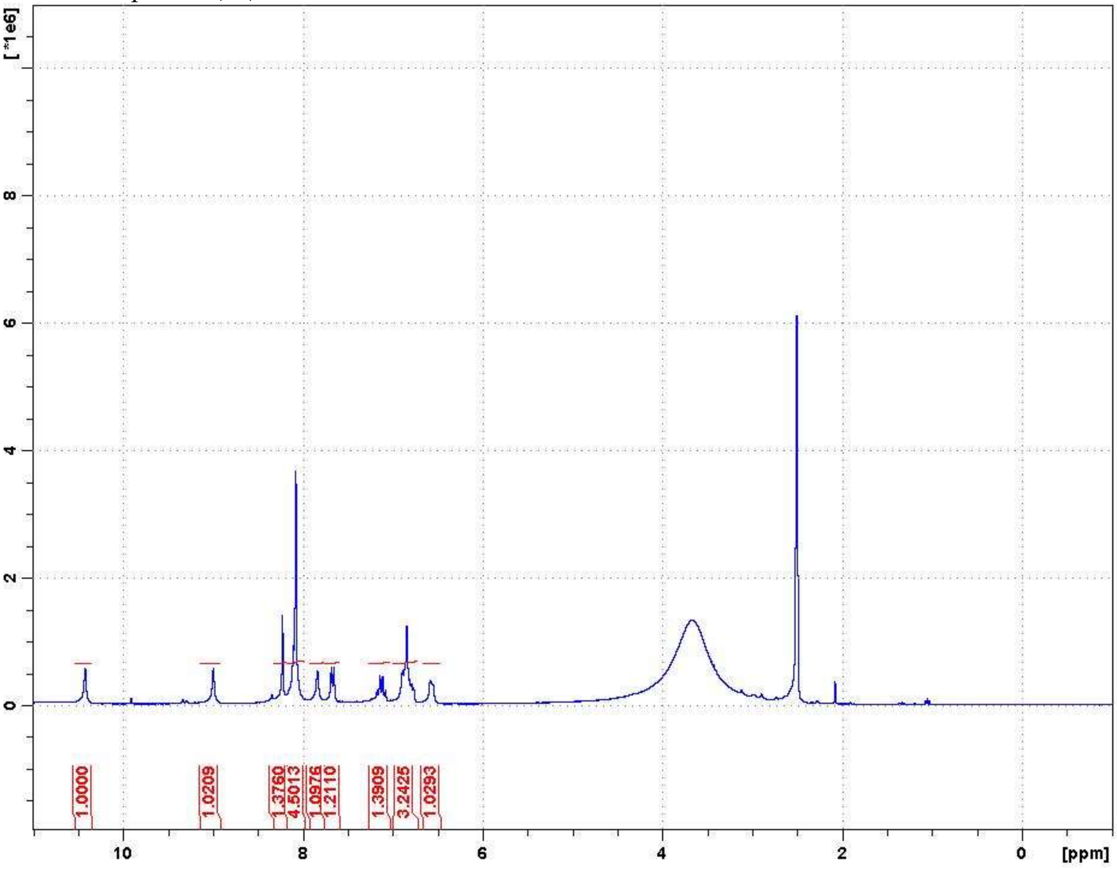

^{13}C NMR of compound (**28**)

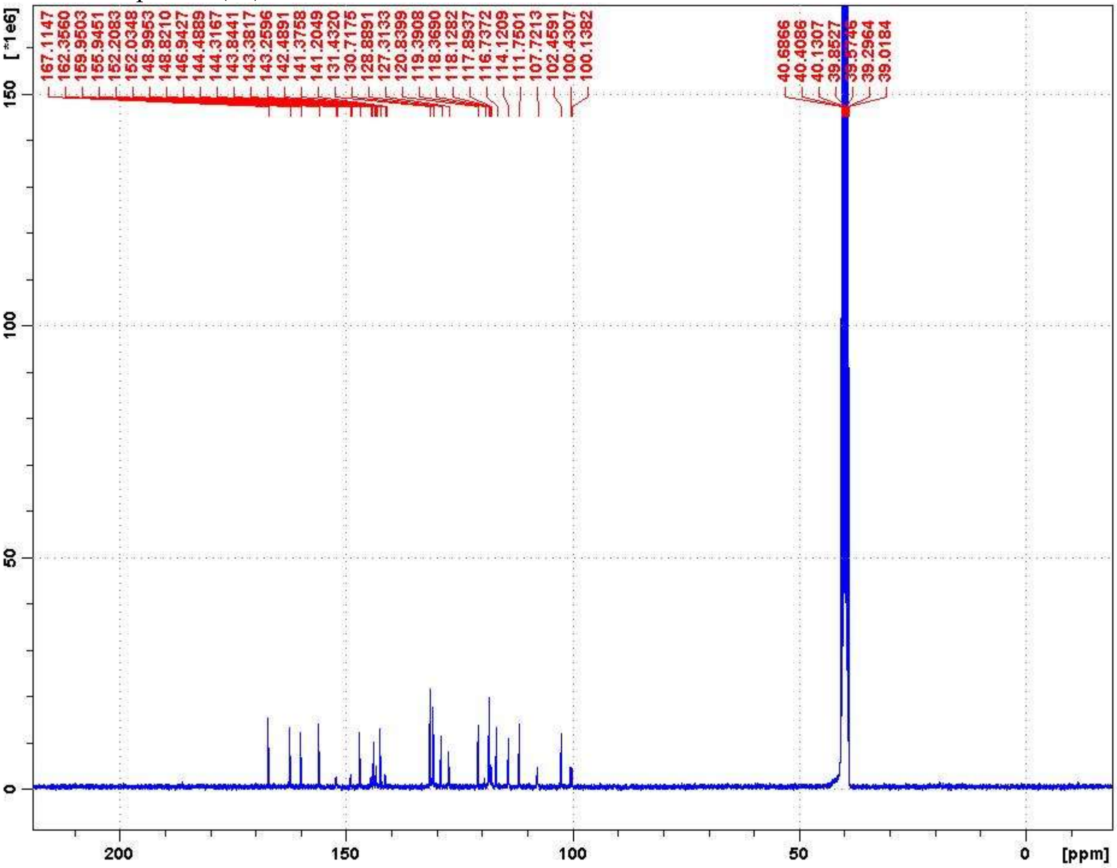

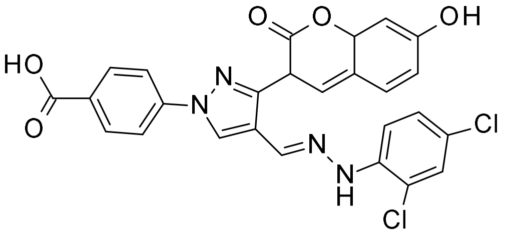

4-[4-[(E)-[(2,4-dichlorophenyl)hydrazono]methyl]-3-(7-hydroxy-2-oxo-chromen-3-yl)pyrazol-1-yl]benzoic acid (29). Brownish; (483 mg, 90%) ^{1}H NMR (300MHz, DMSO-d$_6$): δ 10.94 (br s, 1H), 9.96 (s, 1H), 9.04 (s, 1H), 8.24 (d, *J* = 5.7 Hz, 2H), 8.09 (s, 5H), 7.67 (d, *J* = 8.1 Hz, 1H), 7.40 (s, 1H), 7.23 (d, *J* = 8.7, 1H), 7.00 (d, *J* = 8.9 Hz, 1H) 6.92-6.88 (m, 2H); ^{13}C NMR (75 MHz, DMSO-d$_6$) δ 167.0, 162.4, 159.9, 155.9, 147.3, 144.0, 142.4, 141.0, 134.2, 131.4, 130.7, 128.9, 127.9, 127.3, 122.1, 120.7, 118.4, 116.7, 116.4, 115.0, 114.1, 111.7, 102.4. HRMS (ESI-FTMS Mass (m/z): calcd for $C_{26}H_{16}Cl_2N_4O_5$ $[M+H]^+$ = 535.0571, 537.0543, found 535.0570, 537.0545.

^{1}H NMR of compound (**29**)

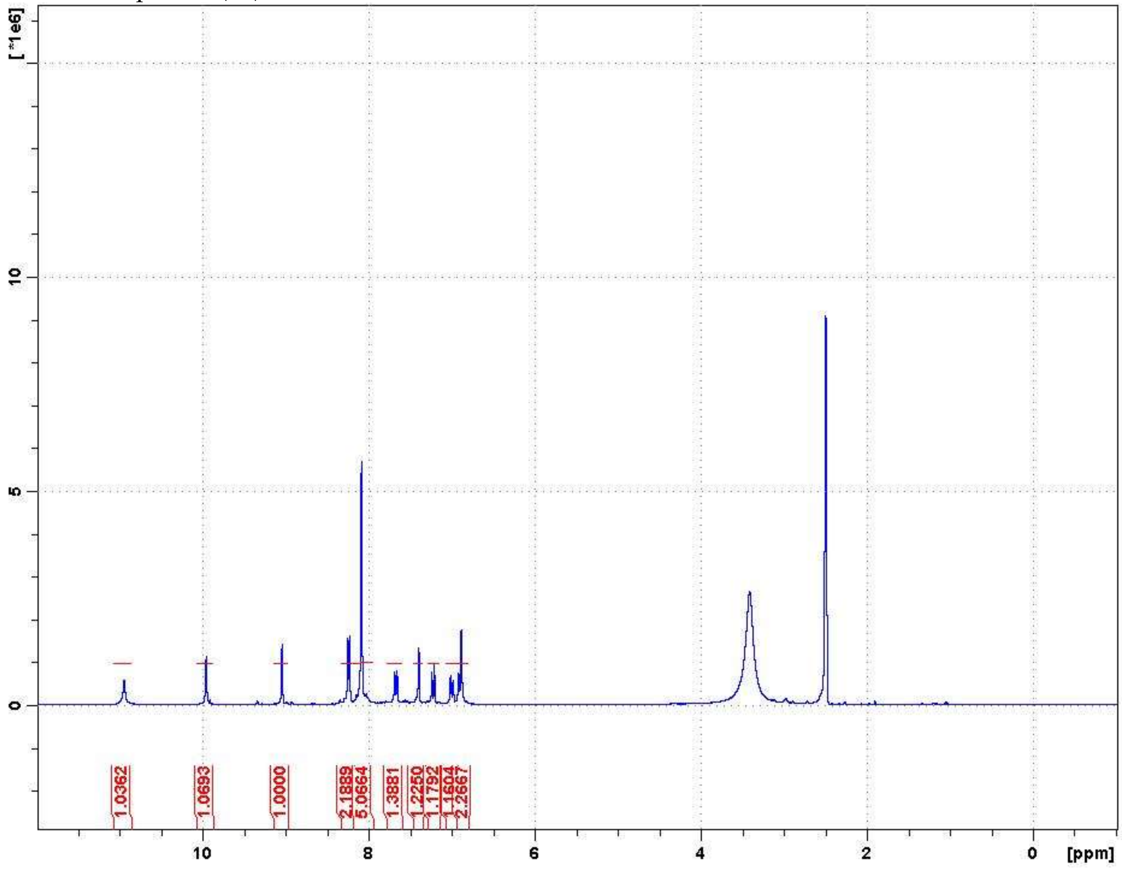

^{13}C NMR of compound (**29**)

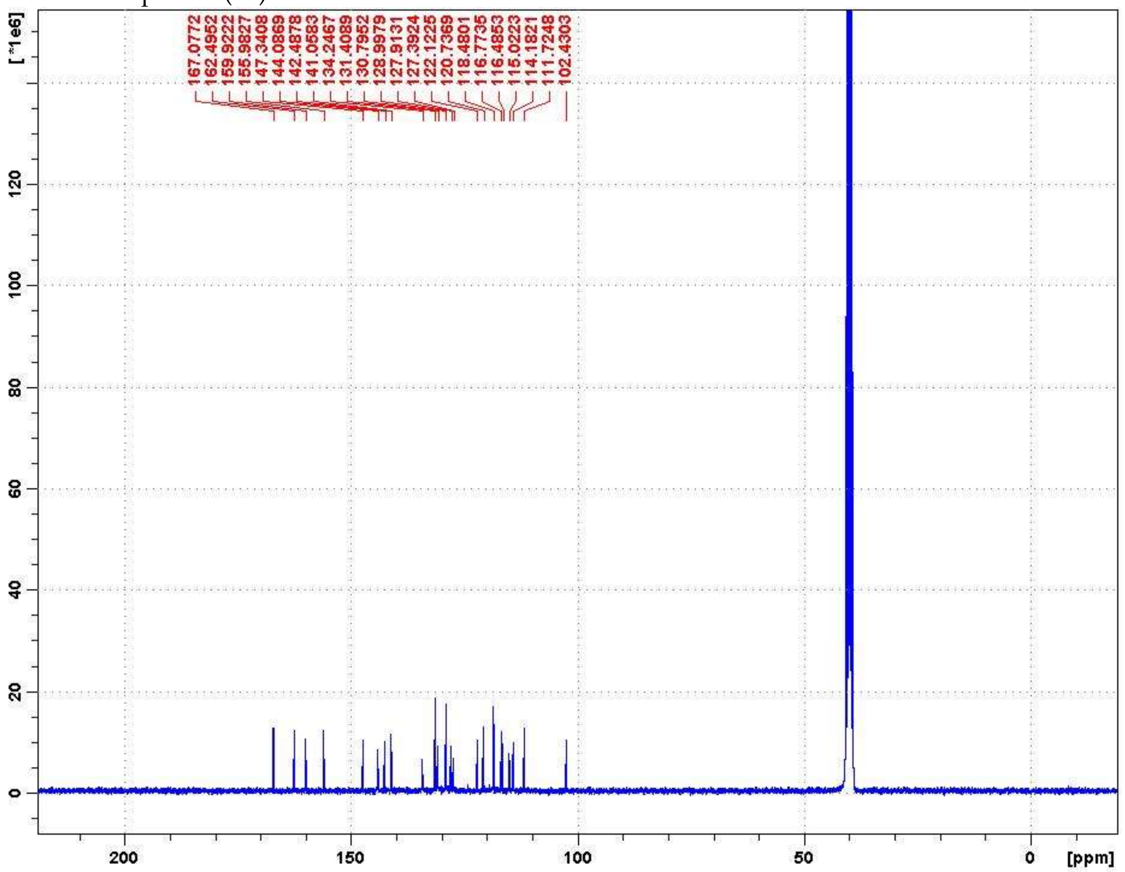

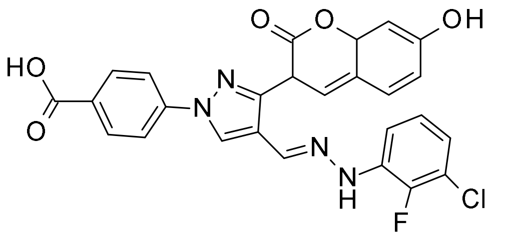

4-[4-[(E)-[(3-chloro-2-fluoro-phenyl)hydrazono]methyl]-3-(7-hydroxy-2-oxo-chromen-3-yl)pyrazol-1-yl]benzoic acid (30). Brown; (437 mg, 84%) ^1H NMR (300MHz, DMSO-d_6): δ 10.33 (s, 1H), 9.00 (s, 1H), 8.32 (s, 1H), 8.11-8.07 (m, 5H), 7.66 (d, *J* = 8.1 Hz, 1H), 7.08 (s, 1H), 6.89-6.79 (m, 4H); ^{13}C NMR (75 MHz, DMSO-d_6) δ 167.1, 162.3, 159.9, 155.9, 147.1, 144.7 (d, ^1J = 240.2 Hz), 143.8, 142.4, 135.3 (d, ^3J = 9.3 Hz), 133.5, 131.4, 130.7, 128.9, 127.5, 125.4, 120.6, 119.9 (d, ^3J = 14.2 Hz), 118.7, 118.4, 116.7, 114.1, 112.6, 111.7, 102.4. HRMS (ESI-FTMS Mass (m/z): calcd for $C_{26}H_{16}ClFN_4O_5$ $[M+H]^+$ = 519.0866, 521.0839, found 519.0869, 521.0844.

^1H NMR of compound (**30**)

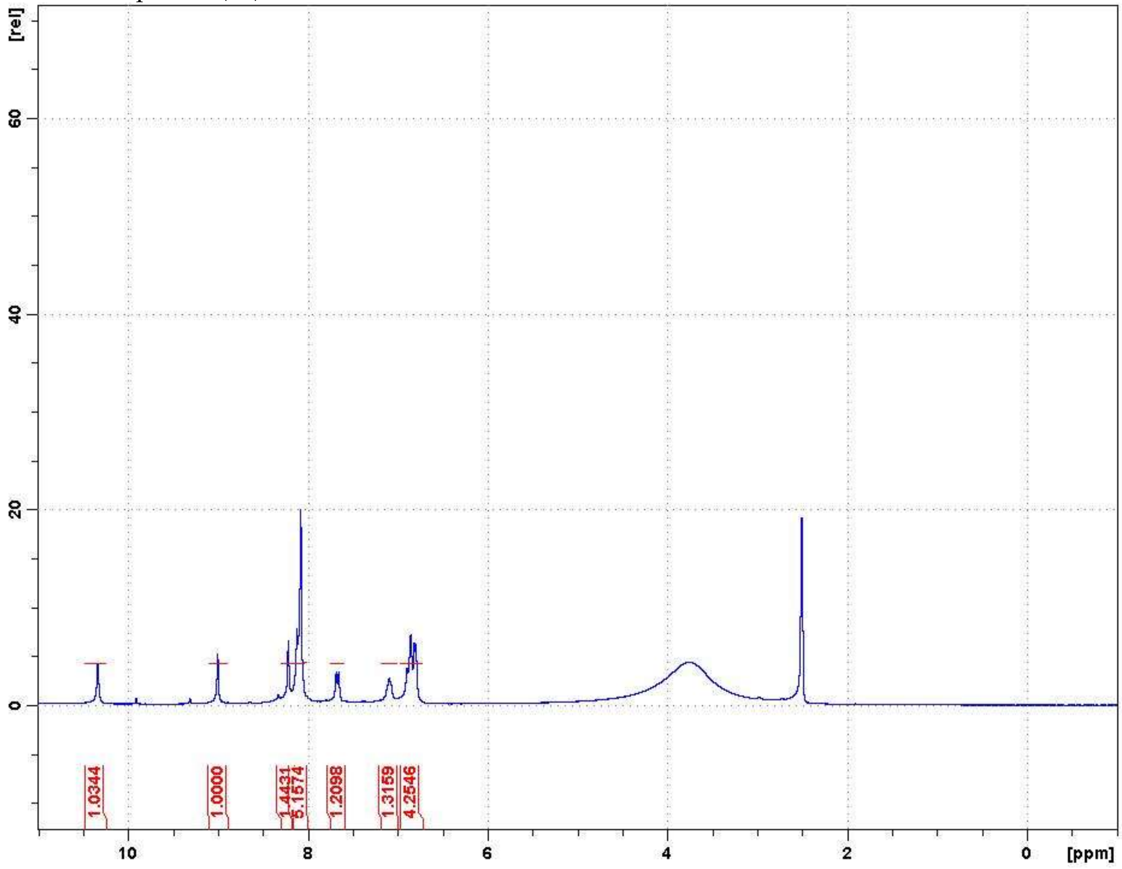

^{13}C NMR of compound (**30**)

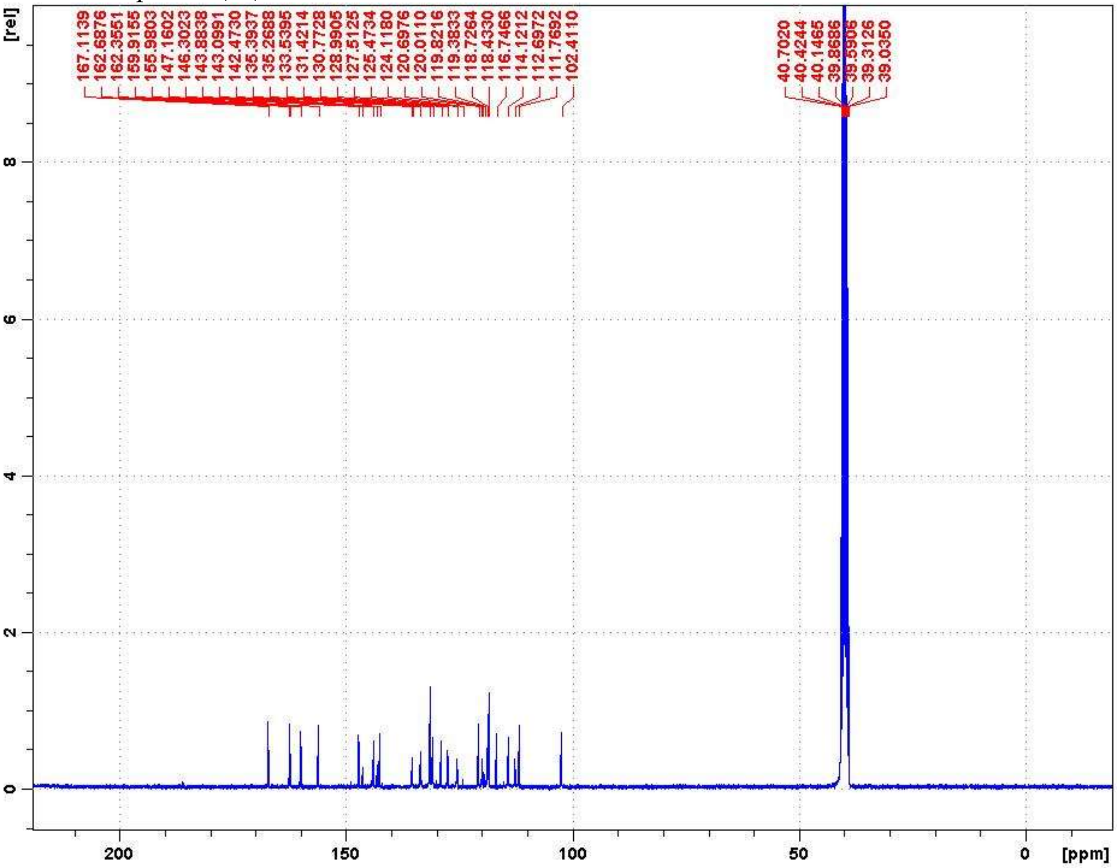

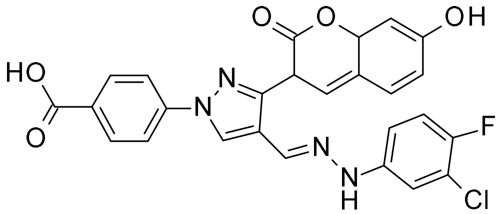

4-[4-[(E)-[(3-chloro-4-fluoro-phenyl)hydrazono]methyl]-3-(7-hydroxy-2-oxo-chromen-3-yl)pyrazol-1-yl]benzoic acid (31). Brown; (380 mg, 73%) ^{1}H NMR (300MHz, DMSO-d$_6$): δ 10.42 (s, 1H), 8.99 (s, 1H), 8.22 (s, 1H), 8.10-8.07 (m, 5H), 7.84 (s, 1H), 7.66 (d, *J* = 7.8 Hz, 1H), 7.12 (t, *J* = 9.5 Hz, 1H), 6.94-6.85 (m, 3H), 6.75 (br s, 1H); ^{13}C NMR (75 MHz, DMSO-d$_6$) δ 167.0, 162.3, 159.9, 155.9, 150.8 (d, ^1J = 234.6 Hz), 146.9, 143.7, 143.2, 142.5, 131.4, 130.7 (d, ^3J = 14.9 Hz), 128.9, 127.4, 120.7, 120.2 (d, ^2J = 18.1 Hz), 119.4, 118.3, 117.4 (d, ^2J = 21.6 Hz), 116.8, 114.0, 112.4, 111.9 (d, ^3J = 6.8 Hz), 111.7, 102.6. HRMS (ESI-FTMS Mass (m/z): calcd for $C_{26}H_{16}ClFN_4O_5$ [M+H]$^+$ = 519.0866, 521.0839, found 519.0866, 521.0837.

^{1}H NMR of compound (**31**)

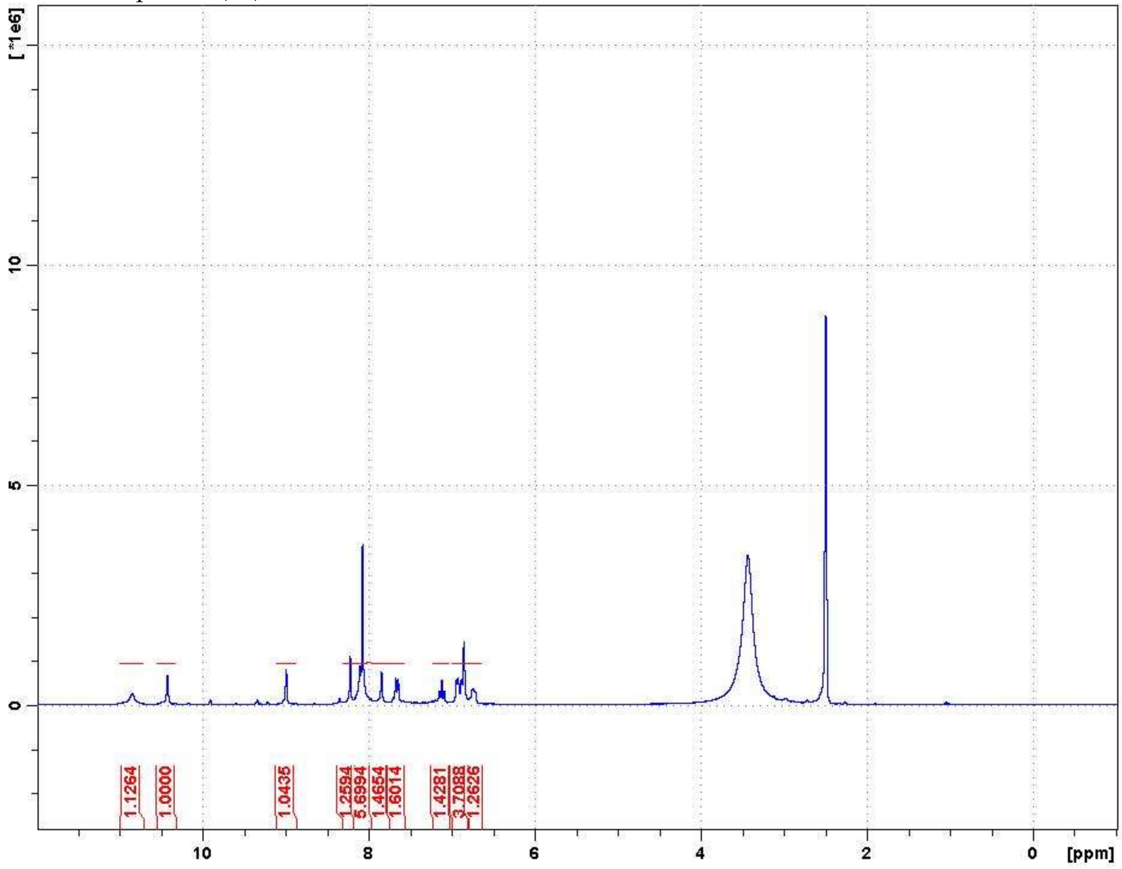

^{13}C NMR of compound (**31**)

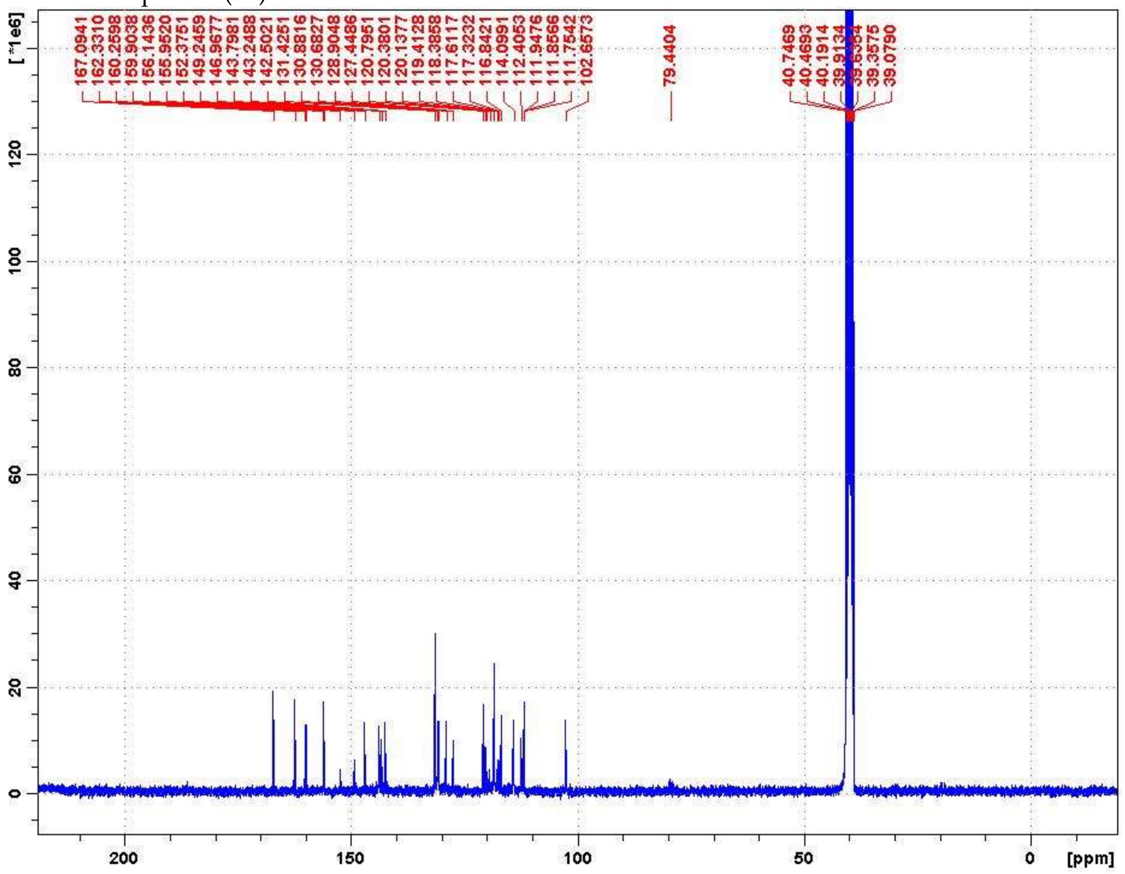

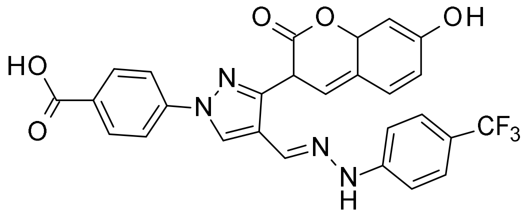

4-[3-(7-hydroxy-2-oxo-3,8a-dihydrochromen-3-yl)-4-[(E)-[[4-(trifluoromethyl)phenyl]hydrazono]methyl]pyrazol-1-yl]benzoic acid (32). Yellow; (445 mg, 83%) ^{1}H NMR (300MHz, DMSO-d$_6$): δ 10.7 (s, 1H), 9.00 (s, 1H), 8.24 (s, 1H), 8.08 (s, 4H), 7.90 (s, 1H), 7.68 (d, *J* = 8.3 Hz, 1H), 7.36 (d, *J* = 8.5 Hz, 1H), 6.96-6.86 (m, 3H); ^{13}C NMR (75 MHz, DMSO-d$_6$) δ 167.1, 162.3, 159.9, 156.0, 148.6, 147.1, 143.9, 142.4, 132.1, 131.4, 130.8, 129.0, 127.4, 127.2, 126.6, 123.6, 120.6, 118.6-118.1 (m), 116.6, 114.1, 111.8, 111.7, 102.3. HRMS (ESI-FTMS Mass (m/z): calcd for $C_{27}H_{17}F_3N_4O_5$ $[M+H]^+$ = 535.1224, found 535.1223.

^{1}H NMR of compound (**32**)

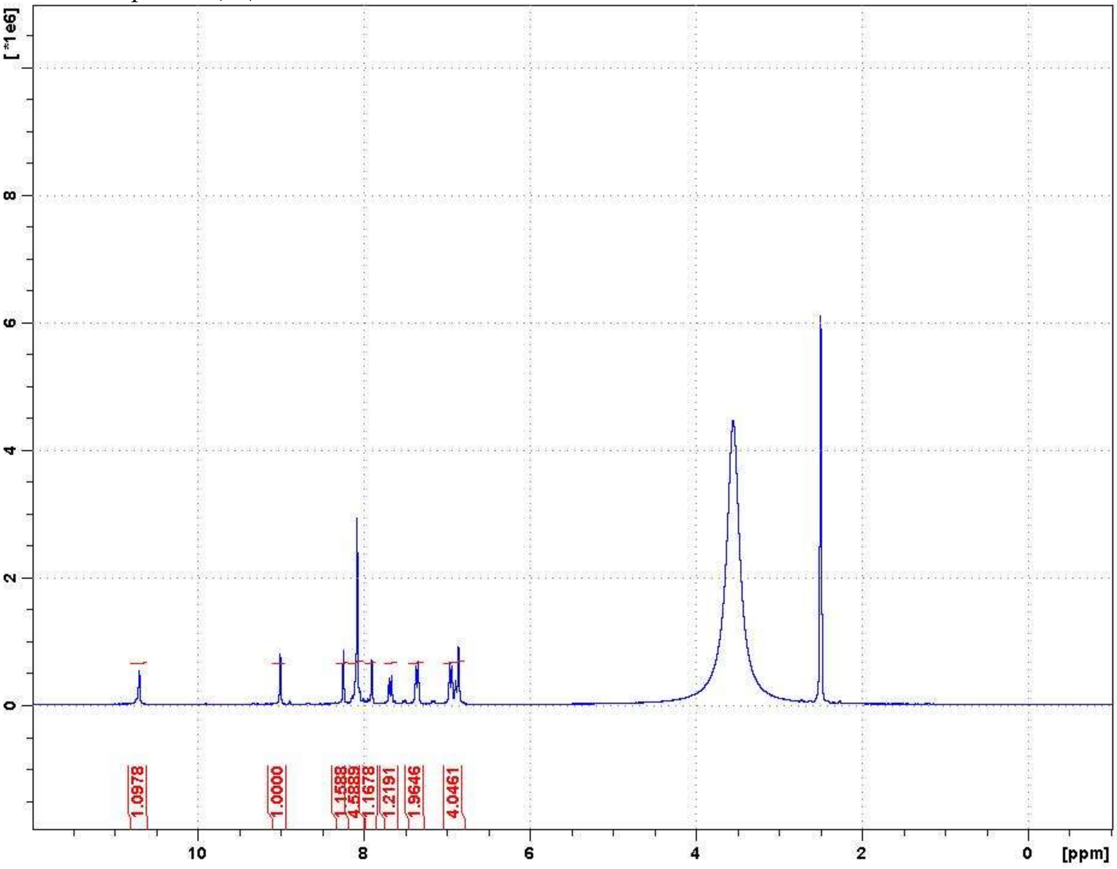

^{13}C NMR of compound (**32**)

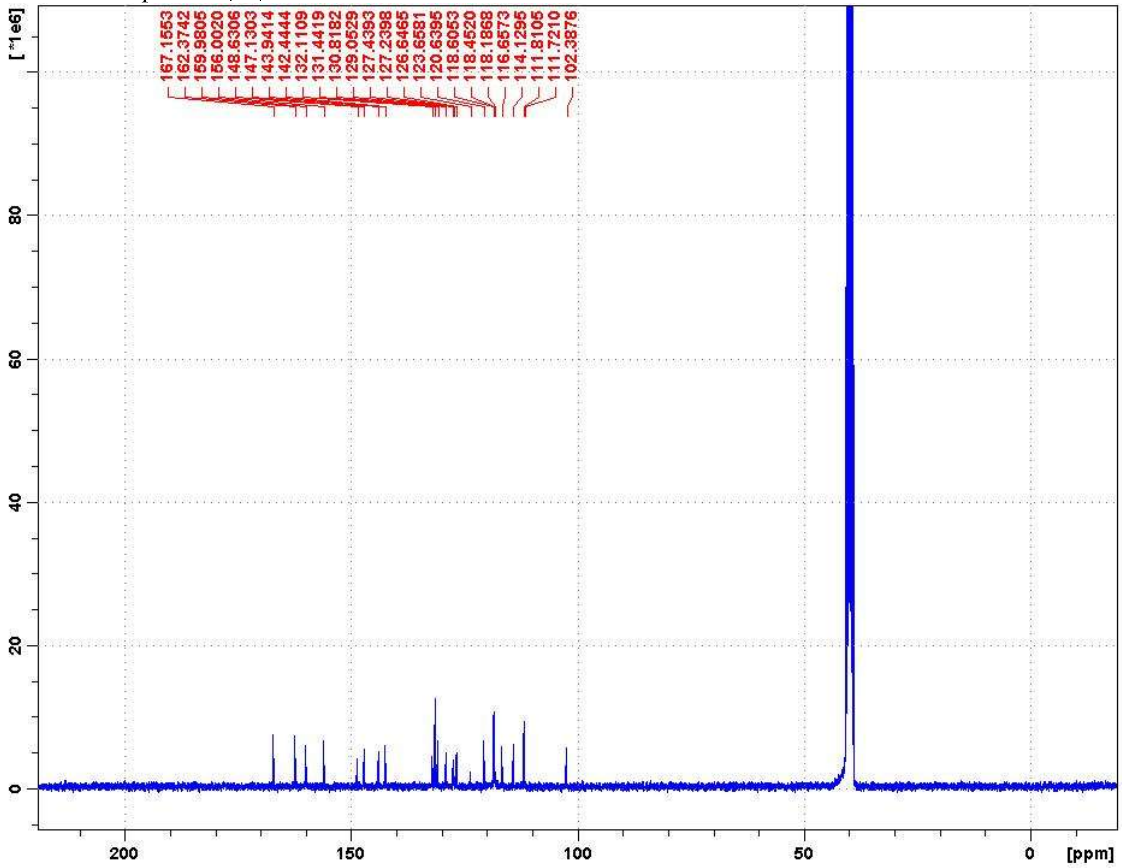

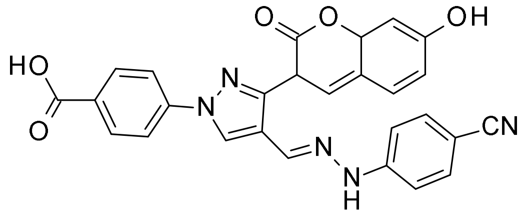

4-[4-[(E)-[(4-cyanophenyl)hydrazono]methyl]-3-(7-hydroxy-2-oxo-3,8a-dihydrochromen-3-yl)pyrazol-1-yl]benzoic acid (33). Yellow; (399 mg, 81%) ^{1}H NMR (300MHz, DMSO-d_6): δ 10.83 (s, 1H), 9.02 (s, 1H), 8.25 (s,1H), 8.08 (s, 4H), 7.92 (s, 1H), 7.67 (d, *J* = 8.3 Hz, 1H), 7.46 (d, *J* = 8.7 Hz, 2H), 6.95-6.85 (m, 4H); ^{13}C NMR (75 MHz, DMSO-d_6) δ 167.1, 162.3, 159.9, 156.0, 148.9, 147.2, 143.9, 142.4, 133.8, 133.3, 131.4, 130.8, 129.0, 127.6, 120.6, 120.4, 118.4, 116.4, 114.1, 112.1, 111.8, 102.4, 99.2. HRMS (ESI-FTMS Mass (m/z): calcd for $C_{27}H_{17}N_5O_5$ $[M+H]^+$ = 492.1302, found 492.1300.

^{1}H NMR of compound (**33**)

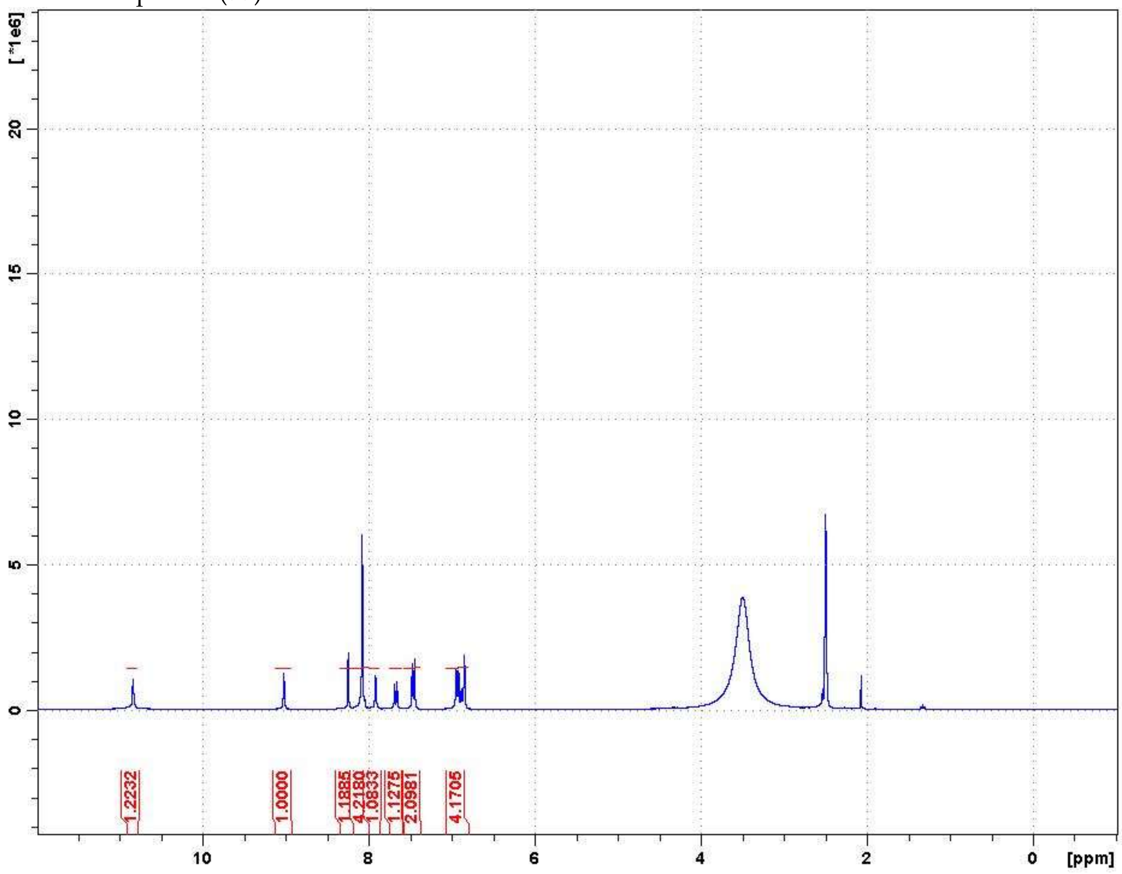

^{13}C NMR of compound (**33**)

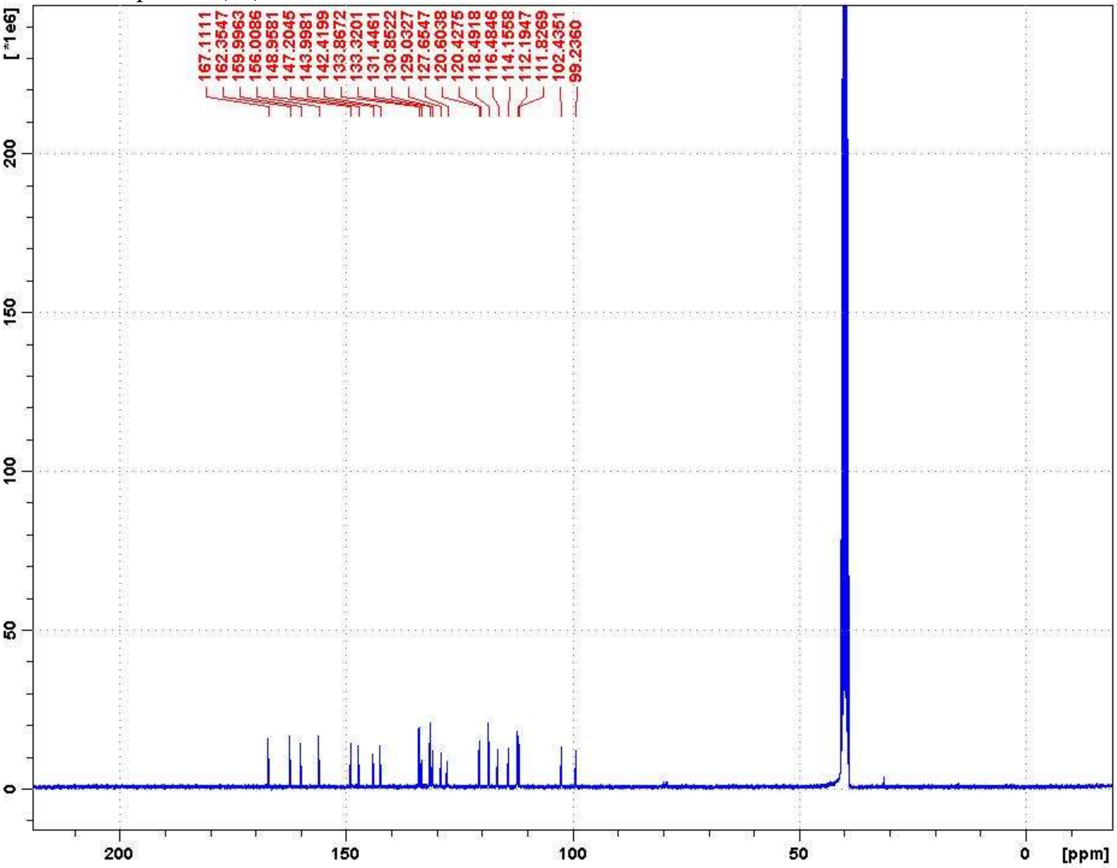

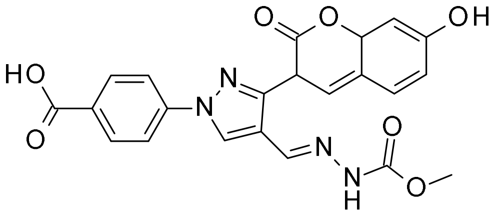

4-[3-(7-hydroxy-2-oxo-3,8a-dihydrochromen-3-yl)-4-[(E)-(methoxycarbonylhydrazono)methyl]pyrazol-1-yl]benzoic acid (34). Brownish; (324 mg, 72%) ^{1}H NMR (300MHz, DMSO-d_6): δ 10.97 (br s, 1H), 8.95 (s, 1H), 8.24 (s, 2H), 8.06 (s, 4H), 7.95 (s, 1H), 7.66 (d, *J* = 8.4 Hz, 1H), 6.87-6.81 (m, 2H); ^{13}C NMR (75 MHz, DMSO-d_6) δ 167.4, 162.6, 160.0, 156.0, 154.2, 147.7, 144.3, 142.0, 131.3, 130.8, 130.3, 127.5, 119.6, 118.9, 118.6, 115.6, 114.1, 111.6, 102.4, 52.2. HRMS (ESI-FTMS Mass (m/z): calcd for $C_{22}H_{16}N_4O_7$ $[M+H]^+$ = 449.1092, found 449.1107.

^{1}H NMR of compound (**34**)

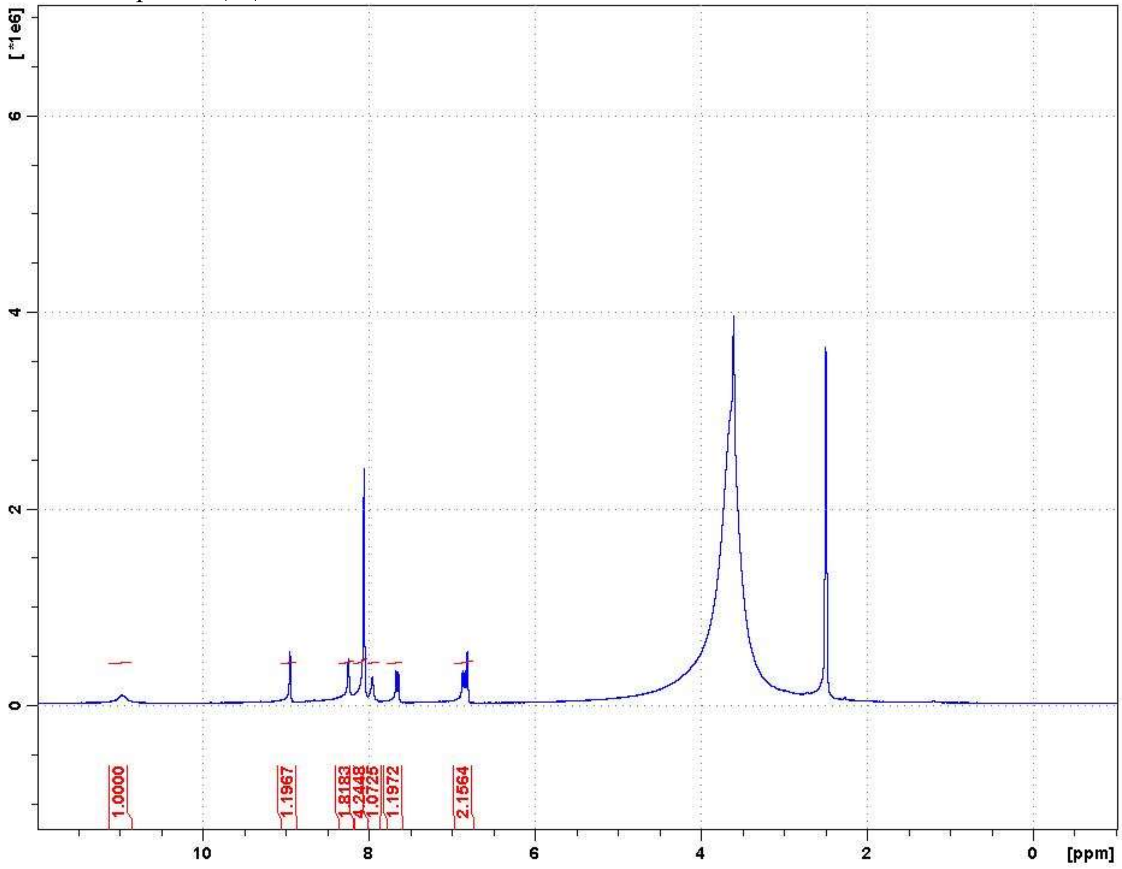

^{13}C NMR of compound (**34**)

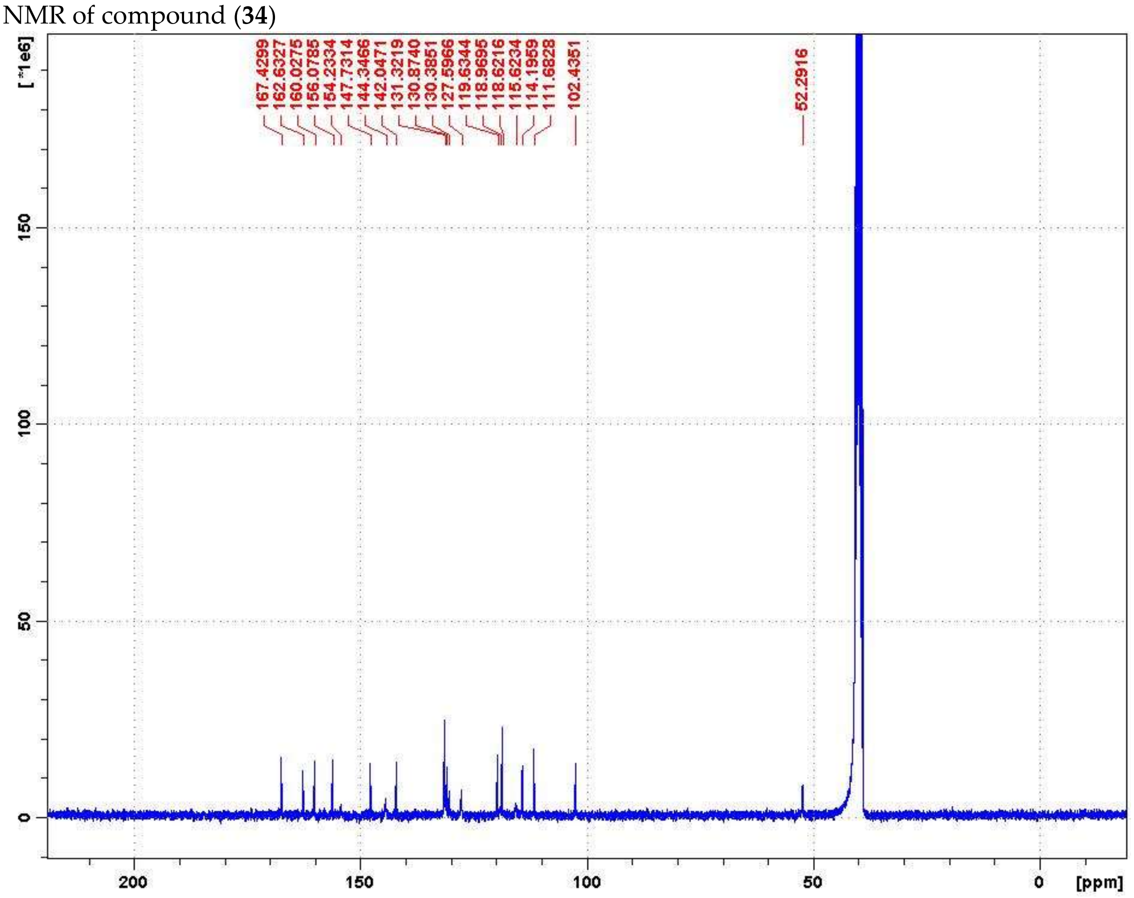

Supplement: Supplementary file 1 [file molecules-25-02758-s001.pdf]
